# Effector loss and gain drives host range at a fitness cost

**DOI:** 10.1371/journal.ppat.1014145

**Published:** 2026-08-03

**Authors:** Marcus V. Merfa, Tracy E. Hawk, Jelmer W. Poelstra, Lillian Ebeling-Koning, Ervan Rodgers, Hannah Toth, Zachary Konkel, Jules Butchacas, Nathaniel Heiden, Muhammad Uzair, Temitope F. Oladele, Godwin E. Oduokpaha, Rebecca D. Curland, Emmanuelle Lauber, Laurie Marsan, Zhaohui Liu, Ruth Dill-Macky, Laurent D. Noël, Horacio D. Lopez-Nicora, Jason C. Slot, Verónica Román-Reyna, Jonathan M. Jacobs

**Affiliations:** 1 Department of Plant Pathology, The Ohio State University, Columbus, Ohio, United States of America; 2 Infectious Disease Institute, The Ohio State University, Columbus, Ohio, United States of America; 3 Department of Microbiology, University of Tennessee, Knoxville, Tennessee, United States of America; 4 Molecular and Cellular Imaging Center, The Ohio State University, Wooster, Ohio, United States of America; 5 The Bredesen Center for Interdisciplinary Research and Graduate Education, University of Tennessee, Knoxville, Tennessee, United States of America; 6 Department of Plant Pathology, University of Minnesota, St. Paul, Minnesota, United States of America; 7 LIPME, Université de Toulouse, INRAE, CNRS, Castanet-Tolosan, France; 8 Department of Plant Pathology, North Dakota State University, Fargo, North Dakota, United States of America; 9 Department of Plant Pathology, University of Wisconsin–Madison, Madison, Wisconsin, United States of America; University of California Riverside, UNITED STATES OF AMERICA

## Abstract

Epidemic preparedness depends on tracking microbial evolution that drives shifts in ecological behaviors such as disease emergence. However, the genetic constraints mediating microbial emergence for generalist and specialist behaviors remain poorly described. Here, we addressed this question by combining comparative and functional genomics with phylogeny-based evolutionary analyses of the cereal pathogen *Xanthomonas translucens*. We show that a generalist *X. translucens* subgroup arose from a specialist ancestor, and the loss of a single effector gene, *xopAL1*, contributed to the generalist host expansion by promoting host jump from barley to wheat. Deleting barley-specialist *X. translucens xopAL1* recapitulated the host jump to wheat and demonstrates risk across each globally distributed genetic lineage. However, this niche expansion via XopAL1 loss incurs a significant fitness cost to colonize barley. Moreover, the specialist lineage gained an additional effector gene, *xopAJ*, which enhanced virulence on barley while restricting oat infection, thereby reinforcing niche specialization. We further conducted transcriptomic analysis of wheat and determined that XopAL1 triggers a defense response that involves the reduction of photosynthetic processes. Our work provides an experimentally validated evolutionary framework to understand mechanisms of intergenera host jump. Overall, we demonstrate that single events of gene loss and gain shape ecological behaviors by creating a dynamic trade-off between niche breadth and specialization.

## Author summary

Pathogenic microorganisms emerge and adapt to new niches posing problems for host health, such as when disease outbreaks occur. Microorganisms may quickly adapt by modifying their genome contents, but the evolutionary mechanisms for historical events like host jump and their impact on potential future epidemics remain poorly understood. Our study shows that loss and gain of secreted pathogen virulence factors (i.e., effectors) alter the host range of the plant pathogenic bacterium *Xanthomonas translucens*. Our combined evolutionary reconstruction of the events together with functional genomics and transcriptomic analyses demonstrate the fitness trade-off of effector presence and absence with pathogenicity and host recognition. In other words, loss of effector genes allowed for host jump from barley to wheat in *X. translucens*, presumably by disrupting pathogen recognition by the host immune system. However, this also reduced the pathogenic fitness of the pathogen in barley. We determined that current pathogen lineages pose risk for host jump and thus provide an informed, testable framework for epidemiological tracking. In essence, understanding the genetic mechanisms of host jump provides key knowledge for proactive prediction and mitigation of disease outbreaks.

## Introduction

Disease outbreaks often result from pathogen evolution, leading to changes in microbial ecological behaviors such as niche specialization. Changes in niche-specificity may occur from single genetic loss or gain events [1,2] or the movement of genomic islands and plasmids that dictate the transitional behaviors such as niche shift between microbial pathogens and their hosts [3]. Niche alterations are universal and widespread in plant, human and animal epidemiology and have significant effects on host health outcomes [4]. Some pathogens have generalist behaviors with broad host ranges while others are highly specialized on a particular host or niche [5]. However, the underlying evolutionary and biological mechanisms often remain undefined for most niche changes, especially in plant agricultural systems.

**Fig 1:**
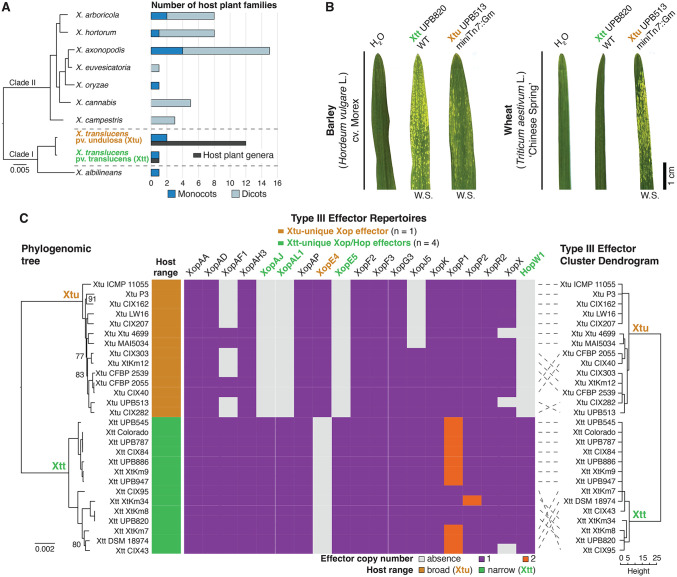
*X. translucens* pathovars with differential host range breadth encode significantly different effector repertoires. (A) Phylogenomics and host range of representative *Xanthomonas* spp. The number of plant families that are known hosts (natural or experimentally determined) for each analyzed *Xanthomonas* spp. are shown in the chart to the right side of the panel. The number of plant genera that are known hosts for *X. translucens* pathovars translucens and undulosa is also included in the chart. (B) Symptom development in barley (*Hordeum vulgare* L. cv. Morex) and wheat (*Triticum aestivum* L. genotype ‘Chinese Spring’) leaves inoculated via spray with Xtu and Xtt strains (OD_600_ 0.5) at six days post inoculation. Water soaking symptoms were visualized using a light box and appear as lighter areas in the leaf due to being translucent. Xtt elicited no response in wheat. Scale bar is shown in figure. W.S. – water soaking. (C) Effector repertoires of *X. translucens* pvs. translucens and undulosa (only non-TAL effectors are presented). A core genome-based tree of 28 *X. translucens* strains is shown to the left side of the panel. Putative Type III secretion system effectors were identified via local Blastx against a database of known *Xanthomonas* effectors. Clustering of strains according to a hierarchical clustering of their encoded repertoires (Type III effector cluster dendrogram) is shown to the right side of the panel. Trees were visualized via FigTree, and mid-point rooted. Branches with bootstrap values below 98% are indicated in each tree. Xtt - *X. translucens* pv. translucens; Xtu - *X. translucens* pv. undulosa.

Host range is a particular type of niche-specificity, and many pathogenic organisms can be found across the spectrum from generalist to specialist. The Gammaproteobacterial genus *Xanthomonas* limits agricultural production by causing diseases in over 400 identified plant species. *Xanthomonas* bacteria in the generalist spectrum cause disease in a broad range of host genera with access to a breadth of potential hosts for resources, while specialists remain restricted to a particular species or genus (Figs 1A and S1A) [6–8]. Differences in niche-specificity of closely related plant pathogenic species and strains are often due to small changes in genome content [9–12] but the evolutionary mechanisms for niche specialization of *Xanthomonas* spp. remain poorly understood. Ultimately, understanding the spectrum and evolution of host range specificity will benefit prediction and mitigation of disease epidemics [5,13].

Bacterial organisms regularly secrete proteins and other molecules into the extracellular environment or directly into host cells to interact with their niche aiming at promoting growth, survival, colonization and virulence [14]. Many Gram-negative animal and plant bacterial pathogens, including *Xanthomonas spp.*, depend on a Type III secretion system (T3SS) for injecting effector proteins into the host cells to aid in colonization. Effectors usually function by altering molecular pathways within the host that lead to susceptibility. However, they may also trigger plant resistance if recognized by components of the host’s immune system [15,16]. Our previous work revealed that frequent loss and gain of a single gene encoding a Type II secreted cellulase effector, CbsA, promoted lifestyle switch for changes in tissue-specific host adaptation [2]. Here, we used a similar framework to define the evolution of host range shift in specialist and generalist subgroups (i.e., respectively, narrow and broad host range) of *Xanthomonas translucens*, arguably the most important re-emergent bacterial pathogen of cereals beyond rice [17–19]. We have determined that evolutionary loss and gain of Type III effectors (T3Es) govern the phenotypic switch between specialist and generalist behaviors of *X. translucens* subgroups for host range by either individually expanding or restricting host specificity. The broad host range lineage lost a single effector, XopAL1, to evade wheat defenses but at a fitness cost on a primary host. On the other hand, the narrow host range lineage gained the effector XopAJ, which restricted its ability to cause disease in oat but increased its virulence in barley. We assessed the potential for contemporary *X. translucens* specialist lineages to emerge via host jump, thus providing a framework for epidemiological tracking of particular lineages of risk.

## Results

### *Xanthomonas translucens* subgroups with differential host range behaviors encode significantly different T3E repertoires

Bacteria in the *X. translucens* species primarily infect grass and cereal plants in the Poaceae family, causing bacterial leaf streak (BLS) and blight, and black chaff of cereals [17,19]. Early documentation from the 1900s described the broad host range of generalist *X. translucens* pv. undulosa (Xtu) [20,21]. This subgroup uses a wide range of metabolites upon infection of host plants [22] and causes disease in wheat, barley, rye, triticale, spelt, wild oat, ornamental asparagus, wild rice, and in a variety of wild grasses, totaling 12 plant genera (Figs 1A and S1A) [19–21,23–27]. In contrast, the closely related *X. translucens* pv. translucens (Xtt) is a specialist with limited metabolism that only causes disease in plants of the *Hordeum* genus (barley; Figs 1A-1B and S1A) [20,22,24]. The main small grains affected by Xtt and Xtu are barley and wheat, respectively [17,24]. For example, upon spray inoculation for naturalistic entry and infection, Xtt and Xtu both caused the water soaking (i.e., leaf lesion that appears wet, dark, and translucent) disease symptom development in barley, but only generalist Xtu caused disease in wheat (Fig 1B).

To better understand differences in niche specificity between the closely related (>97% average nucleotide identity) [28] but phylogenomically distinct Xtt and Xtu (Fig 1A,1C), comparative genomic analyses were conducted using the pangenome analysis tool Panaroo [29]. Results revealed that over 3,000 core proteins are shared between them (S1 Table). On the other hand, each subgroup had over 100 unique conserved proteins (S2 Table). Secreted proteins, which are often associated with virulence [14], represented 3% and 3.5% of shared (core genome) and unique proteins (accessory genome), respectively (S1-S2 Tables). The number of unique, overall secreted proteins were not significantly different between Xtt and Xtu (Student’s *t*-*t*est; *P* value = 0.7772).

Based on the biological importance of T3Es [15,16], we aimed to determine if there is a link between host range of *X. translucens* and encoded T3E repertoires. Patterns of T3E gene presence and absence in complete Xtt and Xtu genomes were determined via blastx using a database of known *Xanthomonas* spp. T3Es, as previously described [30]. Hierarchical clustering of strains according to their encoded effector repertoires (Fig 1C; right side of the panel) was congruent with the subspecies tree and with generalist and specialist behaviors (Fig 1C; left side of the panel). This suggests that encoded T3E repertoires closely relate to host range behaviors of *X. translucens*. Specialist Xtt has a larger T3E repertoire (mean average of 18 effector genes encoded per genome) than generalist Xtu (mean average of 13 effector genes encoded per genome) (Fig 1C; Wilcoxon rank-sum test; *P* value = 3.436 e-06). Xtt genomes encode an expanded effector repertoire that include the unique effectors XopAJ, XopAL1, XopE5, and HopW1 and an effector family expansion for XopP1 and XopP2, in comparison to Xtu. Only one unique T3E was identified in Xtu (XopE4), while seven were overall absent in this generalist subgroup (Fig 1C). Moreover, Type III-secreted transcription activator-like (TAL) effectors were identified via AnnoTALE [31] and were significantly more abundant in broad host range Xtu (S1B Fig; Student’s *t*-test; *P* value = 0.02511). Highly virulent Xtu strains have previously been found to precisely manipulate plant hormone pathways via the Tal8 (synonym TalDC) effector to cause wheat bacterial leaf streak [32]. This effector is not encoded by low-virulence strains of Xtu or any Xtt strain (S1B Fig) [32]. We therefore hypothesize the TAL effector family expansion correlates with adaptation to new niches as these are highly evolvable repetitive elements [33]. While we only focused on non-TAL effectors in this study, future work will explore host adaptation based on this special and abundant class of effectors.

### The T3E XopAL1 affects both host range and fitness of *X. translucens*

**Fig 2:**
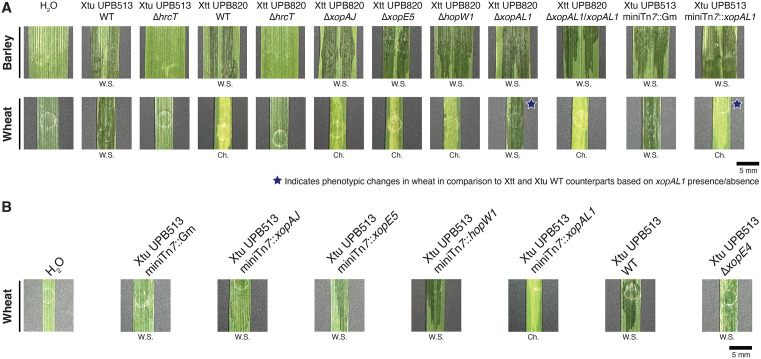
XopAL1 is the only Xtt-unique T3E mediating host jump from barley to wheat. (A) Symptom development in barley and wheat leaves inoculated via syringe infiltration with Xtu and Xtt strains (wild-type, mutant, and insertion strains; OD_600_ 0.1) at three days post inoculation. Scale bar is shown in figure. (B) Symptom development in wheat leaves inoculated via syringe infiltration with Xtu strains (wild-type, mutant, and insertion strains; OD_600_ 0.1) at three days post inoculation. Scale bar is shown in figure. Barley – *Hordeum vulgare* L. cv. Morex; Wheat – *Triticum aestivum* L. genotype ‘Chinese Spring’; Xtt - *X. translucens* pv. translucens; Xtu - *X. translucens* pv. undulosa; Ch. – chlorosis (appear as yellow lesions); W.S. – water soaking (appear as wet, dark lesions due to the dark background).

We hypothesized that presence and absence patterns of individual T3Es might be responsible for host range differences between Xtt and Xtu. We first tested pathogenicity on both barley and wheat for ∆*hrcT* mutants that lack a critical component for the *X. translucens* T3SS by inoculating leaves via syringe infiltration [16]. ∆*hrcT* mutants of Xtt and Xtu did not cause disease symptoms (i.e., water soaking) in barley or wheat (Fig 2A). Unlike the lack of symptom development upon natural infection via spray (Fig 1B), Xtt caused chlorosis (i.e., leaf yellowing at the site of inoculation) in wheat when inoculated via syringe infiltration (Fig 2A). Chlorosis is sometimes associated with disease symptom caused by plant pathogens or abiotic stress factors. However, in this pathosystem, this phenotype has been described as a non-host or non-pathogenic response that prevents disease development [34]. Thus, in our screening process, the development of chlorosis indicates a lack of disease, whereas the appearance of water soaking denotes disease development.

Results with the *∆hrcT* mutants of Xtt and Xtu demonstrated that both pathogenicity (water soaking symptom development) and the chlorotic non-host response are dependent on T3Es. We then aimed to define if a unique Xtt effector prevented wheat pathogenesis by screening for loss of chlorosis. All four Xtt-unique effectors (*xopAJ*, *xopAL1*, *xopE5*, and *hopW1*) were individually deleted via clean deletion [2] and inoculated into barley and wheat leaves via infiltration. We hypothesized that if one of the Xtt-unique effectors was responsible for triggering the chlorotic non-host response in wheat, we would observe a loss of chlorosis following inoculation with the mutant strains. Individual mutant strains of the four Xtt-unique effector genes retained wild-type (WT) pathogenicity, as all still caused water soaking in barley, and all but Xtt ∆*xopAL1* triggered the chlorotic non-host response in wheat (Fig 2A). Notably, *xopAL1* deletion in Xtt produced water soaking disease symptom development in wheat similar to Xtu BLS (Fig 2A). Complementation of the Xtt ∆*xopAL1* mutant strain or *xopAL1* introduction into the true wheat pathogen Xtu via miniTn*7* insertion (Xtu miniTn7::*xopAL1*) triggered development of the non-pathogenic, chlorosis phenotype in wheat (Fig 2A). Introduction of the other three Xtt-unique effectors in Xtu did not trigger the wheat chlorotic response (Fig 2B).

To determine whether wheat pathogenicity of Xtu depends on its unique effector *xopE4*, we evaluated a deletion mutant strain of this gene. Wheat leaves infiltrated with Xtu ∆*xopE4* still displayed water-soaking symptoms (Fig 2B). Paired with our Xtt results in which deletion of the single effector gene *xopAL1* was sufficient to allow pathogenicity on wheat, these data demonstrate that XopE4 is not required for causing disease in this host plant.

**Fig 3:**
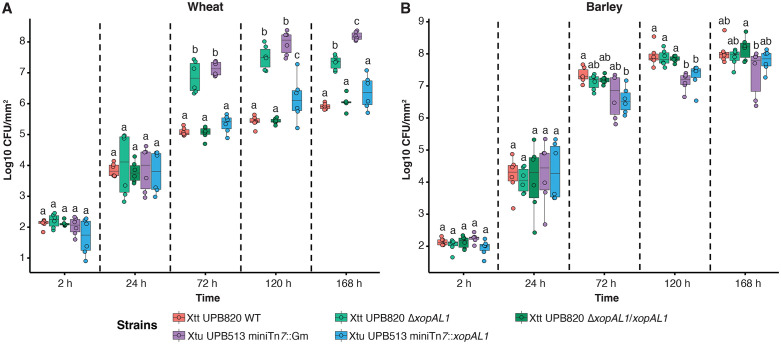
Presence of *xopAL1* significantly reduces the population of *X. translucens* in wheat. Xtt and Xtu (wild-type, mutant, complemented and insertion strains for *xopAL1*) populations in planta determined at 24- to 48-hour intervals in wheat (A) and barley leaves (B). Xtt strains were previously transformed with pPneo-GFP (kanamycin resistance), and Xtu strains were previously transformed via miniTn*7*-Gm (gentamicin resistance) insertion, to allow for growth in selective medium. Leaves were inoculated with Xtt and Xtu strains via syringe infiltration (OD_600_ 0.001). Different letters indicate significant difference per time point (separated by vertical dashed lines) as determined by one way ANOVA and Tukey’s post-hoc test in the log-transformed data (*P* < 0.05; n = two independent replicates with three biological replicates within each time point and inoculated strain, with three internal replicates each). Wheat – *Triticum aestivum* L. genotype ‘Chinese Spring’; Barley – *Hordeum vulgare* L. cv. Morex; Xtt – *X. translucens* pv. translucens; Xtu – *X. translucens* pv. undulosa.

To assess whether the observed symptoms correlated with differences in bacterial colonization, endophytic populations of Xtt and Xtu strains, with or without *xopAL1*, were quantified via isolation and counting of colony forming units (CFU). Xtt ∆*xopAL1* had a similar population to Xtu in wheat upon syringe infiltration, whereas introduction of this effector gene into Xtu (Xtu miniTn7::*xopAL1*) significantly reduced its population to similar levels as Xtt WT (Fig 3A). On the other hand, Xtu presented overall slower growth in barley than Xtt, but significant differences mediated by XopAL1 were generally not observed in either Xtt or Xtu in this host plant (Fig 3B).

**Fig 4:**
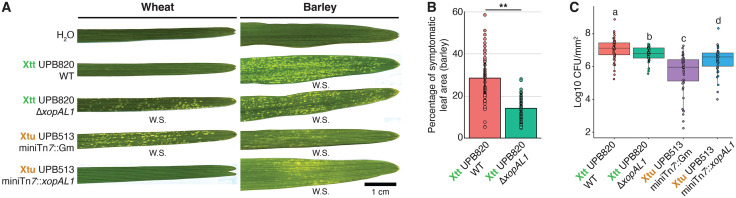
Deletion of the T3E gene *xopAL1* promotes Xtt host jump but at a fitness cost. (A) Symptom development in barley and wheat leaves inoculated via spray with Xtu and Xtt strains (wild-type, mutant, and insertion strains; OD_600_ 0.5) at six days post inoculation. Water soaking symptoms were visualized using a light box and appear as lighter areas in the leaf due to being translucent. Scale bar is shown in figure. (B) Percentage of symptomatic leaf area of inoculated barley leaves via spray with Xtt WT and Xtt ∆*xopAL1* shown in panel A. Original data of percentage of symptomatic leaf area (treatments; y-axis) was submitted to a transformation that combines the arcsine and square root functions. The Shapiro-Wilk test (W) was then applied to the transformed data, and it supported the assumption of normal distribution (W = 0.975; *P* value = 0.0671). ** indicates significant difference as determined by Student’s *t*-test (*P* < 0.001; n = two independent replicates with 23–24 biological replicates each). (C) Xtt and Xtu (wild-type, mutant, and insertion strains) populations in barley leaves inoculated via spray (OD_600_ 0.5) at 144 hours post inoculation. Different letters indicate significant difference as determined by Wilcoxon rank-sum test (*P* < 0.05; n = two independent replicates with 23–24 biological replicates, each with three internal replicates). Wheat – *Triticum aestivum* L. genotype ‘Chinese Spring’; Barley – *Hordeum vulgare* L. cv. Morex; Xtt - *X. translucens* pv. translucens; Xtu - *X. translucens* pv. undulosa; W.S. – water soaking.

These findings were further assessed by spray inoculation, an inoculation method more similar to natural infections than syringe infiltration. Xtt ∆*xopAL1* caused water-soaking BLS symptoms similar to Xtu on wheat and barley, while introduction of this effector into Xtu disrupted its ability to cause disease in wheat (Fig 4A). Deletion of *xopAL1* did not affect the pathogenicity of Xtt (ability to cause disease or not; Fig 4A), but we observed that Xtt ∆*xopAL1* inoculated via spray caused significantly less water soaking symptoms on barley leaves than its WT counterpart (Fig 4A-4B). Deletion of this effector gene significantly reduced the mean average of the symptomatic leaf area of barley from 28% (Xtt WT) to 14% (Xtt ∆*xopAL1*), resulting in a two-fold decrease in symptom development (Fig 4B; Student’s *t*-test; *P* value = 9.003 e-11). We thus concluded that XopAL1 affects virulence (severity of disease) of Xtt. Because of the reduced virulence of Xtt ∆*xopAL1* and Xtu although pathogenic to barley is infrequently isolated from this host plant [35], we suspected that XopAL1 plays a role in bacterial fitness beyond restricting the ability of Xtt to cause disease in wheat. We tested this hypothesis by quantifying the endophytic populations of Xtt and Xtu in barley leaves inoculated via spray by CFU counting. Significantly higher endophytic populations of Xtt (WT) and Xtu miniTn7::*xopAL1* were observed in barley leaves in comparison to non-*xopAL1*-encoding strains (Xtt ∆*xopAL1* and Xtu WT; Fig 4C). Deletion of *xopAL1* decreased the mean average of the Xtt population by 76% (Fig 4C; approximately four-fold; Wilcoxon rank-sum test; *P* value = 6.00 e-03), and introduction of this effector gene into Xtu increased its population mean average by 313% (Fig 4C; approximately four-fold; Wilcoxon rank-sum test; *P* value = 1.00 e-03). Since these differences were not observed when strains were inoculated via infiltration (Fig 3B), we hypothesize that XopAL1 plays a role in the early stages of barley infection. Altogether, results demonstrated that *xopAL1* is a fitness factor for Xtt virulence and colonization in specialized host barley, but it restricts its ability to cause disease in wheat in an effector-dependent manner possibly via XopAL1 recognition by components of the wheat immune system.

A cereal genotype diversity panel containing known susceptible hosts to Xtu was screened via leaf infiltration to assess if other plant species display a similar *xopAL1*-dependent non-host response mechanism (S3 Table). All screened hexaploid wheat genotypes (*Triticum aestivum* L., BBAADD genotype) presented a *xopAL1*-dependent chlorotic non-host response against Xtt. Diploid (einkorn, AA genotype) and tetraploid (durum and emmer, BBAA genotypes) wheat genotypes were overall more susceptible to Xtt in a XopAL1-independent manner. All tested Triticale lines (hexaploid genotype BBAARR) displayed a *xopAL1*-dependent non-host response against Xtt. Despite Triticale being a wheat-rye hybrid [36], no *xopAL1*-dependent non-host response was observed in rye, which remained resistant to Xtt. Genomic introduction of *xopAL1* into Xtu did not disrupt its pathogenicity in Triticale, as opposed to wheat.

### XopAL1 mediates global transcriptional reprogramming in wheat leading to a chlorotic non-host response

The basis of chlorosis development in a non-host response is widespread in *Xanthomonas*-plant interactions, but its mechanism remains unclear [8,34,37]. For instance, a distant XopAL1 ortholog encoded by *Xanthomonas campestris* pv. campestris (S2 Fig) also triggered chlorosis development in wheat when transformed in Xtu (S3 Fig). We have already established in this study that chlorosis is an effector-triggered response of wheat against Xtt (Fig 2). However, this non-host response differs from a canonical effector-triggered immunity (i.e., hypersensitive response) because there is no programmed cell death at the site of infection leading to necrosis, and because it reduces growth but does not kill incompatible pathogens, such as Xtt, in a short term (Fig 3A) [16,38]. Currently, Xtt-caused chlorosis in wheat is considered as an undefined plant non-host response that prevents disease development by Xtt [34].

**Fig 5:**
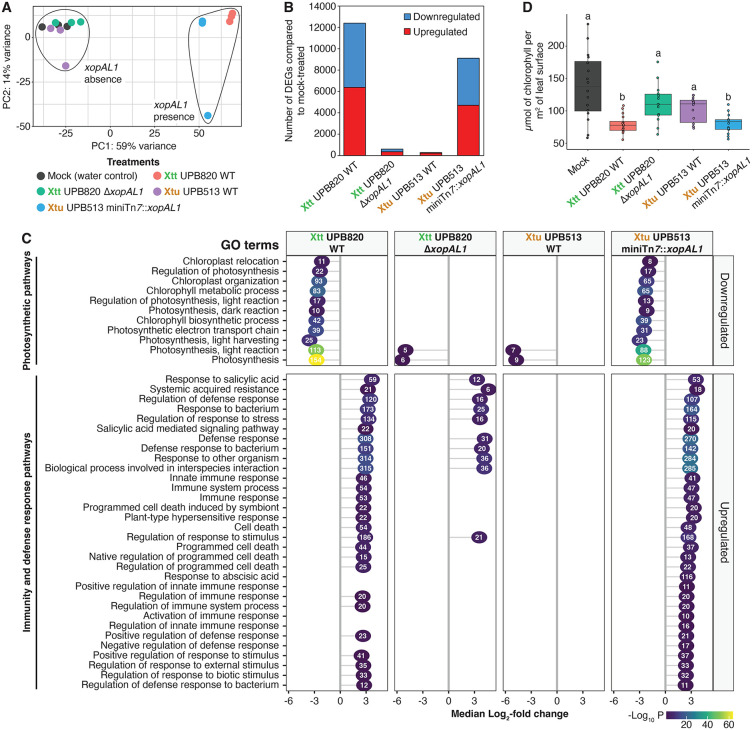
XopAL1 mediates global transcriptional changes in wheat plants, leading to a chlorotic non-host response. (A) Principal component analysis (PCA) of the normalized RNA-seq data transcripts per million of wheat leaf tissue infected with different *X. translucens* strains and mock (treatments). Ellipses are arbitrarily drawn to indicate clustering of treatment based on *xopAL1* presence and absence. (B) Number of significantly differentially expressed genes (DEGs; downregulated and upregulated) in wheat from each bacterial treatment compared to mock. (C) A subset of Gene Ontology (GO) terms significantly enriched in wheat from bacterial treatments versus mock comparisons. The number of genes that fall into a GO term from each comparison are indicated within each dot. *P* values are indicated by dot color (color scale is shown in figure). Upregulated and downregulated GO terms are indicated in the plot. (D) Chlorophyll concentration measurement in wheat leaves (*Triticum aestivum* L. genotype ‘Chinese Spring’) inoculated with Xtt and Xtu strains (wild-type, mutant, and insertion strains) via leaf syringe infiltration (OD_600_ 0.1) at three days post inoculation. Different letters indicate significant difference as determined by Wilcoxon rank-sum test (*P* < 0.05; n = two independent replicates with 6–9 biological replicates, each with three internal replicates). Xtt - *X. translucens* pv. translucens; Xtu - *X. translucens* pv. undulosa.

The molecular and physiological changes of plants during a non-host response are largely undescribed. To address this knowledge gap, we carried out RNA sequencing (RNA-seq) of inoculated wheat leaves to further elucidate the mechanistic basis of non-host response that restricts bacterial infection. Expression profiles were compared between wheat leaves inoculated with *xopAL1*-encoding strains (Xtt WT and Xtu miniTn7::*xopAL1*), non-encoding strains (Xtt ∆*xopAL1* and Xtu WT), and mock-inoculated leaves. A principal component analysis (PCA) of the RNA-seq count data demonstrated that samples clustered in two primary transcriptional profiles in a *xopAL1*-dependent manner, reflecting the ability of strains to cause disease in wheat or not (Fig 5A). Accordingly, much higher numbers of significantly differentially expressed genes (DEGs) were identified in plants inoculated with Xtt and Xtu encoding *xopAL1* when compared to mock-treated samples (12,397 and 9,118 DEGs, respectively; S4 Table) and in comparison to non-encoding strains of these subgroups (607 and 285 DEGs, respectively; S4 Table) (Fig 5B). Leaves inoculated with non-encoding, pathogenic strains clustered with mock-treated leaves (Fig 5A), demonstrating that XopAL1 is the main factor driving transcriptional changes in wheat.

A gene ontology (GO) term enrichment analysis was performed to investigate the specific plant processes in wheat that led to the non-host response. Our analysis revealed that upregulated DEGs in response to inoculation with Xtt and Xtu expressing *xopAL1* were significantly enriched in GO terms associated with immunity, induced defense response, defense signaling, and programmed cell death (Fig 5C; S4 Table). On the other hand, significant overrepresentation of GO terms associated with photosynthetic pathways, including chloroplast and chlorophyll processes, were observed among the downregulated DEGs in response to strains expressing *xopAL1* (Fig 5C; S4 Table). Based on this, we hypothesized that chlorosis is the result of a defense response that leads to reduced photosynthetic activity and chlorophyll content in wheat upon XopAL1 recognition. Indeed, we observed significantly lower chlorophyll concentration in wheat leaves inoculated with *xopAL1*-encoding Xtt and Xtu in comparison to those inoculated with non-encoding strains and mock-inoculated leaves (Fig 5D). Wheat leaves inoculated with Xtt ∆*xopAL1* had a mean average of chlorophyll concentration 40% higher than leaves inoculated with Xtt WT (Wilcoxon rank-sum test; *P* value = 0.011). Likewise, leaves inoculated with Xtu miniTn7::*xopAL1* presented a 21% decrease in the mean average of their chlorophyll concentration in comparison to inoculation with their WT counterpart (Wilcoxon rank-sum test; *P* value = 0.031). These findings indicate that recognition of XopAL1 in wheat results in extensive transcriptional changes that ultimately lead to a non-host type of effector-triggered resistance response.

### *xopAL1* has been lost in Xtu and presents an inherent risk for host jump by Xtt

**Fig 6:**
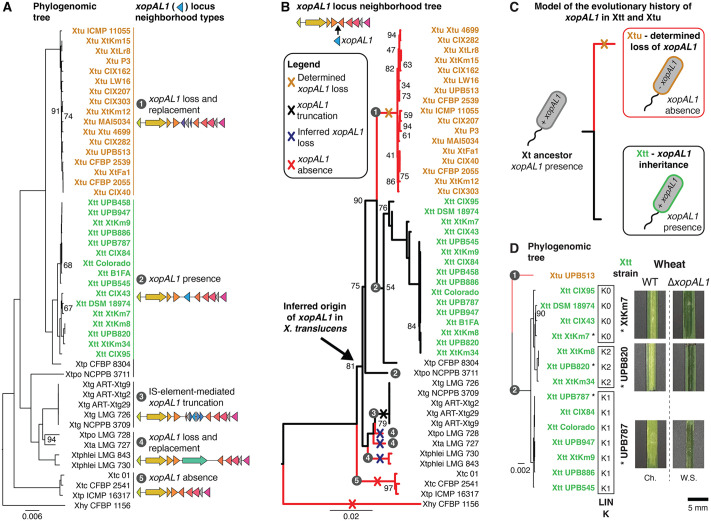
Loss of *xopAL1* promoted host jump of *X. translucens* pv. undulosa to wheat. (A) Core genome-based tree of *X. translucens* strains and their *xopAL1* locus neighborhood types. A complete list of genes composing each *xopAL1*-neighborhood is shown in S4 Fig. (B) Tree of the concatenated sequences of the genes flanking *xopAL1* (4 upstream and 5 downstream genes) in *X. translucens*. The *xopAL1* locus neighborhood types are indicated in each branch. Red-colored branches indicate subgroups that do not encode *xopAL1*, with determined and inferred loss or absence being shown. *X. hyacinthi* strain CFBP 1156 was included as an outgroup to root the trees in panels A and B. (C) Model of the evolutionary history of *xopAL1* in Xtt and Xtu. (D) Deletion of *xopAL1* promotes host jump of all Xtt genomic subgroups. A core genome-based tree including the K0, K1 and K2 genomic subgroups of Xtt is shown. Symptom development in wheat (*Triticum aestivum* L. genotype ‘Chinese Spring’) leaves inoculated via syringe infiltration with representative Xtt strains (OD_600_ = 0.1) belonging to the three K genomic subgroups and their respective *xopAL1* mutants at three days post inoculation is shown to the right side of the panel. Scale bar is shown in figure. Trees were visualized via Figtree. Branches with bootstrap values below 98% are indicated in each tree. Ch. – chlorosis (appear as yellow lesions); W.S. – water soaking (appear as wet, dark lesions due to the dark background). *X. translucens* pathovars: Xtc – pv. cerealis; Xtp – pv. pistaciae; Xtg – pv. graminis; Xtpo – pv. poae; Xta – pv. arrhenateri; Xtphlei – pv. phleipratensis; Xtu – pv. undulosa; Xtt – pv. translucens; Xhy – *X. hyacinthi*.

Plant pathogen populations may shift and diversify their encoded virulence factor repertoires to avoid recognition by the host immune system [1,39]. We therefore tested if the emergence of Xtu in wheat correlated with the loss of *xopAL1*. Xtu was suggested to have diverged from a Xtt ancestor [40]. The evolution of *xopAL1* was examined with maximum likelihood phylogenetic comparisons of whole genomes alongside the immediate genomic neighborhood of this effector gene (four upstream and five downstream genes of *xopAL1*; Fig 6A-6B). We evaluated syntenic gene arrangements across subgroups of *X. translucens* (Fig 6B) and reconciled the topologies of obtained trees with the *xopAL1* gene tree (S2A Fig). To define and compare these regions across genomes of *X. translucens*, we used Clinker to visualize the alignment, orientation, and synteny of gene clusters flanking the *xopAL1* locus (i.e., genomic neighborhoods; S4 Fig).

Specific neighborhoods of *xopAL1* were defined across the *X. translucens* species (Figs 6A and S4). Our phylogenetic hypothesis testing of the *X. translucens xopAL1* locus determined that this effector was lost in Xtu (*xopAL1*-neighborhood type 1) during its divergence from a common ancestor with Xtt (*xopAL1*-neighborhood type 2; Fig 6B-6C). Loss of *xopAL1* in Xtu was accompanied by the replacement of this effector gene by three other genes across its entire clade (S4 Fig). These include *gvpU* (encoding a gas vesicle accessory protein), a gene encoding a hypothetical protein, and a gene encoding a low molecular weight protein tyrosine phosphatase (S4 Fig). While we did not assess whether these acquired genes affect the host range of Xtu, and their evolutionary origin remains elusive, this finding demonstrates that the evolution of this genomic locus is dynamic, underscoring a complex genomic rearrangement rather than a simple deletion event.

Loss of *xopAL1* was common across other *X. translucens* lineages. Three other inferred losses of *xopAL1* with acquisition of different genes replacing this effector were observed in subgroups with *xopAL1*-neighborhood type 4 (Fig 6B). The origin of *xopAL1* (*xopAL1*-neighborhood type 2) was parsimoniously inferred in *X. translucens* to occur after divergence from a common ancestor with pathovar cerealis and strain ICMP 16317 in pathovar pistaciae (Xtp; *xopAL1*-neighborhood type 5; Fig 6B). In addition, there was truncation of *xopAL1* in pathovar graminis (Xtg) by direct transposon insertion 5’ of the remaining sequence fragment (*xopAL1*-neighborhood type 3; Figs 6B and S2B). For comparison, we analyzed the evolution of *xopE5* and *hopW1* (other Xtt-unique effector genes) using the same framework and also observed loss in Xtu for both (S5-S6 Figs, respectively).

Risk assessment is key for epidemic preparedness, but there is often a lack of precise knowledge about what genetic events lead to emergence, and about the potential of organisms to change their niche. Because loss of *xopAL1* allowed Xtu to jump host to wheat, we examined if deletion of this effector gene could lead to host jump for the three described, globally-distributed Xtt genomic subgroups (K0, K1, and K2) [30,35]. In comparison to our K2 model strain used in this study (strain UPB820), K1 strains shared 100% homology of XopAL1 (strain UPB787) but K0 strains shared between one (strain XtKm7) to two (strain CIX95) amino acid changes in their XopAL1 sequences (S2 Fig). Nonetheless, the deletion of *xopAL1* in representative K0, K1 and K2 strains led to host expansion of all to wheat (Fig 6D). Xtt lineages are globally important in barley production systems [30], and all have an inherent pathogenic capacity in wheat production systems based on *xopAL1* loss.

### The T3E XopAJ is another host range determinant of *X. translucens*

**Fig 7:**
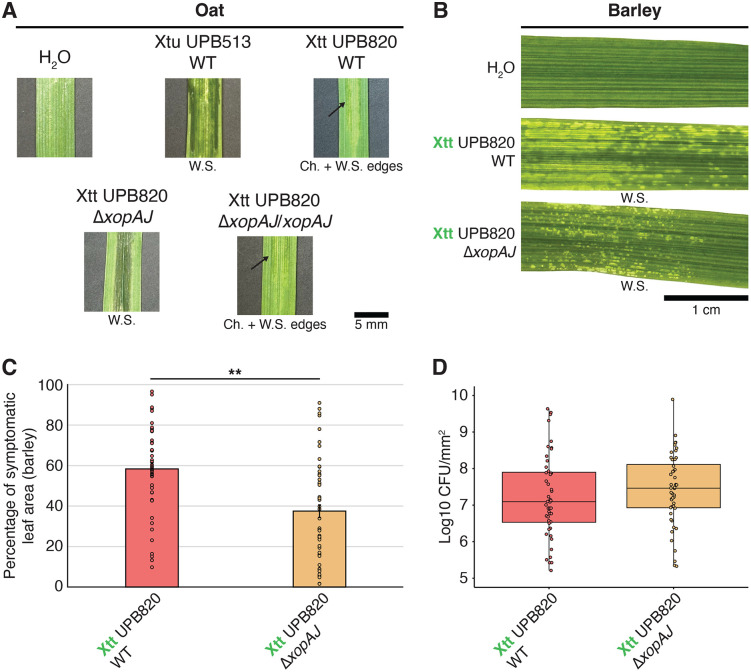
Deletion of the T3E gene *xopAJ* allows for disease development caused by Xtt in oat but decreases virulence in barley. (A) Symptom development in oat (*Avena sativa* L. variety Antigo) leaves inoculated via syringe infiltration with Xtu and Xtt strains (wild-type, mutant, and complemented strains; OD_600_ 0.1) at three days post inoculation. Arrows are pointing to water-soaked edges of chlorotic lesions. (B) Symptom development in barley leaves inoculated via spray with Xtt WT and Xtt ∆*xopAJ* (OD_600_ 0.5) at four days post inoculation. Water soaking symptoms were visualized using a light box and appear as lighter areas in the leaf due to being translucent. (C) Percentage of symptomatic leaf area of inoculated barley leaves via spray with Xtt WT and Xtt ∆*xopAJ* shown in panel B. Original data of percentage of symptomatic leaf area (treatments; y-axis) was submitted to a transformation that combines the arcsine and square root functions. The Shapiro-Wilk test (W) was then applied to the transformed data, and it supported the assumption of normal distribution (W = 0.982; *P* value = 0.223). ** indicates significant difference as determined by Student’s *t*-test (*P* < 0.001; n = two independent replicates with 24 biological replicates each). (D) Xtt WT and Xtt ∆*xopAJ* populations in barley leaves inoculated via spray (OD_600_ 0.5) at four days post inoculation. No significant differences between endophytic populations of Xtt WT and Xtt ∆*xopAJ* were observed according to one-way ANOVA and Tukey’s post-hoc test (*P* > 0.05; n = two independent replicates with 24 biological replicates each). Scale bars are shown in figure. Barley – *Hordeum vulgare* L. cv. Morex; Xtu - *X. translucens* pv. undulosa; Xtt - *X. translucens* pv. translucens; Ch. – chlorosis (appear as yellow lesions); W.S. – water soaking (appear as wet, dark lesions due to the dark background).

Barley and wheat are the main crops affected by *X. translucens* [17,24]. However, the host range of this pathogenic species, especially of Xtu, extends to other hosts of economic interest, such as rye, triticale, and oat [20,21,23,24]. To determine if the other Xtt-unique effectors beyond XopAL1 (XopAJ, XopE5, and HopW1) modulate the host range of this subgroup, we gathered a subset of the cereal genotype diversity panel that was inoculated with the *xopAL1* mutant (S3 Table) and screened it with individual mutant strains of *xopAJ*, *xopE5*, and *hopW1* (S5 Table). Leaves of each host were inoculated via infiltration and we analyzed changes in host range by observing whether the Xtt mutants lost the ability to cause the chlorotic non-host response. No changes were observed except for *xopAJ* (S5 Table). Xtt WT caused chlorosis development with small water-soaked edges on oat (*Avena sativa* L.) leaves, while Xtt ∆*xopAJ* caused strong water soaking development similar to Xtu BLS (Fig 7A). Complementation of *xopAJ* restored the WT phenotype (Fig 7A). This *xopAJ*-dependent resistance response of oat against Xtt demonstrates that this effector restricts the host range of this specialist subgroup by not allowing it to cause disease in oat.

### *xopAJ* has been gained by Xtt and enhances virulence in barley

**Fig 8:**
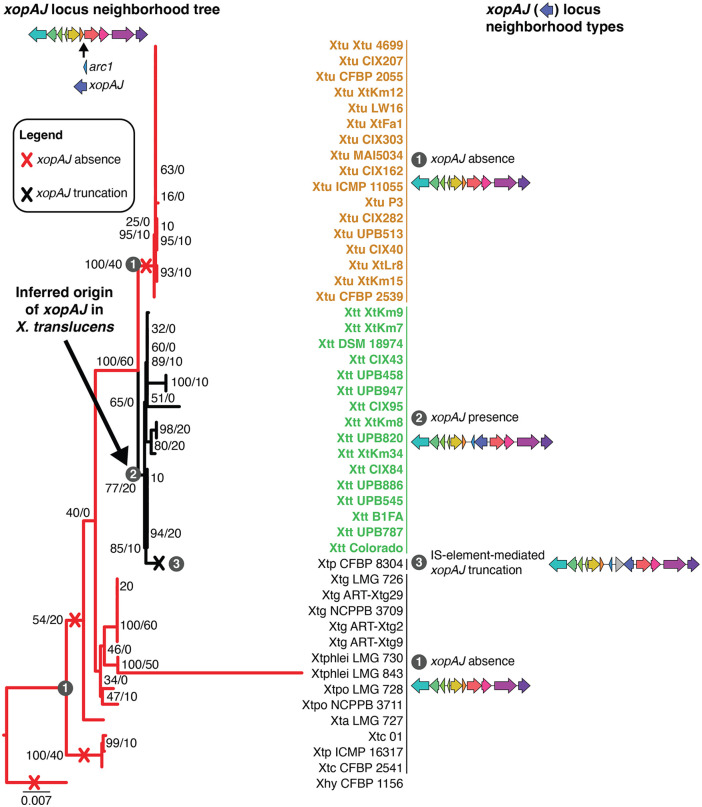
*xopAJ* was gained by *X. translucens* pv. translucens. Tree of the concatenated amino acid sequences of the genes flanking *xopAJ* (6 upstream and 4 downstream genes) in *X. translucens*. The *xopAJ* locus neighborhood types are highlighted within each branch and shown to the right side of the figure (see S7A Fig for more details). Red-colored branches indicate subgroups that do not encode this effector, with absence being shown (see tree legend). *X. hyacinthi* strain CFBP 1156, a closely-related clade I *Xanthomonas* spp., was included as an outgroup to root the tree. The tree was visualized via Figtree. Branches are labeled with bootstrap values followed by the gene concordance factor (gCF). Xtu – *X. translucens* pv. undulosa; Xtt – *X. translucens* pv. translucens; Xtp – *X. translucens* pv. pistaciae; Xtc – *X. translucens* pv. cerealis; Xtg – *X. translucens* pv. graminis; Xtphlei – *X. translucens* pv. phleipratensis; Xtpo – *X. translucens* pv. poae; Xta – *X. translucens* pv. arrhenateri; Xhy – *X. hyacinthi*.

Since we determined that XopAJ also affects the host range of *X. translucens*, we used the same evolutionary analysis framework as the one described for *xopAL1* to assess if this gene was lost or gained between Xtt and Xtu. Gene gain is another mechanism for niche shift in *Xanthomonas* [2], and our results determined that the oat-limiting effector gene *xopAJ* originated in Xtt (*xopAJ*-neighborhood 2) after divergence with Xtu (*xopAJ*-neighborhood 1; Figs 8 and S7A). This gene was also gained in related pistachio-infecting *X. translucens* (Xtp) strain CFBP 8304 but further truncated via transposon insertion (*xopAJ*-neighborhood 3; Figs 8 and S7A). *xopAJ* is not encoded by any other *X. translucens* subgroup and is not encoded by Xtt in the same locus as other *xopAJ*-encoding *Xanthomonas* spp. (S7B Fig). Moreover, Xtt-encoded *xopAJ* is more phylogenetically closely related to *xopAJ* encoded by a distant species, *Paracidovorax citrulli*, than to *xopAJ* encoded by other *Xanthomonas* spp. (S8 Fig). These results collectively suggest that *xopAJ* evolution is dynamic and was gained in Xtt by horizontal gene transfer but lost by dicot pathogen Xtp strain CFBP 8304.

We hypothesized that evolutionary gain and maintenance of *xopAJ* by Xtt increased its adaptive fitness to colonize barley and tested this by performing spray inoculation of barley leaves with Xtt WT and ∆*xopAJ*. Xtt ∆*xopAJ* was still pathogenic to barley, but the symptomatic leaf area of barley decreased significantly with the deletion of this effector gene (Fig 7B-7C). Deletion of *xopAJ* significantly reduced the mean average of the symptomatic leaf area of barley from 58% (Xtt WT) to 37% (Xtt ∆*xopAJ*), resulting in a 35% decrease in symptom development (Fig 7B; Student’s *t*-test; *P* value = 4.126 e-05). On the other hand, counting of CFU revealed that no significant changes occurred in endophytic populations of the pathogen upon *xopAJ* deletion (Fig 7D). Altogether, results show that gain of *xopAJ* enhanced virulence of Xtt in barley, while not affecting fitness for plant colonization.

## Discussion

Emerging infectious diseases often result from microbial niche shift. Multiple genetic mechanisms control emergence including gene loss and gain. Genome and episome reduction are strongly associated with host specialization and gain can promote adaptation to a particular environment or resist control interventions (i.e., antibiotics) [41,42]. Here, we have determined that loss and gain of single T3Es modulate specialist and generalist behaviors of Xtt and Xtu for host range. Specifically, our results collectively demonstrate that a) XopAL1 is both a fitness and virulence requirement for Xtt; b) this effector is a determinant of host range since its loss contributed for host expansion of Xtu to wheat by circumventing an otherwise effector-triggered defense response of this host plant; and c) gain of XopAJ by Xtt limited its ability to cause disease in oat but contributes for full virulence in barley.

Bacteria commonly cause disease in host plants by delivering secreted factors that alter plant cellular processes to promote virulence, but these same factors can also trigger plant immune responses that restrict infection if recognized by cognate plant resistance (R) proteins [15,16]. Absence or presence of specific T3Es dictates host range in an effector-triggered immunity (ETI) -dependent manner. Examples of effectors affecting host range include the XopAG, XopQ, and AvrBsT effectors of *Xanthomonas* spp.[9,10,12,37], and HopQ1-1 effector of *Pseudomonas syringae* [11]. Moreover, effectors have demonstrated fitness requirements for leaf colonization in particular hosts [39,43]. For instance, absence of AvrBsT expands the host range of *Xanthomonas perforans* while decreasing its fitness in tomato fields [43].

These studies demonstrate how T3E repertoires may vary in response to resistance within individual cropping or model plant systems but lack an evolutionary framework. Here, we determined the phylogenetically-supported evolutionary history and ecological function of T3E loss and gain events related to fitness and host range. Using the generalist model Xtu, we posit that *xopAL1* was one of multiple loss events that led to emergence of this generalist subgroup. Xtu has lost other niche-specific factors for tissue-specificity. Our previous work demonstrated that loss of the vascular-associated gene *cbsA* is frequently associated with non-vascular tissue colonization. This gene is encoded by the vascular, specialist Xtt but was lost in non-vascular, generalist Xtu via transposon insertion [2]. We have also observed truncation of *xopAL1* in Xtg, and *xopAJ* in Xtp strain CFBP 8304 via transposon insertion. Transposons shape microbial niche-specificity, and these insertion sequence (IS) elements are major drivers of evolutionary ecological shifts of bacterial and fungal species [2,44–46]. Overall, we hypothesize that gene loss, frequently mediated by IS elements, is a common strategy for niche shifts across behaviors.

Interestingly, XopAJ, which we have determined here as a host range-limiting factor for *X. translucens* in oat, was gained in Xtt and contributes to virulence in barley. We speculate that gain of effectors upon host specialization provides resource access on a preferred host. XopAJ (synonym of AvrRxo1) was initially described as a host range-limiting factor of the rice pathogen *X. oryzae* pv. oryzicola (Xoc) when inoculated into maize lines carrying the cognate resistance gene Rxo1 [47]. This effector has been shown to suppress pattern-triggered immunity (PTI) responses of host and non-host plants [48], and to enhance virulence of Xoc [49]. Xtt is limited to barley (*Hordeum* spp.), and it remains to be determined whether XopAJ displays similar molecular functional roles for host colonization and virulence as observed in other *Xanthomonas* spp.

Unlike XopAJ, there is no previous functional or mechanistic characterization of XopAL1. Thus, it remains to be determined how this effector interacts with components of the host plant (i.e., barley) to contribute to disease development and pathogen growth within leaves. Comparison of symptom development and endophytic growth in barley leaves inoculated via infiltration and spray demonstrated that XopAL1 is likely important during the early stages of infection. This is because XopAL1 affected the pathogenic fitness only when strains were inoculated via spray and thus have to access the inner leaf tissue by themselves before growing and causing disease. This is similar to what was described for the effector XopAP in Xoc. This effector is critical in the early stages of rice infection by preventing stomatal closure and allowing the pathogen cells to enter leaves [50]. As mentioned, the mechanism by which XopAL1 aids Xtt in this process remains elusive.

Hosts respond dramatically different to specialized and generalist pathogens and could be used to inform resistance trait discovery. Plant R proteins are usually cytoplasmic nucleotide-binding leucine rich-repeat receptors (NLRs) that trigger defense signaling pathways leading to immunity and suppression of a pathogen once one of its effectors or another avirulence factor is recognized [38,51]. However, pathogens may also be recognized by transmembrane pattern recognition receptors (PRRs) [51]. This is the case of the rice Xa21 PRR that recognizes the RaxX peptide produced by the pathogen *X. oryzae* pv. oryzae and triggers an immune response [52,53]. XopAL1 recognition is possibly cytoplasmic due to it being a T3E, and we have not yet identified whether a NLR or PRR recognizes this effector. However, we believe that the DEGs identified in our RNA-seq analysis, especially those upregulated by XopAL1, compose a valuable pool of candidates to identify the interactor(s) of this effector in wheat and generate resistance in other hosts including barley. This will be the focus of a future study. Moreover, our RNA-seq analysis demonstrated downregulation of chloroplast processes by XopAL1, which play a central role in plant immunity [54]. Our RNA-seq analysis help to understand the basis of the chlorotic non-host response that includes upregulation of defense responses with downregulation of photosynthetic processes. Together, results demonstrate that this is an active immune response of the plant, providing valuable insights into the molecular pathways governing non-host resistance.

In addition to identifying the specific pathways leading to disease resistance, the identification of the specific donor species or accession lines from which a resistance gene originated is a critical step to determine candidates to be used in breeding programs. Common wheat (*Triticum aestivum* L.) has a complex allohexaploid genome (2n = 6x = 42; BBAADD) originated from the hybridization of the allotetraploid *T. turgidum* (BBAA genome) wheat with *Aegilops tauschii*, the donor of the DD sub-genome. *T. urartu* is the donor of the AA sub-genome, while the BB sub-genome originated from an unknown species likely related to *Ae. speltoides* [55]. Results of our screening of the cereal genotype diversity panel demonstrated that allotetraploid and diploid wheat, as well as the wheat-rye hybrid triticale (BBAARR, where R is the sub-genome inherited from rye) [56], were overall more susceptible to Xtt independent of XopAL1. We therefore hypothesize that wheat resistance against Xtt may be mostly encoded by the DD sub-genome, which has the annual grass *Ae. tauschii* Coss. as the donor species [55].

**Fig 9:**
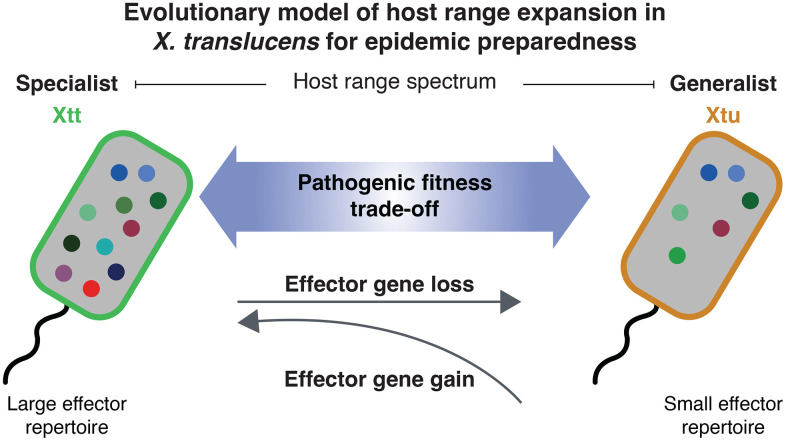
Proposed evolutionary model of *Xanthomonas translucens* host range based on effector gene loss and gain. Taken together, phenotypic and phylogenetic analyses support a model where loss of a single Type III secretion system effector gene, *xopAL1*, allowed for host range expansion to wheat by the generalist Xtu, while gain of *xopAJ* in the specialist Xtt restricted even more its host range breadth. Our model also implies that Xtt strains have an inherent risk for host jump via individual losses of *xopAL1* and *xopAJ*, which has profound effects for epidemic preparedness and management practices. Circles with different colors in figure represent different effector repertoires encoded by Xtt and Xtu. Xtt - *X. translucens* pv. translucens; Xtu - *X. translucens* pv. undulosa.

Preparedness for mitigation of new epidemics, especially when host jump occurs, depends on strong knowledge of the evolution of niche shift. The research described here provides a framework for reconstructing the evolutionary history of host jump in an important cereal pathogen and allows us to predict disease emergence based on gene loss and gain. Based on our results, we developed a tested evolutionary model for *X. translucens* intergenera host jump centered on effector gene loss and gain (Fig 9). Xtu is a generalist that has fewer T3Es, and loss of *xopAL1* contributed to its broad host range behavior by promoting host expansion to wheat. Conversely, gain of *xopAJ* in the specialist Xtt restricted its ability to cause disease in oat. A practical implication of our model is that management of BLS of wheat and oat must include surveillance of all lineages of Xtt in addition to Xtu due to their inherent risk for jumping hosts based on loss of single genes. These lineages operate in close ecological proximity, since wheat and barley are closely related cereal crops that share extensive geographic distributions and frequently coexist in co-cultivation or regional crop rotations [57,58]. Despite the inherent risk of a host jump by Xtt, the loss of *xopAL1* occurred only once during the divergence of Xtu, suggesting that this evolutionary event is ancestral within this lineage and resulted in a significant founder effect. We speculate that once Xtu successfully adapted to the vacant wheat niche, stabilizing selection within the respective host populations likely maintained the distinct host ranges of modern Xtt and Xtu lineages. Ultimately, this work demonstrated that the shift between specialist and generalist subgroups of *X. translucens* for causing disease in individual host plants is modulated by presence and absence of single genes, with gene loss and gain being major drivers for changing niches and ecological behaviors of these microbes.

## Materials and methods

### Bacterial strains, plasmids, and culture conditions

Bacterial strains and plasmids used in this study are listed in S6 Table. *Xanthomonas* strains were recovered from -80ºC glycerol stocks and grown for two days at 28ºC on nutrient agar (NA) or nutrient broth (NB) (3 g/L beef extract, 5 g/L peptone, 15 g/L agar). When needed, the antibiotics kanamycin (Km), gentamicin (Gm), or cycloheximide (CHX) were used at concentrations of 50 µg/ml, 5 µg/ml, and 50 µg/ml, respectively. Sterile deionized water was used for suspending cells in liquid. *Escherichia coli* strains were cultured in Luria-Bertani (LB) medium at 37ºC. LB was supplemented with kanamycin (50 µg/ml), gentamicin (10 µg/ml), or ampicillin (100 µg/ml) whenever necessary. *E. coli* WM3064, a diaminopimelic acid (DAP) auxotrophic strain used for conjugation transformation, was grown as described above, but with LB medium supplemented with DAP (200 µM).

### Recombinant DNA techniques

Genomic DNA of respective strains was isolated using the Monarch Genomic DNA Purification kit (New England Biolabs). Polymerase chain reactions (PCR) were performed using Q5 High-Fidelity DNA Polymerase (New England Biolabs) for cloning sequences into plasmid vectors of interest or using Quick-Load *Taq* 2X Master Mix (New England Biolabs) for other processes. PCR reactions were carried out using standard manufacturer protocols in a ProFlex PCR System thermal cycler (Applied Biosystems). PCR products and agarose gel fragments were purified using the QIAquick PCR and Gel Cleanup kit (QIAGEN). Competent cells of *E. coli* strains were made and transformed using the Mix & Go! *E. coli* Transformation Buffer set (Zymo Research). Plasmid DNA was isolated from overnight cultures of *E. coli* using the QIAprep Spin Miniprep kit (QIAGEN).

Transformation of Xtt UPB820 and Xtu UPB513 with respective plasmids (S6 Table) was performed by electroporation (1.8 kV, 200 Ω, 25 μF and a time constant lower than 5 ms). Some Xtt UPB820 strains were transformed via electroporation with pPneo-GFP for kanamycin resistance and GFP expression (S6 Table). Transformation of Xtt UPB787 and XtKm7 was made via conjugation using the *E. coli* donor strain WM3064. In summary, Xtt strains were grown overnight in 40 mL of NB (28 ºC, 220 rpm), and *E. coli* WM3064 strains were grown overnight in 20 mL of LB + DAP + respective antibiotic (37 ºC, 220 rpm). After, Xtt and *E. coli* cells were collected by centrifugation (4,000 rpm for 10 minutes), suspended into the same volume of media (40 mL of NB for Xtt and 20 mL of LB + DAP for *E. coli* WM3064), centrifuged again and suspended with adequate volume of media to obtain an optical density at 600nm (OD_600_) of 3.0. Then, Xtt and *E. coli* cells were combined at a 1:1 ratio and 200 µL aliquots were spotted on top of MF-Millipore MCE membranes (25 mm diameter, 0.45 µm pore size; Merck Millipore Ltd.) overlaid on NA + DAP. Eight membranes were spotted per transformation event. Plates were incubated for 24 hours at 28ºC, and membranes were then recovered by using sterile forceps and placed into conical tubes containing 20 mL of NB. Cells were removed from membranes by vortexing on maximum for 1 minute, and membranes were removed using sterile forceps. At last, cells were collected by centrifugation (4,000 rpm for 10 minutes), suspended in 1 mL of NB, diluted by 10-fold serial dilution until 10^-2^ and 500 µL aliquots of the 10^-1^ and 10^-2^ suspensions were then plated on NA + antibiotic selective medium (150 mm diameter plates) for growth of transformed cells.

Clean deletion of genes of interest (GOI) in strains of *X. translucens* (S7 Table) was made via *sacB* counter-selection using the pK18mobsacB plasmid, as previously described [2]. Clean deletion of each GOI was confirmed via PCR (S8 Table). Complementation of *xopAL1* and *xopAJ* in the respective mutant strains of each in Xtt UPB820 was also performed via the pK18mobsacB plasmid. The upstream and downstream regions of each gene, together with their open reading frames, were amplified altogether using the upstream region forward primer and the downstream region reverse primer (S8 Table) and then cloned into pK18mobsacB at the HindIII restriction site via Gibson Assembly (New England Biolabs). Insertion of *xopAL1* and *xopAJ* back into their original loci was confirmed via PCR (S8 Table).

To obtain the constructs for genomic insertion via miniTn*7*, the open reading frames of each GOI containing promoter and terminator regions were amplified by PCR and then fused (when needed) and cloned into pUC18miniTn*7*T-Gm at the HindIII restriction site via Gibson Assembly (New England Biolabs). The obtained constructs were confirmed via PCR (S8 Table) and transformed into Xtu UPB513 together with pTNS3 (S6 Table) to promote transposition and single gene insertion. Transformant cells were selected by plating on NA + Gm and confirmed for GOI genomic insertion by PCR (S8 Table).

The *xopAL1*_Xcc8004_ gene (400-bp promoter plus 100-bp terminator, amplicons LM169–170 and LM171-LM172; S8 Table) was cloned from *X. campestris* pv. campestris (Xcc) strain 8004 by GoldenGate strategy using *Bsa*I-mediated restriction-ligation into pEL667, a GoldenGate-compatible derivative of pBBR1MCS2. Ligation products were introduced into *E. coli* by electroporation and conjugated into Xcc using pRK2073 as a helper plasmid in triparental matings.

### Plant growth conditions and inoculation methods

Barley (*Hordeum vulgare* L. cv. Morex) and wheat (*Triticum aestivum* L. varieties ‘Chinese Spring’, RB07, Glenn, and AGS2055) seeds were germinated and grown in growth chambers with cycles of 16 hours of light per day at 22º to 24 ºC and 70% relative humidity for leaf syringe infiltration inoculations, or in greenhouse conditions for temperature, relative humidity and light for spray inoculation. In both experiments, seeds were germinated directly in potting mix (PRO-MIX BX General Purpose; Premier Tech Growers and Consumers Inc.) in 3.5 inches diameter square pots (four seeds per pot; Kord Products).

For spray inoculation, the primary leaf (counting from the bottom) of seven-day-old wheat and barley plants was sprayed till run off on the abaxial and adaxial surfaces with water-based bacterial inocula (OD_600_ of 0.5) or sterile deionized water as mock control. After leaves were dry, pots with plants were transferred to trays filled with approximately 0.5 inch of tap water. Trays were then covered for four days to promote high relative humidity, which facilitates infection via spray inoculation. Plants were kept for two additional days in greenhouse conditions after removing the covers and development of water soaking symptoms was evaluated at six days post inoculation. Individual inoculated leaves were taped to transparency film sheets and pictures were taken using a LED light box (Tikteck). When appropriate, the total and symptomatic leaf areas were measured via the ImageJ software [59] by using a set scale. A standardized length of 5 cm from the top to bottom of each inoculated leaf was used for individual measurements. The percentage of symptomatic leaf area was then calculated using the obtained areas. The experiment was conducted independently twice, with 23–24 independent plants being inoculated for each treatment in each independent replicate.

For bacterial population quantification in barley following spray inoculation, leaves inoculated as described above were collected at six days post inoculation. The leaves were surface-disinfested by soaking into 70% ethanol for five seconds, followed by two consecutive rinses in sterile deionized water for five seconds each. This was done so that the isolation targeted those bacterial cells that naturally moved and multiplied into the inner leaf tissue. After, two leaf discs were collected at each side of the main leaf vein using a standardized length of 2.5 cm from the top to bottom of each inoculated leaf by using a 2.5 mm diameter punch (Acuderm Inc.). Leaf discs were then flash frozen in liquid nitrogen and stored at -80ºC until processed. For bacterial isolation, leaf discs were ground in 100 µL of sterile deionized water in 2-mL microcentrifuge tubes containing a sterile 5 mm diameter stainless steel bead (QIAGEN) using the TissueLyzer II apparatus (30 Hz for 1 minute; QIAGEN). Samples were then transferred to 96-well plates (Thermos Scientific), diluted by 10-fold serial dilutions and plated as 5 µL drops in triplicates in selective media (NA + CHX + Km for Xtt strains transformed with pPneo-GFP, and NA + CHX + Gm for Xtu strains with miniTn*7* genomic insertion). Colony forming units (CFU) were enumerated after three days of incubation at 28 ºC. The experiment was conducted independently twice, with 23–24 independent plants being inoculated for each treatment in each independent replicate.

For infiltration inoculation, the first two leaves (counting from the bottom) of fourteen-day-old plants (wheat and barley) were inoculated using needleless syringes. Bacterial strains were suspended in sterile deionized water (OD_600_ of 0.1) for inoculation. Control plants (mock) were inoculated with sterile deionized water. Three to four independent plants were inoculated for each treatment, and experiments were conducted independently at least twice. Development of symptoms was recorded three days post inoculation (dpi), in which lesions associated with pathogenicity were recorded (water soaking – disease development; chlorosis – non-host response; no symptom – no pathogenicity). Pictures of inoculated leaves were taken at 3 dpi by placing them on top of a black non-reflective photography display board (Neewer) and using the camera of a cell phone mounted on a tripod (UBeesize) with a LED cell phone circle light (QIAYA). Germination, growth, inoculation, and evaluation of the plant species used to screen a cereal genotype diversity panel (S3 and S5 Tables) with Xtt and Xtu strains were performed as described for infiltration inoculation.

Inoculations for bacterial growth curves performed in planta were made via leaf syringe infiltration, as described above, but using a lower optical density of each bacterial suspension (OD_600_ of 0.001). Plants were inoculated in triplicates per time point and treatment, and samples were collected at 2, 24, 72, 120, and 168 hours post inoculation using a 2.5 mm diameter punch (Acuderm Inc.), flash frozen in liquid nitrogen and stored at -80 ºC until processed. Bacterial isolation, serial dilution, plating, and enumeration were performed as described above. Experiments were performed independently twice.

### Whole genome sequencing

Genomic DNA for Xtt strains Colorado, CIX84, UPB545, UPB787, and UPB820, and Xtu strain UPB513, was extracted using Genomic-tip 20/G and the Genomic DNA Buffer Set (QIAGEN) and then sequenced as previously described [60]. Briefly, PacBio long-read, single-molecule real-time sequencing was used to sequence extracted genomic DNAs, and assembly was performed via Flye v2.4 [61] for strains CIX84 and UPB545, and via HGAP v4 (PacBio) for strains Colorado, UPB787, UPB820, and UPB513 using default settings. Whole genomes were deposited at GenBank under accession numbers CP159781 for Xtt UPB545, CP159782 for Xtt CIX84, and JBEFAI000000000 for Xtu UPB513 (S9 Table). Availability of the genomes of Xtt strains Colorado, UPB787, and UPB820 is shown in S9 Table.

### Pangenome analyses to identify core and unique proteins among Xtt and Xtu

Pangenome analysis to identify core and unique proteins encoded by all analyzed Xtt and Xtu strains (S1-S2 and S9 Tables) was performed by comparing the gene presence/absence output matrix obtained when analyzing genomes via Panaroo v1.3.3, a tool designed to perform core and pangenome comparisons of prokaryotes [29]. Core proteins are determined here as proteins encoded by all strains in both pathovars translucens and undulosa. On the other hand, unique proteins of a specific pathovar are determined here as proteins encoded by all strains of a specific pathovar but are not encoded by any strain of the other pathovar. The proteome of a representative strain of each pathovar was searched for secretion signals using SignalP v6.0 to predict secreted proteins [62]. Proteomes were obtained by annotating genomes via Prokka v1.14.6 (see below) [63]. SignalP results were then used to identify which core and unique proteins of Xtt and Xtu are predicted to be secreted. Proteins in which a secretion signal was identified but were predicted to locate to the outer membrane or periplasm were excluded when calculating the percentage of secreted proteins within core and unique proteins. Additional genes encoded uniquely by subsets of strains belonging to one pathovar or the other were also identified and analyzed as described above to compare percentages of uniquely secreted proteins in each pathovar via statistical analysis (described below). Hypothetical proteins were not considered to calculate the proportion of secreted proteins in the core genome but were considered when performing this analysis in genes found uniquely in pathovars translucens or undulosa. We manually curated secretion of proteins annotated as hypothetical by searching for encoded functions using the blastp algorithm (<https://blast.ncbi.nlm.nih.gov/Blast.cgi?PAGE=Proteins>) and retrieving the best hit for each searched protein.

### Phylogenomic and phylogenetic analyses

Phylogenomic trees depicting the evolutionary relationships of examined *Xanthomonas* species for host range comparisons (Figs 1A and S1A) were constructed from well-supported 1780 single-copy orthologous genes (SCOs) trees. SCOs were determined via OrthoFinder [64], and individual SCO phylogenies were constructed by first aligning via *mafft* v7.487 [65], trimming via *ClipKIT* v1.3.0 [66], and building the phylogeny via *IQ-TREE* v2.2.6 with 1,000 ultrafast bootstrap iterations [67]. Individual SCO trees with at least 75% average node support were concatenated for a multigene phylogenomic tree by applying best predicted evolutionary models to each SCO partition.

All other whole-genome phylogenies were performed based on core genome alignment. Genomes of interest were annotated via Prokka v1.14.6 specifying the appropriate genus and species with the “--genus” and “--species” options, respectively, and otherwise with default settings [63], followed by core genome alignment using Panaroo v1.3.3 with a core threshold of 0.98 and option “—clean-mode strict” [29]. Alignments were then filtered for recombination by first using maximum likelihood recombination inference through ClonalFrameML v1.12 [68] followed by alignment masking with maskrc-svg v0.5 (https://github.com/kwongj/maskrc-svg), and trees were built using IQ-TREE v2.2.2.7 with 5,000 ultrafast bootstrap replicates.

A phylogenetic tree for the *xopAL1* locus neighborhood, which includes four upstream and five downstream genes of *xopAL1*, was constructed by first running Parsnp v1.7.4 [69] to align the nucleotide sequence of this neighborhood from the reference strain Xtt UPB820 (extracted with the GetFasta command from the BEDTools suite) [70] with the full genomes of the other selected assemblies (S9 Table), then converting the Parsnp output to a FASTA alignment using Harvesttools v1.2 [69], and finally inferring a tree with IQ-TREE v2.2.2.7 using 5,000 ultrafast bootstrap replicates. The same method was used to generate phylogenetic trees of the *xopE5* (six upstream and five downstream genes) and *hopW1* (five upstream and seven downstream genes) loci neighborhoods. An amino acid sequence-based phylogenetic tree of the *xopAJ* locus neighborhood (six upstream and four downstream genes of *xopAJ*) was generated by identifying orthologs of each of these genes in the genomes of interest via OrthoFinder v2.5.5, aligning them individually with MAFFT v7.520 using default parameters. Poorly aligned regions were removed from alignments using Trimal v1.5.0 using the ‘-automated1’ method [71]. A concatenated locus tree was inferred using IQ-TREE v2.2.2.7 with 1,000 ultrafast bootstrap replicates and gene concordance factors were calculated for each node.

Phylogenetic trees for individual effector genes were constructed by first identifying orthologs to these genes in Xtt UPB820 via OrthoFinder v2.5.5, extracting the focal sequences with BEDTools GetFasta, aligning them with MAFFT v7.520, and inferring trees using IQ-TREE v2.2.2.7 with 1,000 ultrafast bootstrap replicates. *xopAL1* encoded by the outgroup representative *Ralstonia solanacearum* strain 10314 was included for the phylogenetic tree of *xopAL1*. The phylogenetic trees of *xopE5*, *hopW1*, and *xopAJ* were constructed via the same method but using *Paraburkholderia nemoris* strain RL18–011-BIC-A and *Ralstonia mannitolilytica* strain LMG 18090 as outgroups for *xopE5*, *Pseudomonas amygdali* strain CFBP 1650 and *Erwinia psidii* strain LPF 534 as outgroups for *hopW1*, and *Paracidovorax citrulli* strains AAC00–1 and DSM 17060 as outgroups for *xopAJ*.

Classification of strains of *X. translucens* pv. translucens in the K0, K1, and K2 sub-genomic groups, as determined by life identification numbers (LINs) calculated with LINbase, was performed as previously described [30].

### Identification of Type III secretion system effector repertoires

The effector repertoires of strains of *X. translucens* were identified as previously described via BLAST 2.8.1 + blastx algorithm [30], excluding transcription activator-like effectors (TALEs). Briefly, a database of known *Xanthomonas* spp. Type III secretion system effectors (https://euroxanth.ipn.pt/doku.php?id=bacteria:t3e:effectors) was used as subject to search the query genomes. Results were filtered for hits with coverage of over 200 amino acids and percent amino acid identity of at least 60%. TALEs were identified and classified using AnnoTALE v1.5 [31]. The effector repertoire heat map and the Type III effector cluster dendrogram (hierarchical clustering) figures were generated using the ComplexHeatmap [72] and RColorBrewer (https://cran.r-project.org/web/packages/RColorBrewer/index.html) packages in R v4.1.0 [73].

### RNA-sequencing and analysis

To determine wheat response to XopAL1, an RNA-seq analysis was performed. Wheat plants were grown as described above and inoculated with Xtt UPB820 WT, Xtu UPB513 WT, Xtt UPB820 ∆*xopAL1,* Xtu UPB513 miniTn*7*::*xopAL1,* or a water (mock) control. Each treatment had three independent replicates, and bacterial inoculations were performed at an OD_600_ of 0.001. Tissue was collected 24 hours post inoculation, flash frozen in liquid nitrogen, and homogenized using a sterile 5 mm diameter stainless steel bead (QIAGEN) using the TissueLyzer II apparatus (30 Hz for 1 minute; QIAGEN). Total RNA was extracted using TRIzol (Thermo Scientific) and DNA was removed using the TURBO DNA-free kit (Thermo Scientific) following manufacturer’s guidelines. Plant and 16S ribosomal depletion was performed and total RNA libraries were constructed using random oligos. RNA libraries were multiplexed and sequenced using a NextSeq 2000 (Illumina) with 150-bp paired-end reads and a target of 30 million reads per sample.

RNA-seq analysis was performed using the Nextflow/nf-core “rnaseq” pipeline v3.10.1 [74], using the *Triticum aestivum* L. cv. ‘Chinese Spring’ reference genome (version 2.1) obtained from Phytozome. RNA-seq data were processed using the nf-core RNA-seq pipeline implemented with Nextflow. This pipeline included adapter trimming with TrimGalore! v0.6.5 (<https://www.bioinformatics.babraham.ac.uk/projects/trim_galore/>), alignment of reads to the wheat genome with STAR v2.7.10b [75], and transcript abundance estimation with Salmon v1.10.1 [76]. Quality metrics for each step were aggregated using MultiQC v1.14 [77]. The read count Table output by the pipeline was used to identify differentially expressed genes using the DESeq2 package [78]. Genes with an adjusted *P* value (FDR) < 0.05 were considered significantly differentially expressed. Genes with a log₂ fold change (log₂FC) > 0 were defined as upregulated, while those with log₂FC < 0 were classified as downregulated. The raw RNA-seq fastq files are available at GenBank under accession number GSE299780.

Normalized gene counts produced from DESeq2 were used to generate a principal component analysis (PCA) plot using ggplot2 in R [79]. Gene ontology (GO) analysis was performed to identify enriched biological functions among differentially expressed genes. GO term annotations for each gene in the wheat ‘Chinese Spring’ reference genome were obtained from Phytozome (<https://phytozome-next.jgi.doe.gov>). Enrichment analysis was conducted separately for upregulated and downregulated genes using the Gene Ontology Resource (<http://geneontology.org>) [80–82]. Only significantly overrepresented GO terms (adjusted *P* value < 0.05) were considered enriched among downregulated or upregulated genes. Selected GO terms related to plant defense and photosynthesis were visualized using a Cleveland dot plot generated in R with ggplot2.

### Chlorophyll concentration measurement in wheat leaves

Xtt and Xtu strains were suspended in sterile deionized water (OD_600_ of 0.1) and inoculated into fourteen-day-old wheat leaves (*Triticum aestivum* L. genotype ‘Chinese Spring’; second leaf counting from the bottom) using needless syringes as described above for infiltration inoculation. Control plants (mock) were inoculated with sterile deionized water. At three days post inoculation, leaves were collected and the chlorophyll concentration in inoculated areas was measured in triplicates in each leaf using the MC-100 Chlorophyll Concentration Meter (Apogee Instruments, Inc.). Chlorophyll concentration was measured as micromoles per square meter (µmol m^-2^) of leaf using the manufacturer’s specific settings for wheat and the standard operational protocol for the instrument. The experiment was conducted independently twice, with six to nine independent plants being inoculated for each treatment in each independent replicate.

### Identification and visualization of genomic neighborhoods

To identify the *xopAL1*, *xopAJ*, *xopE5*, and *hopW1* genomic neighborhoods in *Xanthomonas* spp., Prokka-annotated genomes were aligned and visualized via progressiveMauve using default settings [83] to determine flanking genes and neighborhoods. Genomes were then uploaded to the Benchling cloud-based platform (https://www.benchling.com) to extract the sequences of genomic neighborhoods in GBK format, which were submitted to cluster comparison analysis via Clinker v0.0.23 (default settings) to generate figures [84].

### Protein alignment and visualization

Amino acid sequence alignment of representative sequences of XopAL1 within *Xanthomonas* spp. was performed via the T-Coffee Multiple Sequence Alignment Server [85] and visualized using pyBoxShade (https://github.com/mdbaron42/pyBoxshade). Percent coverage and similarity were determined via the blastp algorithm.

### Data analysis and visualization

Data from number of TAL effectors encoded by Xtt and Xtu were compared by two-tailed Student’s *t*-test. Data from number of proteins in the effector repertoires of both subgroups (excluding TAL effectors), from bacterial growth in barley leaves inoculated via spray at six days post inoculation, and from chlorophyll concentration in wheat leaves inoculated via infiltration at three days post inoculation were analyzed by Kruskal-Wallis test followed by Wilcoxon rank-sum test in R v4.1.0. Data from bacterial growth curves in planta upon syringe infiltration were analyzed per time point by one way analysis of variance (ANOVA) followed by Tukey’s post-hoc test in the log-transformed data. Assumptions were tested on the residuals of the model, and the normality of the data was assessed by the Shapiro-Wilk test in R v4.1.0 under the package rstatix (https://rpkgs.datanovia.com/rstatix/). Data from bacterial growth in barley leaves inoculated via spray at four days post inoculation were analyzed via ANOVA followed by Tukey’s post-hoc test upon checking normality of the data. The original data (x) of the percentage of secreted proteins uniquely found in pathovars translucens or undulosa, and of the percentage of symptomatic leaf area in barley leaves inoculated via spray were submitted to a transformation that combines the arcsine and square root functions (y = arcsin(√x/100)). The normality of data was then assessed by the Shapiro-Wilk test, and treatments were analyzed using the two-tailed Student’s *t*-test. Phylogenetic trees were visualized, and mid-point rooted via FigTree v1.4.4. (http://tree.bio.ed.ac.uk/). Genomic neighborhoods were visualized using Clinker. All source (raw) data used for quantitative analyses are available in the Source Data supporting information file.

## Supporting information

S1 Text Supporting text containing the source data for our quantitative analyses. (PDF)

S1 Table Core proteins encoded by *Xanthomonas translucens* pathovars translucens (Xtt) and undulosa (Xtu). (XLSX)

S2 Table Unique encoded proteins identified when comparing *Xanthomonas translucens* pathovars translucens (Xtt) and undulosa (Xtu). (XLSX)

S3 Table Symptom development assessment in a cereal genotype diversity panel inoculated with Xtt and Xtu strains. (PDF)

S4 Table Differentially expressed genes identified in wheat. (XLSX)

S5 Table Symptom development assessment in a subset of the cereal genotype diversity panel inoculated with Xtt and Xtu strains. (PDF)

S6 Table Bacterial strains and plasmids used in this study. (PDF)

S7 Table List of genes deleted in *X. translucens* in this study. (PDF)

S8 Table Oligonucleotide primers used in this study. (PDF)

S9 Table List of genomes used in this study. (PDF)

S1 Fig Phylogenomics and host range of representative *Xanthomonas* spp. with differential host range breadth, and effector repertoires, including TALEs, of *Xanthomonas translucens* pathovars translucens and undulosa. (A) The phylogenomic tree of representative *Xanthomonas* species with whole genome sequences was made from concatenated single-copy orthologous genes (SCOs). The tree was visualized via FigTree, and mid-point rooted. Plant families of natural hosts of each *Xanthomonas* spp. are color-coded and shown next to respective species in the tree (circle for monocots and square for dicots). Genera of host plants for *X. translucens* pv. translucens (*Hordeum*-only) and pv. undulosa are indicated in the figure. Monocots: Ama – *Amatyllidaceae*; Ara – *Araceae*; Asp – *Asparagaceae*; Lil – *Liliaceae*; Mus – *Musaceae*; Poa – *Poaceae*. Dicots: Ana – *Anacardiaceae*; Api – *Apiaceae*; Ara – *Araliceae*; Ast – *Asteraceae*; Beg – *Begoniaceae*; Bet – *Betulaceae*; Bra – *Brassicaceae*; Can – *Cannabaceae*; Eup – *Euphorbiaceae*; Fab – *Fabaceae*; Ger – *Geraniaceae*; Jug – *Juglandaceae*; Lyt – *Lythraceae*; Mal – *Malvaceae*; Oxa – *Oxalidaceae*; Pap – *Papaveraceae*; Ped – *Pedaliaceae*; Phy – *Phyllanthaceae*; Pip – *Piperaceae*; Pla – *Plantaginaceae*; Ros – *Rosaceae*; Rub – *Rubiaceae*; Sal – *Salicaceae*; Sol – *Solanaceae*. ^E^Indicates experimentally determined host plant family. (B) A core genome-based tree of 28 *X. translucens* strains is shown to the left side of the figure, demonstrating specific clustering of each pathovar. The tree was visualized via FigTree, and mid-point rooted. Branches with bootstrap values below 98% are indicated in the tree. Putative Type III secretion system effectors were identified via local Blastx against a database of known *Xanthomonas* effectors. Transcription activator-like effectors (TALEs) were identified and classified using AnnoTALE v.1.5. Inclusion of TALEs in the effector repertoire analysis disrupted the hierarchical clustering of strains within each pathovar according to their encoded repertoires (Type III effector cluster dendrogram; right side of the figure), making them not follow the phylogeny of the species (as shown in the phylogenomic tree; left side of the figure), contrary to what is obtained when TALEs are not included (see Fig 1C). Xtt – *X. translucens* pv. translucens; Xtu – *X. translucens* pv. undulosa.
(TIF)

S2 Fig XopAL1 diversity in *Xanthomonas* spp. (A) Nucleotide-sequence-based phylogenetic trees of *xopAL1* sequences from representative clade I and clade II *Xanthomonas* spp. The tree to the left side of the panel does not contain the truncated *xopAL1* sequences that are encoded by strains of *X. translucens* pv. graminis, which are included in the tree to the right side of the panel. XopAL1 from *Ralstonia solanacearum* strain 10314 was included as an outgroup to root the tree. Trees were visualized via FigTree. Branches with bootstrap values below 98% are shown in each tree. (B) Alignment of representative XopAL1 ortholog sequences within *Xanthomonas* spp. Sequences were aligned via T-Coffee and visualized using pyBoxShade. Different sequences of XopAL1 are denoted by representative strains within each species. Black shading indicates conserved residues; grey shading indicates conservative mutations; and white color indicates divergence among sequences. Amino acid changes within *X. translucens* is indicated by using a bigger font size and the color blue (in comparison to the reference XopAL1 encoded by Xtt UPB820). Sequences represent the entire diversity of XopAL1 within *X. translucens*. (C) Percent coverage and similarity of XopAL1 sequences encoded among representative *Xanthomonas* spp. in comparison to the reference Xtt strain UPB820. Xa - *X. arboricola*; Xhh - *X. hortorum* pv. hederae; Xhp - *X. hortorum* pv. pelargonii; Xcc - *X. campestris* pv. campestris; Xc - *X. campestris*; Xtt - *X. translucens* pv. translucens; Xtp - *X. translucens* pv. pistaciae; Xtpo - *X. translucens* pv. poae; Xtg - *X. translucens* pv. graminis; Rs - *Ralstonia solanacearum*.
(TIF)

S3 Fig The *X. campestris* pv. campestris (Xcc) strain 8004 XopAL1 ortholog triggers wheat non-host response. Symptom development in wheat (*Triticum aestivum* L. genotype ‘Chinese Spring’) leaves inoculated via syringe infiltration with Xtu strains (OD_600_ 0.1) at three days post inoculation. The Xcc 8004 XopAL1 ortholog was expressed in Xtu via a plasmid construct, leading to development of chlorosis or a mix of chlorosis and water soaking when inoculated into wheat leaves. Scale bar is shown in figure. Xtu – *X. translucens* pv. undulosa; Ch. – chlorosis (appear as yellow lesions); W.S. – water soaking (appear as wet, dark lesions due to the dark background).
(TIF)

S4 Fig Schematic overview of the *xopAL1* locus neighborhood types in *Xanthomonas translucens* using Clinker. Among analyzed strains, only Xtt, Xtp CFBP 8304 and Xtpo NCPPB 3711 encode *xopAL1*. Strains of Xtc, Xtt, Xtp CFBP8304 and Xtpo NCPPB 3711 have the same neighborhood, with Xtc sharing all genes except *xopAL1*. Xtg encodes truncated versions of *xopAL1*, which is likely due to the presence of IS5 family transposases. Within the other non-*xopAL1*-encoding strains, there is presence of other genes in place of *xopAL1*. Xtpo, Xta and Xtphlei strains have a similar neighborhood that shares similarities with the neighborhood of Xhy, which encodes additional genes in comparison to all pathovars of *X. translucens* (including transposases). At last, Xtu encodes a neighborhood that is specific to this pathovar. Xtc – *X. translucens* pv. cerealis; Xtg – *X. translucens* pv. graminis; Xtpo – *X. translucens* pv. poae; Xta – *X. translucens* pv. arrhenateri; Xtphlei – *X. translucens* pv. phleipratensis; Xtu – *X. translucens* pv. undulosa; Xtp – *X. translucens* pv. pistaciae; Xtt – *X. translucens* pv. translucens; Xhy – *X. hyacinthi*.
(TIF)

S5 Fig *xopE5* has an ancestral origin in *Xanthomonas translucens* and was lost in Xtu. (A) Tree of the concatenated sequences of the genes flanking *xopE5* in *X. translucens*. The *xopE5* locus neighborhood types are indicated in each branch and correspond to neighborhood types shown in panel B. Red-colored branches indicate subgroups that do not encode this effector gene, with determined loss events being shown (see tree legend). *X. hyacinthi*, a closely related clade I *Xanthomonas* spp., was included as an outgroup to root the tree. (B) Schematic overview of the *xopE5* locus neighborhood types in representative pathovars of *Xanthomonas translucens* and in the closely related species *X. hyacinthi* using Clinker. Among analyzed strains, all Xtt, Xtp, Xtc, and *X. hyacinthi* encode *xopE5*. All these have similar neighborhoods. However, Xtc strains have a smaller neighborhood with partial sequence of conserved genes found in all strains. Within non-*xopE5*-encoding strains, there are no other genes replacing *xopE5*. *X. hyacinthi* encodes two additional genes in comparison to all pathovars of *X. translucens*. (C) Nucleotide-sequence-based phylogenetic tree of *xopE5* sequences from representative clade I and clade II *Xanthomonas* spp. *xopE5* sequences from *Paraburkholderia nemoris* strain RL18–011-BIC-A and *Ralstonia mannitolilytica* strain LMG 18090 were used as outgroups to root the tree. *xopE5* is encoded in a similar neighborhood by the closely related species *X. hyacinthi* (panel B), and this ortholog is closely related to *xopE5* encoded by *X. translucens* pathovars (panel C). Thus, we inferred an ancestral origin of this effector gene in *X. translucens* (i.e., common origin with a shared ancestor with *X. hyacinthi*), and determined subsequent evolutionary losses in *X. translucens* subgroups that do not encode this gene. This includes loss of *xopE5* in Xtu during its divergence from the common ancestor with Xtt. Trees were visualized via Figtree. Branches with bootstrap values below 98% are indicated in each tree. Xtu – *X. translucens* pv. undulosa; Xtt – *X. translucens* pv. translucens; Xtp – *X. translucens* pv. pistaciae; Xtc – *X. translucens* pv. cerealis; Xtg – *X. translucens* pv. graminis; Xta – *X. translucens* pv. arrhenateri; Xtpo – *X. translucens* pv. poae; Xtphlei – *X. translucens* pv. phleipratensis; Xhy – *X. hyacinthi*; Xf – *X. fragariae*; Xhv – *X. hortorum* pv. vitians; Pn – *Paraburkholderia nemoris*; Rm – *Ralstonia mannitolilytica*.
(TIF)

S6 Fig *hopW1* was lost in Xtu and is encoded in a highly rearranged genomic neighborhood. (A) Tree of the concatenated sequences of the genes flanking *hopW1* in *X. translucens*. The *hopW1* locus neighborhood types are indicated in each branch and correspond to neighborhood types shown in panel B. Red-colored branches indicate subgroups that do not encode this effector gene, with determined absence and loss events being shown (see tree legend). *X.* *hyacinthi*, a closely related clade I *Xanthomonas* spp., was included as an outgroup to root the tree. (B) Schematic overview of the *hopW1* locus neighborhood types in representative pathovars of *Xanthomonas translucens* and in the closely related species *X. hyacinthi* using Clinker. Among analyzed strains, all Xtt, Xta LMG 727, and *X. hyacinthi* encode *hopW1*. Xtt strains encode *hopW1* in similar genomic neighborhoods, but which have few differences in comparison to the neighborhood of Xta LMG 727. The latter encodes two additional genes (IS3 family transposases) in comparison to Xtt strains. *X. hyacinthi* encodes *hopW1* in a different genomic neighborhood in comparison to *X. translucens*. Within non-*hopW1*-encoding strains, there is presence of other genes in place of *hopW1*. (C) Nucleotide-sequence-based phylogenetic tree of *hopW1* sequences from representative clade I and clade II *Xanthomonas* spp. *hopW1* sequences from *Pseudomonas amygdali* strain CFBP 1650 and *Erwinia psidii* strain LPF 534 were used as outgroups to root the tree. *hopW1* is encoded in a different neighborhood by the closely related species *X. hyacinthi* (panel B), and this ortholog is distantly related to *hopW1* encoded by *X. translucens* pathovars (panel C). Since the Xta and Xtt orthologs are closely related (panel C) and they share a similar genomic neighborhood (panel B), we inferred an origin in Xta upon divergence from Xtg, and subsequent losses in pathovars that do not encode this effector gene. This includes loss of *hopW1* in Xtu during its divergence from the common ancestor with Xtt. In addition, we observed rearrangements and presence of transposases within this genomic neighborhood in *X. translucens* (see panel B), suggesting that this locus is subject to dynamic evolutionary events. Trees were visualized via Figtree. Branches with bootstrap values below 98% are indicated in each tree. Xtu – *X. translucens* pv. undulosa; Xtc – *X. translucens* pv. cerealis; Xtp – *X. translucens* pv. pistaciae; Xtt – *X. translucens* pv. translucens; Xtpo – *X. translucens* pv. poae; Xtphlei – *X. translucens* pv. phleipratensis; Xta – *X. translucens* pv. arrhenateri; Xtg – *X. translucens* pv. graminis; Xhy – *X. hyacinthi*; Xn – *X. nasturtii*; Xp – *X. populi*; Xc – *X. campestris*; Xe – *X. euvesicatoria*; Pa – *Pseudomonas amygdali*; Ep – *Erwinia psidii*.
(TIF)

S7 Fig Schematic overview of the *xopAJ* locus neighborhood types in *Xanthomonas translucens* and clade II *Xanthomonas* species using Clinker. (A) *xopAJ* locus neighborhood types in *Xanthomonas translucens*. Among analyzed strains, only Xtt encodes *xopAJ*. Xtp CFBP8304 encodes a truncated version of *xopAJ*, which is likely due to the presence of an IS5 family transposase. Within non-*xopAJ*-encoding strains, there are no other genes replacing *xopAJ*. (B) *xopAJ* locus neighborhood types in Xtt and clade II *Xanthomonas* spp. The *xopAJ* locus neighborhood is different between Xtt (clade I *Xanthomonas* spp.) and clade II *Xanthomonas* spp. Xoc and Xb have distinct *xopAJ* locus neighborhood types between themselves and in comparison to other clade II *Xanthomonas* spp. Xe and Xa have similar neighborhood types. All clade II *Xanthomonas* spp. have multiple insertion sequence (IS) elements in their *xopAJ* locus neighborhood types. Xtc – *X. translucens* pv. cerealis; Xtg – *X. translucens* pv. graminis; Xta – *X. translucens* pv. arrhenateri; Xtphlei – *X. translucens* pv. phleipratensis; Xtpo – *X. translucens* pv. poae; Xtu – *X. translucens* pv. undulosa; Xtp – *X. translucens* pv. pistaciae; Xtt – *X. translucens* pv. translucens; Xhy – *X. hyacinthi*; Xoc – *X. oryzae* pv. oryzicola; Xb – *X. bromi*; Xe – *X. euvesicatoria*; *Xa* – *X. axonopodis*.
(TIF)

S8 Fig Phylogenetic tree of *xopAJ* sequences from representative clade I and clade II *Xanthomonas* spp. The tree was visualized via FigTree, and mid-point rooted. Branches with bootstrap values below 98% are shown in the tree. *xopAJ* sequences from *Paracidovorax citrulli* strains AAC00–1 and DSM 17060 were included as outgroups but clustered with *xopAJ* encoded by clade I *Xanthomonas* spp. Pc – *Paracidovorax citrulli*; Xtp – *X. translucens* pv. pistaciae; Xtt – *X. translucens* pv. translucens; Xb – *X. bromi*; Xoc – *X. oryzae* pv. oryzicola; *Xa* – *X. axonopodis*; Xe – *X. euvesicatoria*.
(TIF)

## References

[1] Inoue Y, Vy TTP, Yoshida K, Asano H, Mitsuoka C, Asuke S, et al. Evolution of the wheat blast fungus through functional losses in a host specificity determinant. Science. 2017;357(6346):80–3. doi: 10.1126/science.aam9654 28684523

[2] Gluck-Thaler E, Cerutti A, Perez-Quintero AL, Butchacas J, Roman-Reyna V, Madhavan VN, et al. Repeated gain and loss of a single gene modulates the evolution of vascular plant pathogen lifestyles. Sci Adv. 2020;6(46):eabc4516. doi: 10.1126/sciadv.abc4516 33188025 PMC7673761

[3] Melnyk RA, Hossain SS, Haney CH. Convergent gain and loss of genomic islands drive lifestyle changes in plant-associated Pseudomonas. ISME J. 2019;13(6):1575–88. doi: 10.1038/s41396-019-0372-5 30787396 PMC6776051

[4] Baker RE, Mahmud AS, Miller IF, Rajeev M, Rasambainarivo F, Rice BL, et al. Infectious disease in an era of global change. Nat Rev Microbiol. 2022;20(4):193–205. doi: 10.1038/s41579-021-00639-z 34646006 PMC8513385

[5] Barrett LG, Kniskern JM, Bodenhausen N, Zhang W, Bergelson J. Continua of specificity and virulence in plant host-pathogen interactions: causes and consequences. New Phytol. 2009;183(3):513–29. doi: 10.1111/j.1469-8137.2009.02927.x 19563451

[6] Jacques M-A, Arlat M, Boulanger A, Boureau T, Carrère S, Cesbron S, et al. Using ecology, physiology, and genomics to understand host specificity in xanthomonas. Annu Rev Phytopathol. 2016;54:163–87. doi: 10.1146/annurev-phyto-080615-100147 27296145

[7] Jacobs JM, Pesce C, Lefeuvre P, Koebnik R. Comparative genomics of a cannabis pathogen reveals insight into the evolution of pathogenicity in Xanthomonas. Front Plant Sci. 2015;6:431. doi: 10.3389/fpls.2015.00431 26136759 PMC4468381

[8] Dubrow ZE, Carpenter SCD, Carter ME, Grinage A, Gris C, Lauber E, et al. Cruciferous weed isolates of xanthomonas campestris yield insight into pathovar genomic relationships and genetic determinants of host and tissue specificity. Mol Plant Microbe Interact. 2022;35(9):791–802. doi: 10.1094/MPMI-01-22-0024-R 35536128

[9] Escalon A, Javegny S, Vernière C, Noël LD, Vital K, Poussier S, et al. Variations in type III effector repertoires, pathological phenotypes and host range of Xanthomonas citri pv. citri pathotypes. Mol Plant Pathol. 2013;14(5):483–96. doi: 10.1111/mpp.12019 23437976 PMC6638789

[10] Gochez AM, Minsavage GV, Potnis N, Canteros BI, Stall RE, Jones JB. A functional Xop AG homologue in Xanthomonas fuscans pv. aurantifolii strain C limits host range. Plant Pathology. 2015;64(5):1207–14. doi: 10.1111/ppa.12361

[11] Ferrante P, Clarke CR, Cavanaugh KA, Michelmore RW, Buonaurio R, Vinatzer BA. Contributions of the effector gene hopQ1-1 to differences in host range between Pseudomonas syringae pv. phaseolicola and P. syringae pv. tabaci. Mol Plant Pathol. 2009;10(6):837–42. doi: 10.1111/j.1364-3703.2009.00577.x 19849789 PMC6640246

[12] Schwartz AR, Potnis N, Timilsina S, Wilson M, Patané J, Martins J Jr, et al. Phylogenomics of Xanthomonas field strains infecting pepper and tomato reveals diversity in effector repertoires and identifies determinants of host specificity. Front Microbiol. 2015;6:535. doi: 10.3389/fmicb.2015.00535 26089818 PMC4452888

[13] Morris CE, Moury B. Revisiting the concept of host range of plant pathogens. Annu Rev Phytopathol. 2019;57:63–90. doi: 10.1146/annurev-phyto-082718-100034 31082307

[14] Alvarez-Martinez CE, Sgro GG, Araujo GG, Paiva MRN, Matsuyama BY, Guzzo CR, et al. Secrete or perish: the role of secretion systems in Xanthomonas biology. Comput Struct Biotechnol J. 2020;19:279–302. doi: 10.1016/j.csbj.2020.12.020 33425257 PMC7777525

[15] Timilsina S, Potnis N, Newberry EA, Liyanapathiranage P, Iruegas-Bocardo F, White FF, et al. Xanthomonas diversity, virulence and plant-pathogen interactions. Nat Rev Microbiol. 2020;18(8):415–27. doi: 10.1038/s41579-020-0361-8 32346148

[16] Pesce C, Jacobs JM, Berthelot E, Perret M, Vancheva T, Bragard C, et al. Comparative genomics identifies a novel conserved protein, HpaT, in proteobacterial type III secretion systems that do not possess the putative translocon protein HrpF. Front Microbiol. 2017;8:1177. doi: 10.3389/fmicb.2017.01177 28694803 PMC5483457

[17] Sapkota S, Mergoum M, Liu Z. The translucens group of Xanthomonas translucens: complicated and important pathogens causing bacterial leaf streak on cereals. Mol Plant Pathol. 2020;21(3):291–302. doi: 10.1111/mpp.12909 31967397 PMC7036361

[18] Friskop A, Green A, Ransom J, Liu Z, Knodel J, Hansen B, et al. Increase of bacterial leaf streak in hard red spring wheat in North dakota and yield loss considerations. Phytopathology. 2023;113(11):2103–9. doi: 10.1094/PHYTO-08-22-0282-SA 36399026

[19] Ledman KE, Curland RD, Ishimaru CA, Dill-Macky R. Xanthomonas translucens pv. undulosa identified on common weedy grasses in naturally infected wheat fields in minnesota. Phytopathology. 2021;111(7):1114–21. doi: 10.1094/PHYTO-08-20-0337-R 33225830

[20] Jones LR, Johnson AG, Reddy CS. Bacterial blights of barley and certain other cereals. Science. 1916;44(1134):432–3. doi: 10.1126/science.44.1134.432 17811971

[21] Smith EF, Jones LR, Reddy CS. The black chaff of wheat. Science. 1919;50(1280):48. doi: 10.1126/science.50.1280.48 17801665

[22] Roman-Reyna V, Heiden N, Butchacas J, Toth H, Cooperstone JL, Jacobs JM. The timing of bacterial mesophyll infection shapes the leaf chemical landscape. Microbiol Spectr. 2024;12(4):e0413823. doi: 10.1128/spectrum.04138-23 38426767 PMC10986523

[23] Bragard C, Verdier V, Maraite H. Genetic diversity among xanthomonas campestris strains pathogenic for small grains. Appl Environ Microbiol. 1995;61(3):1020–6. doi: 10.1128/aem.61.3.1020-1026.1995 16534952 PMC1388384

[24] Bragard C, Singer E, Alizadeh A, Vauterin L, Maraite H, Swings J. Xanthomonas translucens from small grains: diversity and phytopathological relevance. Phytopathology. 1997;87(11):1111–7. doi: 10.1094/PHYTO.1997.87.11.1111 18945007

[25] Curland RD, Saad YS, Ledman KE, Ishimaru CA, Dill-Macky R. First report of bacterial leaf streak caused by Xanthomonas translucens pv. undulosa on intermediate wheatgrass (Thinopyrum intermedium) in Minnesota. Plant Disease. 2020;104(1):279–279. doi: 10.1094/pdis-04-19-0797-pdn

[26] Curland RD, Hallada KR, Ledman KE, Dill-Macky R. First report of bacterial leaf streak caused by Xanthomonas translucens pv. undulosa on cultivated wild rice (Zizania palustris) in Minnesota. Plant Disease. 2021;105(9):2711. doi: 10.1094/pdis-02-21-0407-pdn

[27] Rademaker JLW, Norman DJ, Forster RL, Louws FJ, Schultz MH, de Bruijn FJ. Classification and identification of Xanthomonas translucens isolates, including those pathogenic to ornamental asparagus. Phytopathology. 2006;96(8):876–84. doi: 10.1094/PHYTO-96-0876 18943753

[28] Goettelmann F, Roman-Reyna V, Cunnac S, Jacobs JM, Bragard C, Studer B, et al. Complete genome assemblies of all Xanthomonas translucens pathotype strains reveal three genetically distinct clades. Front Microbiol. 2022;12:817815. doi: 10.3389/fmicb.2021.817815 35310401 PMC8924669

[29] Tonkin-Hill G, MacAlasdair N, Ruis C, Weimann A, Horesh G, Lees JA, et al. Producing polished prokaryotic pangenomes with the Panaroo pipeline. Genome Biol. 2020;21(1):180. doi: 10.1186/s13059-020-02090-4 32698896 PMC7376924

[30] Heiden N, Roman-Reyna V, Curland RD, Dill-Macky R, Jacobs JM. Comparative genomics of barley-infecting xanthomonas translucens shows overall genetic similarity but globally distributed virulence factor diversity. Phytopathology. 2023;113(11):2056–61. doi: 10.1094/PHYTO-04-22-0113-SC 35727947

[31] Grau J, Reschke M, Erkes A, Streubel J, Morgan RD, Wilson GG, et al. AnnoTALE: bioinformatics tools for identification, annotation, and nomenclature of TALEs from Xanthomonas genomic sequences. Sci Rep. 2016;6:21077. doi: 10.1038/srep21077 26876161 PMC4753510

[32] Peng Z, Hu Y, Zhang J, Huguet-Tapia JC, Block AK, Park S, et al. Xanthomonas translucens commandeers the host rate-limiting step in ABA biosynthesis for disease susceptibility. Proc Natl Acad Sci U S A. 2019;116(42):20938–46. doi: 10.1073/pnas.1911660116 31575748 PMC6800315

[33] Erkes A, Reschke M, Boch J, Grau J. Evolution of transcription activator-like effectors in Xanthomonas oryzae. Genome Biol Evol. 2017;9(6):1599–615. doi: 10.1093/gbe/evx108 28637323 PMC5512977

[34] Curland RD, Gao L, Hirsch CD, Ishimaru CA. Localized genetic and phenotypic diversity of xanthomonas translucens associated with bacterial leaf streak on wheat and barley in Minnesota. Phytopathology. 2020;110(2):257–66. doi: 10.1094/PHYTO-04-19-0134-R 31448998

[35] Curland RD, Gao L, Bull CT, Vinatzer BA, Dill-Macky R, Van Eck L, et al. Genetic Diversity and Virulence of Wheat and Barley Strains of Xanthomonas translucens from the Upper Midwestern United States. Phytopathology. 2018;108(4):443–53. doi: 10.1094/PHYTO-08-17-0271-R 29165007

[36] Stace CA. Triticale: a case of nomenclatural mistreatment. Taxon. 1987;36(2):445–52. doi: 10.2307/1221447

[37] Adlung N, Prochaska H, Thieme S, Banik A, Blüher D, John P, et al. Non-host resistance induced by the xanthomonas effector XopQ is widespread within the genus nicotiana and functionally depends on EDS1. Front Plant Sci. 2016;7:1796. doi: 10.3389/fpls.2016.01796 27965697 PMC5127841

[38] Balint-Kurti P. The plant hypersensitive response: concepts, control and consequences. Mol Plant Pathol. 2019;20(8):1163–78. doi: 10.1111/mpp.12821 31305008 PMC6640183

[39] Vera Cruz CM, Bai J, Ona I, Leung H, Nelson RJ, Mew TW, et al. Predicting durability of a disease resistance gene based on an assessment of the fitness loss and epidemiological consequences of avirulence gene mutation. Proc Natl Acad Sci U S A. 2000;97(25):13500–5. doi: 10.1073/pnas.250271997 11095723 PMC17604

[40] Clavijo F, Barrera C, Bencic A, Croce V, Jacobs JM, Bernal AJ, et al. Complete genome sequence resource for Xanthomonas translucens pv. undulosa MAI5034, a wheat pathogen from Uruguay. Phytopathology. 2022;112(9):2036–9. 35559654 10.1094/PHYTO-01-22-0025-A

[41] McCutcheon JP, Moran NA. Extreme genome reduction in symbiotic bacteria. Nat Rev Microbiol. 2011;10(1):13–26. doi: 10.1038/nrmicro2670 22064560

[42] Roman-Reyna V, Sharma A, Toth H, Konkel Z, Omiotek N, Murthy S, et al. Live tracking of a plant pathogen outbreak reveals rapid and successive, multidecade plasmid reduction. mSystems. 2024;9(2):e0079523. doi: 10.1128/msystems.00795-23 38275768 PMC10878067

[43] Abrahamian P, Timilsina S, Minsavage GV, Kc S, Goss EM, Jones JB, et al. The Type III Effector AvrBsT Enhances Xanthomonas perforans Fitness in Field-Grown Tomato. Phytopathology. 2018;108(12):1355–62. doi: 10.1094/PHYTO-02-18-0052-R 29905507

[44] Oggenfuss U, Badet T, Wicker T, Hartmann FE, Singh NK, Abraham L, et al. A population-level invasion by transposable elements triggers genome expansion in a fungal pathogen. Elife. 2021;10:e69249. doi: 10.7554/eLife.69249 34528512 PMC8445621

[45] Krishnan P, Meile L, Plissonneau C, Ma X, Hartmann FE, Croll D, et al. Transposable element insertions shape gene regulation and melanin production in a fungal pathogen of wheat. BMC Biol. 2018;16(1):78. doi: 10.1186/s12915-018-0543-2 30012138 PMC6047131

[46] Ferreira RM, de Oliveira ACP, Moreira LM, Belasque J Jr, Gourbeyre E, Siguier P, et al. A TALE of transposition: Tn3-like transposons play a major role in the spread of pathogenicity determinants of Xanthomonas citri and other xanthomonads. mBio. 2015;6(1):e02505-14. doi: 10.1128/mBio.02505-14 25691597 PMC4337579

[47] Zhao B, Ardales EY, Raymundo A, Bai J, Trick HN, Leach JE, et al. The avrRxo1 gene from the rice pathogen Xanthomonas oryzae pv. oryzicola confers a nonhost defense reaction on maize with resistance gene Rxo1. Mol Plant Microbe Interact. 2004;17(7):771–9. doi: 10.1094/MPMI.2004.17.7.771 15242171

[48] Popov G, Fraiture M, Brunner F, Sessa G. Multiple Xanthomonas euvesicatoria type III effectors inhibit flg22-triggered immunity. Mol Plant Microbe Interact. 2016;29(8):651–60. doi: 10.1094/MPMI-07-16-0137-R 27529660

[49] Liu H, Lu C, Li Y, Wu T, Zhang B, Liu B, et al. The bacterial effector AvrRxo1 inhibits vitamin B6 biosynthesis to promote infection in rice. Plant Commun. 2022;3(3):100324. doi: 10.1016/j.xplc.2022.100324 35576156 PMC9251433

[50] Liu L, Li Y, Xu Z, Chen H, Zhang J, Manion B, et al. The Xanthomonas type III effector XopAP prevents stomatal closure by interfering with vacuolar acidification. J Integr Plant Biol. 2022;64(10):1994–2008. doi: 10.1111/jipb.13344 35972796

[51] Jones JDG, Dangl JL. The plant immune system. Nature. 2006;444(7117):323–9. doi: 10.1038/nature05286 17108957

[52] Song WY, Wang GL, Chen LL, Kim HS, Pi LY, Holsten T, et al. A receptor kinase-like protein encoded by the rice disease resistance gene, Xa21. Science. 1995;270(5243):1804–6. doi: 10.1126/science.270.5243.1804 8525370

[53] Pruitt RN, Schwessinger B, Joe A, Thomas N, Liu F, Albert M, et al. The rice immune receptor XA21 recognizes a tyrosine-sulfated protein from a Gram-negative bacterium. Sci Adv. 2015;1(6):e1500245. doi: 10.1126/sciadv.1500245 26601222 PMC4646787

[54] Littlejohn GR, Breen S, Smirnoff N, Grant M. Chloroplast immunity illuminated. New Phytol. 2021;229(6):3088–107. doi: 10.1111/nph.17076 33206379

[55] Levy AA, Feldman M. Evolution and origin of bread wheat. Plant Cell. 2022;34(7):2549–67. 35512194 10.1093/plcell/koac130 PMC9252504

[56] Feldman M, Levy AA. Evolution of wheat under cultivation. Wheat evolution and domestication. Springer International Publishing; 2023. 605–63. doi: 10.1007/978-3-031-30175-9_13

[57] Newton AC, Flavell AJ, George TS, Leat P, Mullholland B, Ramsay L, et al. Crops that feed the world 4. Barley: a resilient crop? Strengths and weaknesses in the context of food security. Food Sec. 2011;3(2):141–78. doi: 10.1007/s12571-011-0126-3

[58] Slafer GA, Savin R. Comparative performance of barley and wheat across a wide range of yielding conditions. Does barley outyield wheat consistently in low-yielding conditions?. Euro J Agronomy. 2023;143:126689. doi: 10.1016/j.eja.2022.126689

[59] Schneider CA, Rasband WS, Eliceiri KW. NIH Image to ImageJ: 25 years of image analysis. Nat Methods. 2012;9(7):671–5. doi: 10.1038/nmeth.2089 22930834 PMC5554542

[60] Roman-Reyna V, Luna EK, Pesce C, Vancheva T, Chang C, Ziegle J, et al. Genome resource of barley bacterial blight and leaf streak pathogen Xanthomonas translucens pv. translucens strain UPB886. Plant Dis. 2020;104(1):13–5. 31660797 10.1094/PDIS-05-19-1103-A

[61] Kolmogorov M, Yuan J, Lin Y, Pevzner PA. Assembly of long, error-prone reads using repeat graphs. Nat Biotechnol. 2019;37(5):540–6. doi: 10.1038/s41587-019-0072-8 30936562

[62] Teufel F, Almagro Armenteros JJ, Johansen AR, Gíslason MH, Pihl SI, Tsirigos KD, et al. SignalP 6.0 predicts all five types of signal peptides using protein language models. Nat Biotechnol. 2022;40(7):1023–5. doi: 10.1038/s41587-021-01156-3 34980915 PMC9287161

[63] Seemann T. Prokka: rapid prokaryotic genome annotation. Bioinformatics. 2014;30(14):2068–9. doi: 10.1093/bioinformatics/btu153 24642063

[64] Emms DM, Kelly S. OrthoFinder: phylogenetic orthology inference for comparative genomics. Genome Biol. 2019;20(1):238. doi: 10.1186/s13059-019-1832-y 31727128 PMC6857279

[65] Katoh K, Misawa K, Kuma K, Miyata T. MAFFT: a novel method for rapid multiple sequence alignment based on fast Fourier transform. Nucleic Acids Res. 2002;30(14):3059–66. doi: 10.1093/nar/gkf436 12136088 PMC135756

[66] Steenwyk JL, Buida TJ 3rd, Li Y, Shen X-X, Rokas A. ClipKIT: a multiple sequence alignment trimming software for accurate phylogenomic inference. PLoS Biol. 2020;18(12):e3001007. doi: 10.1371/journal.pbio.3001007 33264284 PMC7735675

[67] Minh BQ, Schmidt HA, Chernomor O, Schrempf D, Woodhams MD, von Haeseler A, et al. IQ-TREE 2: new models and efficient methods for phylogenetic inference in the genomic era. Mol Biol Evol. 2020;37(5):1530–4. doi: 10.1093/molbev/msaa015 32011700 PMC7182206

[68] Didelot X, Wilson DJ. ClonalFrameML: efficient inference of recombination in whole bacterial genomes. PLoS Comput Biol. 2015;11(2):e1004041. doi: 10.1371/journal.pcbi.1004041 25675341 PMC4326465

[69] Treangen TJ, Ondov BD, Koren S, Phillippy AM. The Harvest suite for rapid core-genome alignment and visualization of thousands of intraspecific microbial genomes. Genome Biol. 2014;15(11):524. doi: 10.1186/s13059-014-0524-x 25410596 PMC4262987

[70] Quinlan AR, Hall IM. BEDTools: a flexible suite of utilities for comparing genomic features. Bioinformatics. 2010;26(6):841–2. doi: 10.1093/bioinformatics/btq033 20110278 PMC2832824

[71] Capella-Gutiérrez S, Silla-Martínez JM, Gabaldón T. trimAl: a tool for automated alignment trimming in large-scale phylogenetic analyses. Bioinformatics. 2009;25(15):1972–3. doi: 10.1093/bioinformatics/btp348 19505945 PMC2712344

[72] Gu Z, Eils R, Schlesner M. Complex heatmaps reveal patterns and correlations in multidimensional genomic data. Bioinformatics. 2016;32(18):2847–9. doi: 10.1093/bioinformatics/btw313 27207943

[73] Ihaka R, Gentleman R. R: A language for data analysis and graphics. J Comput Graph Stat. 1996;5:299–314.

[74] Ewels PA, Peltzer A, Fillinger S, Patel H, Alneberg J, Wilm A, et al. The nf-core framework for community-curated bioinformatics pipelines. Nat Biotechnol. United States. 2020. 276–8. 10.1038/s41587-020-0439-x 32055031

[75] Dobin A, Davis CA, Schlesinger F, Drenkow J, Zaleski C, Jha S, et al. STAR: ultrafast universal RNA-seq aligner. Bioinformatics. 2013;29(1):15–21. doi: 10.1093/bioinformatics/bts635 23104886 PMC3530905

[76] Patro R, Duggal G, Love MI, Irizarry RA, Kingsford C. Salmon provides fast and bias-aware quantification of transcript expression. Nat Methods. 2017;14(4):417–9. doi: 10.1038/nmeth.4197 28263959 PMC5600148

[77] Ewels P, Magnusson M, Lundin S, Käller M. MultiQC: summarize analysis results for multiple tools and samples in a single report. Bioinformatics. 2016;32(19):3047–8. doi: 10.1093/bioinformatics/btw354 27312411 PMC5039924

[78] Love MI, Huber W, Anders S. Moderated estimation of fold change and dispersion for RNA-seq data with DESeq2. Genome Biol. 2014;15(12):550. doi: 10.1186/s13059-014-0550-8 25516281 PMC4302049

[79] Wickham H. ggplot2: elegant graphics for data analysis. Springer-Verlag New York; 2016.

[80] Carbon S, Ireland A, Mungall CJ, Shu S, Marshall B, Lewis S, et al. AmiGO: online access to ontology and annotation data. Bioinformatics. 2009;25(2):288–9. doi: 10.1093/bioinformatics/btn615 19033274 PMC2639003

[81] Thomas PD, Hill DP, Mi H, Osumi-Sutherland D, Van Auken K, Carbon S, et al. Gene Ontology Causal Activity Modeling (GO-CAM) moves beyond GO annotations to structured descriptions of biological functions and systems. Nat Genet. 2019;51(10):1429–33. doi: 10.1038/s41588-019-0500-1 31548717 PMC7012280

[82] Thomas PD, Ebert D, Muruganujan A, Mushayahama T, Albou L-P, Mi H. PANTHER: making genome-scale phylogenetics accessible to all. Protein Sci. 2022;31(1):8–22. doi: 10.1002/pro.4218 34717010 PMC8740835

[83] Darling AE, Mau B, Perna NT. progressiveMauve: multiple genome alignment with gene gain, loss and rearrangement. PLoS One. 2010;5(6):e11147. doi: 10.1371/journal.pone.0011147 20593022 PMC2892488

[84] Gilchrist CLM, Chooi Y-H. clinker & clustermap.js: automatic generation of gene cluster comparison figures. Bioinformatics. 2021;37(16):2473–5. doi: 10.1093/bioinformatics/btab007 33459763

[85] Di Tommaso P, Moretti S, Xenarios I, Orobitg M, Montanyola A, Chang J-M, et al. T-Coffee: a web server for the multiple sequence alignment of protein and RNA sequences using structural information and homology extension. Nucleic Acids Res. 2011;39(Web Server issue):W13-7. doi: 10.1093/nar/gkr245 21558174 PMC3125728

