# Supplementary material for: Effector loss and gain drives host range at a fitness cost
Source: PLoS Pathog. 2026 Aug 3;22(8):e1014145. doi: 10.1371/journal.ppat.1014145 (PMC13472402; doi:10.1371/journal.ppat.1014145)
Supplement: Supplementary file 1 [file ppat.1014145.s001.pdf]

Source Data for

Effector loss and gain drives host range at a fitness cost

Marcus V. Merfa *et al.*

*Corresponding authors. Email: mmerfaes@utk.edu; jacobs.1080@osu.edu

This PDF file includes:

Source Data for Comparative Genomic Analyses
Source Data for the Following Figures: 2, 4, S4, and S16

Source Data for Comparative Genomic Analyses

a. Comparison of the percentage of unique overall secreted proteins between Xtt and Xtu

Percentage of unique secreted proteins encoded by Xtt and Xtu genomes			
**Strain**	**percentage**	**transformed**	**Legend**
Xtt	1.74216028	0.1323772	Xtt – X. translucens pv. translucens strains
Xtt	1.58227848	0.12612276	Xtu – X. translucens pv. undulosa strains
Xtt	1.97183099	0.14088752	
Xtt	1.88679245	0.13779622	
Xtt	1.75438596	0.13284362	
Xtt	1.52439024	0.12378206	
Xtt	1.75953079	0.13303941	
Xtt	1.43678161	0.12015473	
Xtt	1.85185185	0.13650631	
Xtt	2.12765957	0.14638726	
Xtt	1.90735695	0.13854994	
Xtt	1.85758514	0.13671878	
Xtt	1.94552529	0.13993837	
Xtt	1.65562914	0.12902898	
Xtt	1.60427807	0.12700123	
Xtt	3.16205534	0.17877242	
Xtu	2.15517241	0.14733755	
Xtu	1.23839009	0.11151395	
Xtu	1.66666667	0.12946077	
Xtu	1.2012012	0.10981994	
Xtu	1.77935943	0.13379142	
Xtu	2.09205021	0.14514834	
Xtu	1.41342756	0.11916952	
Xtu	2.05761317	0.14394036	
Xtu	1.85873606	0.1367614	
Xtu	1.71232877	0.13123234	
Xtu	2.25225225	0.15064419	
Xtu	1.74825175	0.13260979	
Xtu	1.9379845	0.13966513	
Xtu	2.19298246	0.14863389	
Xtu	1.73611111	0.13214583	
Xtu	1.66666667	0.12946077	
Xtu	2.31481481	0.15273833	

Shapiro-Wilk normality test (data was submitted to a transformation that combines the arcsine and square root functions.)
data: transformed
W = 0.939, p-value = 0.0630

Two Sample t-test
data: transformed by Strain
t = 0.20543, df = 30.709, p-value = 0.7772

b. Comparison of the size of Type III effector (T3E) repertoires encoded by Xtt and Xtu strains

Number of T3Es encoded by Xtt and Xtu genomes		
**Strain**	**Number of predicted T3E identified in each genome via blastx**	**Legend**
Xtt	19	Xtt – X. translucens pv. translucens strains
Xtt	19	Xtu – X. translucens pv. undulosa strains
Xtt	19	
Xtt	19	
Xtt	19	
Xtt	19	
Xtt	19	
Xtt	18	
Xtt	19	
Xtt	18	
Xtt	18	
Xtt	19	
Xtt	19	
Xtt	18	
Xtu	13	
Xtu	13	
Xtu	13	
Xtu	13	
Xtu	14	
Xtu	13	
Xtu	13	
Xtu	14	
Xtu	14	
Xtu	14	
Xtu	15	
Xtu	15	
Xtu	13	
Xtu	14	

Shapiro-Wilk normality test
data: number of T3Es
W = 0.768, p-value = 0.000031

Wilcoxon rank-sum test
data: number of T3Es by Strain
W = 196, p-value = 3.436e-06

c. Comparison of the size of TAL effector (TALE) repertoires encoded by Xtt and Xtu strains

Number of TALEs encoded by Xtt and Xtu genomes		
**Strain**	**Number of predicted TALEs identified in each genome via AnnoTALE**	**Legend**
Xtt	4	Xtt – X. translucens pv. translucens strains
Xtt	5	Xtu – X. translucens pv. undulosa strains
Xtt	5	
Xtt	5	
Xtt	5	
Xtt	5	
Xtt	5	
Xtt	8	
Xtt	7	
Xtt	8	
Xtt	8	
Xtt	8	
Xtt	9	
Xtt	7	
Xtu	7	
Xtu	8	
Xtu	8	
Xtu	7	
Xtu	8	
Xtu	8	
Xtu	7	
Xtu	8	
Xtu	7	
Xtu	7	
Xtu	7	
Xtu	7	
Xtu	8	
Xtu	8	

Shapiro-Wilk normality test
data: number of T3Es
W = 0.94156, p-value = 0.1211

Two Sample t-test
data: transformed by Strain
t = -2.4779, df = 15.559, p-value = 0.02511

Source Data Fig. 2B

Percentage of symptomatic leaf area			
**Strain**	**percentage**	**transformed**	**Legend**
WT	24.38235782	0.516437001	WT – Xtt UPB820 WT
WT	19.49267703	0.457275447	mutant – Xtt UPB820 *xopAL1*
WT	30.76069325	0.58791011	
WT	23.399915	0.504916757	
WT	39.9602532	0.684313505	
WT	22.43050549	0.493383482	
WT	5.329905741	0.232967516	
WT	21.11612903	0.477457943	
WT	36.77276983	0.651532328	
WT	45.22124526	0.737537562	
WT	28.77282182	0.566169312	
WT	23.7746636	0.509330339	
WT	30.67156931	0.586944137	
WT	41.91277259	0.704169052	
WT	31.74624197	0.598541396	
WT	17.32125834	0.429249118	
WT	14.22040044	0.386662546	
WT	21.30642428	0.47978541	
WT	28.52741405	0.563455377	
WT	26.70488634	0.543071141	
WT	29.91739069	0.578738044	
WT	17.9709447	0.437770772	
WT	28.99985005	0.568673851	
WT	14.19899319	0.386355983	
WT	22.65934066	0.496121553	
WT	14.70736312	0.39358504	
WT	13.62907925	0.378122048	
WT	5.260285188	0.231413031	
WT	17.67137703	0.433856775	
WT	29.5586894	0.57481441	
WT	36.55471784	0.649269842	
WT	36.32093632	0.646840967	
WT	41.26062323	0.697553175	
WT	47.16235841	0.757006493	
WT	34.75434357	0.630474557	
WT	28.63622844	0.564659601	
WT	58.50652811	0.870879244	
WT	7.563677415	0.278611968	
WT	16.23909192	0.41476795	
WT	49.15894512	0.776987218	
WT	37.44238788	0.658462929	
WT	30.54280844	0.585547173	
WT	32.75624774	0.609345333	
WT	20.33259896	0.46779226	
WT	43.60026042	0.721224725	
WT	30.80843032	0.58842719	
WT	38.20715474	0.666348035	
WT	51.13611927	0.796760334	
mutant	21.55493852	0.482813567	
mutant	17.05593719	0.42573287	
mutant	18.89041096	0.449628518	
mutant	14.10215432	0.384966784	
mutant	10.96475864	0.337501699	
mutant	13.2317172	0.372295113	
mutant	22.00086931	0.488215756	
mutant	9.311994979	0.310101835	
mutant	16.36779184	0.41650998	
mutant	16.61255053	0.419807819	
mutant	18.48291302	0.444401494	
mutant	23.16589335	0.502148142	
mutant	14.48897654	0.390492496	
mutant	19.72000283	0.46013838	
mutant	15.40695798	0.403366607	
mutant	16.39666509	0.416900038	
mutant	9.322587672	0.310284044	
mutant	9.629861982	0.315529831	
mutant	9.477899733	0.312945048	
mutant	16.44736842	0.41758434	
mutant	7.205055097	0.27175489	
mutant	10.42567568	0.328779507	
mutant	9.528772426	0.313812411	
mutant	6.124161074	0.250068598	
mutant	5.780810919	0.242812087	
mutant	4.679762298	0.218051407	
mutant	17.9444852	0.437426099	
mutant	7.514665479	0.277683789	
mutant	15.34277849	0.402476974	
mutant	11.96514501	0.353204975	
mutant	11.53413089	0.346512173	
mutant	20.12218404	0.465173166	
mutant	6.721219751	0.262248837	
mutant	8.603238866	0.297690199	
mutant	10.78794977	0.334662217	
mutant	20.24587015	0.466713953	
mutant	5.073746313	0.227199396	
mutant	15.23219814	0.400940565	
mutant	28.08785374	0.558576688	
mutant	7.679398537	0.280792582	
mutant	14.74593417	0.394129258	
mutant	13.87485971	0.381690372	
mutant	21.37449904	0.480616175	
mutant	27.45849298	0.551550595	
mutant	19.22915207	0.453940735	
mutant	14.86465464	0.395800662	
mutant	10.10200724	0.323446842	

Shapiro-Wilk normality test (data was submitted to a transformation that combines the arcsine and square root functions.)
data: transformed
W = 0.975, p-value = 0.0671

Two Sample t-test
data: transformed by Strain
t = -7.3126, df = 93, p-value = 9.003e-11

Source Data Fig. 2C

Xtt and Xtu CFU populations in barley leaves inoculated via spray		
**Strain**	**CFU**	**Legend**
Xtt	7733333.333	Xtt – Xtt UPB820 WT
Xtt	25333333.33	dAL1 – Xtt UPB820 Δ*xopAL1*
Xtt	12000000	Xtu – Xtu UPB513 miniTn*7*::Gm
Xtt	22666666.67	XtuAL1 – Xtu UPB513 miniTn*7*::*xopAL1*
Xtt	10666666.67	
Xtt	36000000	
Xtt	24000000	
Xtt	3866666.667	
Xtt	54666666.67	
Xtt	38666666.67	
Xtt	18666666.67	
Xtt	13333333.33	
Xtt	16000000	
Xtt	13333333.33	
Xtt	140000000	
Xtt	24000000	
Xtt	44000000	
Xtt	34666666.67	
Xtt	30666666.67	
Xtt	22666666.67	
Xtt	11466666.67	
Xtt	30666666.67	
Xtt	13333333.33	
Xtt	12000000	
Xtt	56000000	
Xtt	22666666.67	
Xtt	1600000	
Xtt	320000	
Xtt	4666666.667	
Xtt	2800000	
Xtt	760000000	
Xtt	2400000	
Xtt	5333333.333	
Xtt	1253333.333	
Xtt	173333.3333	
Xtt	78666666.67	
Xtt	14666666.67	
Xtt	3600000	
Xtt	16000000	
Xtt	5866666.667	
Xtt	108000000	
Xtt	6666666.667	
Xtt	12533333.33	
Xtt	14666666.67	
Xtt	1333333.333	
Xtt	533333.3333	
Xtt	5066666.667	
dAL1	7600000	
dAL1	3333333.333	
dAL1	6000000	
dAL1	18666666.67	
dAL1	1866666.667	
dAL1	21333333.33	
dAL1	5733333.333	
dAL1	2133333.333	
dAL1	2266666.667	
dAL1	3733333.333	
dAL1	15733333.33	
dAL1	21333333.33	
dAL1	13333333.33	
dAL1	12000000	
dAL1	21333333.33	
dAL1	6933333.333	
dAL1	4266666.667	
dAL1	9600000	
dAL1	4400000	
dAL1	6000000	
dAL1	12000000	
dAL1	24000000	
dAL1	760000	
dAL1	15600000	
dAL1	373333.3333	
dAL1	533333.3333	
dAL1	5733333.333	
dAL1	4266666.667	
dAL1	4133333.333	
dAL1	3200000	
dAL1	2400000	
dAL1	12133333.33	
dAL1	7600000	
dAL1	13733333.33	
dAL1	18133333.33	
dAL1	14266666.67	
dAL1	16533333.33	
dAL1	13866666.67	
dAL1	2666666.667	
dAL1	3066666.667	
dAL1	7333333.333	
dAL1	5866666.667	
dAL1	3200000	
dAL1	9866666.667	
dAL1	18666666.67	
dAL1	5466666.667	
dAL1	733333.3333	
dAL1	13333333.33	
Xtu	4800000	
Xtu	1706666.667	

Xtu	2013333.333
Xtu	320000
Xtu	2533333.333
Xtu	41333.33333
Xtu	813333.3333
Xtu	3200000
Xtu	106666.6667
Xtu	893333.3333
Xtu	3333333.333
Xtu	141333.3333
Xtu	4666666.667
Xtu	6133333.333
Xtu	2186666.667
Xtu	3013333.333
Xtu	440000
Xtu	2933333.333
Xtu	960000
Xtu	2053.333333
Xtu	1200000
Xtu	5200000
Xtu	6000000
Xtu	1866.666667
Xtu	800000
Xtu	18533333.33
Xtu	853333.3333
Xtu	106666.6667
Xtu	173.3333333
Xtu	93333.33333
Xtu	1426666.667
Xtu	2533.333333
Xtu	52000
Xtu	546666.6667
Xtu	1200
Xtu	880000
Xtu	13333333.33
Xtu	2253333.333
Xtu	453.3333333
Xtu	533333.3333
Xtu	2493333.333
Xtu	1973333.333
Xtu	156000
Xtu	880000
Xtu	1946666.667
Xtu	7733333.333
Xtu	12000
Xtu	1933333.333
XtuAL1	5866666.667
XtuAL1	2666666.667
XtuAL1	4666666.667
XtuAL1	3200000
XtuAL1	4266666.667
XtuAL1	5466666.667
XtuAL1	28000
XtuAL1	2133333.333
XtuAL1	5333333.333
XtuAL1	5600000
XtuAL1	7733333.333
XtuAL1	3733333.333
XtuAL1	4000000
XtuAL1	1733333.333
XtuAL1	76000
XtuAL1	2000000
XtuAL1	2666666.667
XtuAL1	7333333.333
XtuAL1	520000
XtuAL1	6266666.667
XtuAL1	7866666.667
XtuAL1	933333.3333
XtuAL1	453333.3333
XtuAL1	4933333.333
XtuAL1	16533333.33
XtuAL1	813333.3333
XtuAL1	8800000
XtuAL1	3866666.667
XtuAL1	13600000
XtuAL1	1800000
XtuAL1	226666.6667
XtuAL1	10000
XtuAL1	3186666.667
XtuAL1	8400000
XtuAL1	266666.6667
XtuAL1	7733333.333
XtuAL1	214666666.7
XtuAL1	4000000
XtuAL1	5333333.333
XtuAL1	2026666.667
XtuAL1	760000
XtuAL1	20400000
XtuAL1	15466666.67
XtuAL1	1186666.667
XtuAL1	813333.3333
XtuAL1	480000
XtuAL1	22400000

Shapiro-Wilk normality test

data: CFU

W = 0.90956, p-value = 2.19e-09

Kruskal-Wallis rank sum test
data: CFU by Strain
Kruskal-Wallis chi-squared = 62.965, df = 3, p-value = 1.366e-13

Wilcoxon rank-sum test

y	group1	group2	p	p.adj	p.adj.signif
CFU	dAL1	Xtt	6.00E-03	6.00E-03	**
CFU	dAL1	Xtu	2.61E-09	1.30E-08	****
CFU	dAL1	XtuAL1	3.00E-03	6.00E-03	**
CFU	Xtt	Xtu	7.70E-11	4.62E-10	****
CFU	Xtt	XtuAL1	6.45E-06	2.58E-05	****
CFU	Xtu	XtuAL1	3.90E-04	1.00E-03	***

Source Data Fig. 4D

Chlorophyl concentration per inoculated wheat leaf		
**Strain**	**Concentration**	**Legend**
Mock	58.36666667	Mock – inoculation with sterile water
Mock	62.46666667	Xtt – Xtt UPB820 WT
Mock	86.56666667	dAL1 – Xtt UPB820 Δ*xopAL1*
Mock	96.33333333	Xtu – Xtu UPB513 WT
Mock	161.7666667	XtuAL1 – Xtu UPB513 miniTn*7*::*xopAL1*
Mock	149.5333333	
Mock	234.0666667	
Mock	182.9666667	
Mock	143.9333333	
Mock	174.2	
Mock	109	
Mock	212	
Mock	100.8666667	
Mock	186.2666667	
Mock	120.9	
Mock	130.7	
Xtt	70.2	
Xtt	81.66666667	
Xtt	68.6	
Xtt	73.06666667	
Xtt	71.46666667	
Xtt	55.5	
Xtt	90.63333333	
Xtt	68.16666667	
Xtt	108.2	
Xtt	81.66666667	
Xtt	80.83333333	
Xtt	77.2	
Xtt	78.43333333	
Xtt	103.7	
Xtt	96.4	
Xtt	66.13333333	
dAL1	63.7	
dAL1	73.06666667	
dAL1	106.2	
dAL1	94.7	
dAL1	93.1	
dAL1	175.6333333	
dAL1	119.2666667	
dAL1	110.2	
dAL1	151.1333333	
dAL1	123.3333333	
dAL1	87	
dAL1	110.2333333	
dAL1	126.6	
dAL1	131.9333333	
Xtu	101.2666667	
Xtu	75.53333333	
Xtu	88.6	
Xtu	112.2333333	
Xtu	73.06666667	
Xtu	120.0333333	
Xtu	115.2	
Xtu	109.4333333	
Xtu	116.8	
Xtu	124.1666667	
Xtu	80	
Xtu	124.1666667	
Xtu	113.5333333	
Xtu	75.96666667	
XtuAL1	74.33333333	
XtuAL1	60.43333333	
XtuAL1	100.4666667	
XtuAL1	64.53333333	
XtuAL1	86.56666667	
XtuAL1	93.5	
XtuAL1	109.8333333	
XtuAL1	86.13333333	
XtuAL1	80.83333333	
XtuAL1	71.06666667	
XtuAL1	86.16666667	
XtuAL1	85.76666667	
XtuAL1	72.7	
XtuAL1	56.33333333	

Shapiro-Wilk normality test

data: Concentration

W = 0.886, p-value = 0.00000663

Kruskal-Wallis rank sum test

data: Concentration by Strain

Kruskal-Wallis chi-squared = 25.767, df = 4, p-value = 3.526e-05

Wilcoxon rank-sum test

y	group1	group2	p	p.adj	p.adj.signif
Concentration	dAL1	Mock	0.193	0.579	ns
Concentration	dAL1	Xtt	0.002	0.011	*
Concentration	dAL1	Xtu	0.491	0.982	ns
Concentration	dAL1	XtuAL1	0.001	0.009	**
Concentration	Mock	Xtt	0.000851	0.009	**
Concentration	Mock	Xtu	0.059	0.234	ns
Concentration	Mock	XtuAL1	0.001	0.009	**
Concentration	Xtt	Xtu	0.003	0.019	*
Concentration	Xtt	XtuAL1	0.708	0.982	ns
Concentration	Xtu	XtuAL1	0.006	0.031	*

Source Data for Supplementary Fig. S4A

**Xtt and Xtu CFU populations in wheat leaves inoculated via infiltration**				
**Strain**	**CFU**	**Log10(CFU)**	**Time**	**Legend**
Xtt	168	2.225309282	2h	Xtt – Xtt UPB820 WT
Xtt	128	2.10720997	2h	dAL1 – Xtt UPB820 Δ*xopAL1*
Xtt	69.33333333	1.84094208	2h	comp – Xtt UPB820 Δ*xopAL1*/*xopAL1*
dAL1	80	1.903089987	2h	Xtu – Xtu UPB513 miniTn*7*::Gm
dAL1	96	1.982271233	2h	XtuAL1 – Xtu UPB513 miniTn*7*::*xopAL1*
dAL1	156	2.193124598	2h	
comp	112	2.049218023	2h	
comp	117.3333333	2.069421409	2h	
comp	120	2.079181246	2h	
Xtu	93.33333333	1.970036777	2h	
Xtu	64	1.806179974	2h	
Xtu	40	1.602059991	2h	
XtuAL1	16	1.204119983	2h	
XtuAL1	8	0.903089987	2h	
XtuAL1	24	1.380211242	2h	
Xtt	9066.666667	3.957447649	24h	
Xtt	10666.66667	4.028028724	24h	
Xtt	13866.67	4.14197218	24h	
dAL1	93333.33333	4.970036777	24h	
dAL1	74666.66667	4.873126764	24h	
dAL1	88000	4.944482672	24h	
comp	19466.66667	4.289291592	24h	
comp	10933.33333	4.038752589	24h	
comp	7200	3.857332496	24h	
Xtu	27466.67	4.43880601	24h	
Xtu	26666.66667	4.425968732	24h	
Xtu	42133.33333	4.624625819	24h	
XtuAL1	25600	4.408239965	24h	
XtuAL1	21066.66667	4.323595824	24h	
XtuAL1	26133.33333	4.417194808	24h	
Xtt	93333.33333	4.970036777	72h	
Xtt	96666.66667	4.985276743	72h	
Xtt	116666.6667	5.06694679	72h	
dAL1	27333333.33	7.436692598	72h	
dAL1	13666666.67	7.135662602	72h	
dAL1	23000000	7.361727836	72h	
comp	50000	4.698970004	72h	
comp	96666.66667	4.985276743	72h	
comp	116666.6667	5.06694679	72h	
Xtu	19333333.33	7.286306739	72h	
Xtu	24000000	7.380211242	72h	
Xtu	22666666.67	7.355387658	72h	
XtuAL1	303333.3333	5.481920138	72h	
XtuAL1	453333.3333	5.656417654	72h	
XtuAL1	363333.3333	5.560305243	72h	
Xtt	353333.3333	5.548184611	120h	
Xtt	436666.6667	5.640150041	120h	
Xtt	126666.6667	5.102662342	120h	
dAL1	33333333.33	7.522878745	120h	
dAL1	103333333.3	8.014240439	120h	
dAL1	70000000	7.84509804	120h	
comp	250000	5.397940009	120h	
comp	323333.3333	5.50965048	120h	
comp	256666.6667	5.409369471	120h	
Xtu	170000000	8.230448921	120h	
Xtu	240000000	8.380211242	120h	
Xtu	170000000	8.230448921	120h	
XtuAL1	19000000	7.278753601	120h	
XtuAL1	2833333.333	6.452297671	120h	
XtuAL1	2200000	6.342422681	120h	
Xtt	1146666.667	6.059437188	168h	
Xtt	773333.3333	5.88836673	168h	
Xtt	666666.6667	5.823908741	168h	
dAL1	32800000	7.515873844	168h	
dAL1	23733333.33	7.375358739	168h	
dAL1	39466666.67	7.596230448	168h	
comp	1120000	6.049218023	168h	
comp	1120000	6.049218023	168h	
comp	480000	5.681241237	168h	
Xtu	232000000	8.365487985	168h	
Xtu	157333333.3	8.196820744	168h	
Xtu	109333333.3	8.038752589	168h	
XtuAL1	5866666.667	6.768391413	168h	
XtuAL1	4533333.333	6.656417654	168h	
XtuAL1	12000000	7.079181246	168h	
Xtt	154.6666667	2.189396726	2h	
Xtt	154.6666667	2.189396726	2h	
Xtt	138.6666667	2.141972076	2h	
dAL1	205.3333333	2.312459457	2h	
dAL1	248	2.394451681	2h	
dAL1	285.3333333	2.45535251	2h	
comp	192	2.283301229	2h	
comp	130.6666667	2.116164812	2h	
comp	133.3333333	2.124938736	2h	
Xtu	192	2.283301229	2h	
Xtu	213.3333333	2.329058719	2h	
Xtu	146.6666667	2.166331422	2h	
XtuAL1	168	2.225309282	2h	
XtuAL1	128	2.10720997	2h	
XtuAL1	189.3333333	2.277227081	2h	
Xtt	4693.333333	3.6714814	24h	
Xtt	4480	3.651278014	24h	
Xtt	4640	3.666517981	24h	
dAL1	666.6666667	2.823908741	24h	
dAL1	1146.666667	3.059437188	24h	
dAL1	2240	3.350248018	24h	
comp	2960	3.471291711	24h	

comp	2640	3.421603927	24h
comp	4453.333333	3.648685203	24h
Xtu	1333.333333	3.124938736	24h
Xtu	906.6666667	2.957447649	24h
Xtu	3893.333333	3.590321588	24h
XtuAL1	960	2.982271233	24h
XtuAL1	1920	3.283301229	24h
XtuAL1	1600	3.204119983	24h
Xtt	154666.6667	5.189396726	72h
Xtt	202666.6667	5.306782325	72h
Xtt	104000	5.017033339	72h
dAL1	2160000	6.334453751	72h
dAL1	2453333.333	6.38975656	72h
dAL1	3306666.667	6.519390417	72h
comp	165333.3333	5.218360422	72h
comp	128000	5.10720997	72h
comp	178666.6667	5.252043535	72h
Xtu	7733333.333	6.88836673	72h
Xtu	8266666.667	6.917330426	72h
Xtu	9333333.333	6.970036777	72h
XtuAL1	218666.6667	5.339782585	72h
XtuAL1	77333.33333	4.88836673	72h
XtuAL1	136000	5.133538908	72h
Xtt	269333.3333	5.430290106	120h
Xtt	250666.6667	5.399096586	120h
Xtt	242666.6667	5.385010125	120h
dAL1	11466666.67	7.059437188	120h
dAL1	29866666.67	7.475186755	120h
dAL1	12266666.67	7.088726564	120h
comp	362666.6667	5.559507641	120h
comp	280000	5.447158031	120h
comp	197333.3333	5.295200452	120h
Xtu	37333333.33	7.572096768	120h
Xtu	77333333.33	7.88836673	120h
Xtu	29333333.33	7.467361417	120h
XtuAL1	162666.6667	5.211298567	120h
XtuAL1	720000	5.857332496	120h
XtuAL1	560000	5.748188027	120h
Xtt	640000	5.806179974	168h
Xtt	826666.6667	5.917330426	168h
Xtt	960000	5.982271233	168h
dAL1	21866666.67	7.339782585	168h
dAL1	10933333.33	7.038752589	168h
dAL1	12266666.67	7.088726564	168h
comp	1040000	6.017033339	168h
comp	2586666.667	6.412740467	168h
comp	1173333.333	6.069421409	168h
Xtu	210666666.7	8.323595824	168h
Xtu	133333333.3	8.124938736	168h
Xtu	117333333.3	8.069421409	168h
XtuAL1	506666.6667	5.704722333	168h
XtuAL1	853333.3333	5.931118711	168h
XtuAL1	1200000	6.079181246	168h

- Analysis at 2h post inoculation

Shapiro-Wilk normality test
data: Log10(CFU)
W = 0.97679, p-value = 0.7354

Tukey multiple comparisons of means

**y**	**Groups**	**p adj**
Log10(CFU)	Xtu-XtuAL1	0.3630725
Log10(CFU)	Xtt-XtuAL1	0.1636946
Log10(CFU)	comp-XtuAL1	0.156258
Log10(CFU)	dAL1-XtuAL1	0.06146
Log10(CFU)	Xtt-Xtu	0.9880129
Log10(CFU)	comp-Xtu	0.9855048
Log10(CFU)	dAL1-Xtu	0.8619679
Log10(CFU)	comp-Xtt	0.9999999
Log10(CFU)	dAL1-Xtt	0.9872196
Log10(CFU)	dAL1-comp	0.9895107

- Analysis at 24h post inoculation

Shapiro-Wilk normality test
data: Log10(CFU)
W = 0.9598, p-value = 0.3062

Tukey multiple comparisons of means

y	Groups	p adj
Log10(CFU)	comp-XtuAL1	0.9999988
Log10(CFU)	Xtt-XtuAL1	0.9994802
Log10(CFU)	Xtu-XtuAL1	0.9992667
Log10(CFU)	dAL1-XtuAL1	0.9724273
Log10(CFU)	Xtt-comp	0.999803
Log10(CFU)	Xtu-comp	0.9996952
Log10(CFU)	dAL1-comp	0.9794256
Log10(CFU)	Xtu-Xtt	1
Log10(CFU)	dAL1-Xtt	0.9946584
Log10(CFU)	dAL1-Xtu	0.9956174

- Analysis at 72h post inoculation

Shapiro-Wilk normality test
data: Log10(CFU)
W = 0.97921, p-value = 0.8041

Tukey multiple comparisons of means

y	Groups	p adj
Log10(CFU)	Xtt-comp	0.9996234
Log10(CFU)	XtuAL1-comp	0.470382
Log10(CFU)	dAL1-comp	0
Log10(CFU)	Xtu-comp	0
Log10(CFU)	XtuAL1-Xtt	0.5908261
Log10(CFU)	dAL1-Xtt	0
Log10(CFU)	Xtu-Xtt	0
Log10(CFU)	dAL1-XtuAL1	0
Log10(CFU)	Xtu-XtuAL1	0
Log10(CFU)	Xtu-dAL1	0.5347326

- Analysis at 120h post inoculation

Shapiro-Wilk normality test
data: Log10(CFU)
W = 0.95366, p-value = 0.2117

Tukey multiple comparisons of means

y	Groups	p adj
Log10(CFU)	comp-Xtt	0.9999901
Log10(CFU)	XtuAL1-Xtt	0.0361309
Log10(CFU)	dAL1-Xtt	0
Log10(CFU)	Xtu-Xtt	0
Log10(CFU)	XtuAL1-comp	0.0431103
Log10(CFU)	dAL1-comp	0
Log10(CFU)	Xtu-comp	0
Log10(CFU)	dAL1-XtuAL1	0.0000552
Log10(CFU)	Xtu-XtuAL1	0.0000005
Log10(CFU)	Xtu-dAL1	0.321483

- Analysis at 168h post inoculation

Shapiro-Wilk normality test

data: Log10(CFU)

W = 0.97622, p-value = 0.7187

Tukey multiple comparisons of means

y	Groups	p adj
Log10(CFU)	comp-Xtt	0.929786
Log10(CFU)	XtuAL1-Xtt	0.0794022
Log10(CFU)	dAL1-Xtt	0.0000001
Log10(CFU)	Xtu-Xtt	0
Log10(CFU)	XtuAL1-comp	0.3314649
Log10(CFU)	dAL1-comp	0.0000005
Log10(CFU)	Xtu-comp	0
Log10(CFU)	dAL1-XtuAL1	0.0000583
Log10(CFU)	Xtu-XtuAL1	0
Log10(CFU)	Xtu-dAL1	0.0002446

Source Data for Supplementary Fig. S4B

Xtt and Xtu CFU populations in barley leaves inoculated via infiltration				
**Strain**	**CFU**	**Log10(CFU)**	**Time**	**Legend**
Xtt	112	2.04921802	2h	Xtt – Xtt UPB820 WT
Xtt	157.333333	2.19682074	2h	dAL1 – Xtt UPB820 Δ*xopAL1*
Xtt	109.333333	2.03875259	2h	comp – Xtt UPB820 Δ*xopAL1*/*xopAL1*
dAL1	133.333333	2.12493874	2h	Xtu – Xtu UPB513 miniTn*7*::Gm
dAL1	96	1.98227123	2h	XtuAL1 – Xtu UPB513 miniTn*7*::*xopAL1*
dAL1	128	2.10720997	2h	
comp	178.666667	2.25204354	2h	
comp	82.6666667	1.91733043	2h	
comp	181.333333	2.25847764	2h	
Xtu	189.333333	2.27722708	2h	
Xtu	104	2.01703334	2h	
Xtu	194.666667	2.28929159	2h	
XtuAL1	34.6666667	1.53991208	2h	
XtuAL1	66.6666667	1.82390874	2h	
XtuAL1	101.333333	2.00575233	2h	
Xtt	14400	4.15836249	24h	
Xtt	9866.66667	3.99417046	24h	
Xtt	1520	3.18184359	24h	
dAL1	4533.33333	3.65641765	24h	
dAL1	8266.66667	3.91733043	24h	
dAL1	4266.66667	3.63008871	24h	
comp	2400	3.38021124	24h	
comp	8000	3.90308999	24h	
comp	266.666667	2.42596873	24h	
Xtu	480	2.68124124	24h	
Xtu	4800	3.68124124	24h	
Xtu	9600	3.98227123	24h	
XtuAL1	4266.66667	3.63008871	24h	
XtuAL1	3200	3.50514998	24h	
XtuAL1	3200	3.50514998	24h	
Xtt	18933333.3	7.27722708	72h	
Xtt	10666666.7	7.02802872	72h	
Xtt	15733333.3	7.19682074	72h	
dAL1	5866666.67	6.76839141	72h	
dAL1	13600000	7.13353891	72h	
dAL1	7733333.33	6.88836673	72h	
comp	13333333.3	7.12493874	72h	
comp	10400000	7.01703334	72h	
comp	25066666.7	7.39909659	72h	
Xtu	2986666.67	6.47518675	72h	
Xtu	986666.667	5.99417046	72h	
Xtu	640000	5.80617997	72h	
XtuAL1	1200000	6.07918125	72h	
XtuAL1	2800000	6.44715803	72h	
XtuAL1	1546666.67	6.18939673	72h	
Xtt	37333333.3	7.57209677	120h	
Xtt	106666667	8.02802872	120h	
Xtt	66666666.7	7.82390874	120h	
dAL1	37333333.3	7.57209677	120h	
dAL1	53333333.3	7.72699873	120h	
dAL1	50666666.7	7.70472233	120h	
comp	48000000	7.68124124	120h	
comp	61333333.3	7.78769657	120h	
comp	85333333.3	7.93111871	120h	
Xtu	10666666.7	7.02802872	120h	
Xtu	12266666.7	7.08872656	120h	
Xtu	4533333.33	6.65641765	120h	
XtuAL1	29600000	7.47129171	120h	
XtuAL1	36266666.7	7.55950764	120h	
XtuAL1	3466666.67	6.53991208	120h	
Xtt	546666667	8.73772259	168h	
Xtt	69333333.3	7.84094208	168h	
Xtt	98666666.7	7.99417046	168h	
dAL1	109333333	8.03875259	168h	
dAL1	26666666.7	7.42596873	168h	
dAL1	133333333	8.12493874	168h	
comp	61333333.3	7.78769657	168h	
comp	154666667	8.18939673	168h	
comp	56000000	7.74818803	168h	
Xtu	3466666.67	6.53991208	168h	
Xtu	2400000	6.38021124	168h	
Xtu	85333333.3	7.93111871	168h	
XtuAL1	40000000	7.60205999	168h	
XtuAL1	48000000	7.68124124	168h	
XtuAL1	18400000	7.26481782	168h	
Xtt	160	2.20411998	2h	
Xtt	109.333333	2.03875259	2h	
Xtt	205.333333	2.31245946	2h	
dAL1	152	2.18184359	2h	
dAL1	138.666667	2.14197208	2h	
dAL1	45.3333333	1.65641765	2h	
comp	130.666667	2.11616481	2h	
comp	218.666667	2.33978258	2h	
comp	77.3333333	1.88836673	2h	
Xtu	186.666667	2.27106677	2h	
Xtu	277.333333	2.44300207	2h	
Xtu	157.333333	2.19682074	2h	
XtuAL1	104	2.01703334	2h	
XtuAL1	170.666667	2.23214871	2h	
XtuAL1	120	2.07918125	2h	
Xtt	74666.6667	4.87312676	24h	
Xtt	34666.6667	4.53991208	24h	
Xtt	29333.33	4.46736137	24h	
dAL1	29333.3333	4.46736142	24h	
dAL1	32000	4.50514998	24h	
dAL1	16000	4.20411998	24h	
comp	210666.667	5.32359582	24h	

comp	61333.3333	4.78769657	24h
comp	50666.6667	4.70472233	24h
Xtu	221333.33	5.34504682	24h
Xtu	80000	4.90308999	24h
Xtu	80000	4.90308999	24h
XtuAL1	154666.667	5.18939673	24h
XtuAL1	216000	5.33445375	24h
XtuAL1	80000	4.90308999	24h
Xtt	37666666.7	7.57595719	72h
Xtt	46333333.3	7.66589355	72h
Xtt	19666666.7	7.29373076	72h
dAL1	23333333.3	7.36797679	72h
dAL1	19000000	7.2787536	72h
dAL1	19666666.7	7.29373076	72h
comp	17666666.7	7.24715461	72h
comp	13666666.7	7.1356626	72h
comp	17333333.3	7.23888209	72h
Xtu	22000000	7.34242268	72h
Xtu	18000000	7.25527251	72h
Xtu	18000000	7.25527251	72h
XtuAL1	14666666.7	7.16633142	72h
XtuAL1	4000000	6.60205999	72h
XtuAL1	7000000	6.84509804	72h
Xtt	63333333.3	7.80163235	120h
Xtt	346666667	8.53991208	120h
Xtt	80000000	7.90308999	120h
dAL1	86666666.7	7.93785209	120h
dAL1	116666667	8.06694679	120h
dAL1	173333333	8.23888209	120h
comp	70000000	7.84509804	120h
comp	66666666.7	7.82390874	120h
comp	86666666.7	7.93785209	120h
Xtu	25000000	7.39794001	120h
Xtu	19666666.7	7.29373076	120h
Xtu	21333333.3	7.32905872	120h
XtuAL1	15666666.7	7.1949766	120h
XtuAL1	29000000	7.462398	120h
XtuAL1	36666666.7	7.56427143	120h
Xtt	56000000	7.74818803	168h
Xtt	90666666.7	7.95744765	168h
Xtt	117333333	8.06942141	168h
dAL1	80000000	7.90308999	168h
dAL1	117333333	8.06942141	168h
dAL1	57066666.7	7.75638251	168h
comp	176000000	8.24551267	168h
comp	232000000	8.36548798	168h
comp	493333333	8.69314046	168h
Xtu	80000000	7.90308999	168h
Xtu	50666666.7	7.70472233	168h
Xtu	101333333	8.00575233	168h
XtuAL1	101333333	8.00575233	168h
XtuAL1	104000000	8.01703334	168h
XtuAL1	136000000	8.13353891	168h

- Analysis at 2h post inoculation

Shapiro-Wilk normality test
data: Log10(CFU)
W = 0.94328, p-value = 0.1115

Tukey multiple comparisons of means

y	Groups	p adj
Log10(CFU)	dAL1-XtuAL1	0.93096150
Log10(CFU)	comp-XtuAL1	0.44636651
Log10(CFU)	Xtt-XtuAL1	0.38606750
Log10(CFU)	Xtu-XtuAL1	0.05907348
Log10(CFU)	comp-dAL1	0.88681572
Log10(CFU)	Xtt-dAL1	0.84037090
Log10(CFU)	Xtu-dAL1	0.26450772
Log10(CFU)	Xtt-comp	0.99996673
Log10(CFU)	Xtu-comp	0.77895321
Log10(CFU)	Xtu-Xtt	0.83374561

- Analysis at 24h post inoculation

Shapiro-Wilk normality test
data: Log10(CFU)
W = 0.96293, p-value = 0.3671

Tukey multiple comparisons of means

y	Groups	p adj
Log10(CFU)	comp-dAL1	0.9999984
Log10(CFU)	Xtt-dAL1	0.9982997
Log10(CFU)	Xtu-dAL1	0.9947573
Log10(CFU)	XtuAL1-dAL1	0.9752660
Log10(CFU)	Xtt-comp	0.9991953
Log10(CFU)	Xtu-comp	0.9969342
Log10(CFU)	XtuAL1-comp	0.9822050
Log10(CFU)	Xtu-Xtt	0.9999771
Log10(CFU)	XtuAL1-Xtt	0.9981498
Log10(CFU)	XtuAL1-Xtu	0.9996168

- Analysis at 72h post inoculation

Shapiro-Wilk normality test
data: Log10(CFU)
W = 0.97717, p-value = 0.7464

Tukey multiple comparisons of means

y	Groups	p adj
Log10(CFU)	Xtu-XtuAL1	0.97592968
Log10(CFU)	dAL1-XtuAL1	0.12406800
Log10(CFU)	comp-XtuAL1	0.06542027
Log10(CFU)	Xtt-XtuAL1	0.01552039
Log10(CFU)	dAL1-Xtu	0.33910553
Log10(CFU)	comp-Xtu	0.20344927
Log10(CFU)	Xtt-Xtu	0.05816480
Log10(CFU)	comp-dAL1	0.99766528
Log10(CFU)	Xtt-dAL1	0.87093034
Log10(CFU)	Xtt-comp	0.96663824

- Analysis at 120h post inoculation

Shapiro-Wilk normality test
data: Log10(CFU)
W = 0.96498, p-value = 0.4124

Tukey multiple comparisons of means

y	Groups	p adj
Log10(CFU)	XtuAL1-Xtu	0.8504447667
Log10(CFU)	comp-Xtu	0.0022574388
Log10(CFU)	dAL1-Xtu	0.0012272535
Log10(CFU)	Xtt-Xtu	0.0004188665
Log10(CFU)	comp-XtuAL1	0.0256224325
Log10(CFU)	dAL1-XtuAL1	0.0145837587
Log10(CFU)	Xtt-XtuAL1	0.0052388971
Log10(CFU)	dAL1-comp	0.9991836284
Log10(CFU)	Xtt-comp	0.9618170747
Log10(CFU)	Xtt-dAL1	0.9928121267

- Analysis at 168h post inoculation

Shapiro-Wilk normality test
data: Log10(CFU)
W = 0.96774, p-value = 0.4794

Tukey multiple comparisons of means

y	Groups	p adj
Log10(CFU)	XtuAL1-Xtu	0.59786023
Log10(CFU)	dAL1-Xtu	0.36502679
Log10(CFU)	Xtt-Xtu	0.11632521
Log10(CFU)	comp-Xtu	0.04640667
Log10(CFU)	dAL1-XtuAL1	0.99426537
Log10(CFU)	Xtt-XtuAL1	0.82003184
Log10(CFU)	comp-XtuAL1	0.56375783
Log10(CFU)	Xtt-dAL1	0.96106348
Log10(CFU)	comp-dAL1	0.79787177
Log10(CFU)	comp-Xtt	0.99146989

Source Data for Supplementary Fig. S16B

**Percentage of symptomatic leaf area**			
**Strain**	**percentage**	**transformed**	**Legend**
WT	72.44582043	1.018174323	WT – Xtt UPB820 WT
WT	76.99400645	1.070545511	AJ – Xtt UPB820 Δ*xopAJ*
WT	16.38521223	0.416745351	
WT	76.88834154	1.069291206	
WT	78.30018083	1.086223229	
WT	88.12615955	1.219000298	
WT	58.4701815	0.870510424	
WT	34.09594096	0.623545732	
WT	43.0733945	0.715908624	
WT	9.776536313	0.318007444	
WT	78.83834281	1.092780842	
WT	23.11740891	0.501573324	
WT	67.30959446	0.962152632	
WT	59.2472119	0.878405724	
WT	15.08274232	0.398856727	
WT	13.22654462	0.372218778	
WT	56	0.845543105	
WT	62.7154047	0.913964268	
WT	31.51540383	0.596059447	
WT	71.31661442	1.005615275	
WT	67.42857143	0.963421417	
WT	50.25614754	0.78795965	
WT	58.1443299	0.867205974	
WT	57.61947701	0.861890965	
WT	95.05395683	1.346523957	
WT	46.62331166	0.75160556	
WT	42.68402154	0.71197478	
WT	77.5147929	1.076757429	
WT	70.97050021	1.001795814	
WT	46.78942398	0.7532703	
WT	54.67422096	0.832208725	
WT	67.41793239	0.963307912	
WT	59.43738657	0.880341564	
WT	86.99331849	1.201834016	
WT	60.34261242	0.889576425	
WT	77.07832773	1.071547903	
WT	28.41901067	0.562254329	
WT	54.75578406	0.833028007	
WT	61.79372197	0.904457313	
WT	57.03301673	0.855962338	
WT	67.76395022	0.967004427	
WT	42.73560209	0.712496157	
WT	88.77697842	1.229182835	
WT	96.61921708	1.38587515	
WT	81.03186646	1.120175794	
WT	48.94351013	0.774832478	
WT	64.77654974	0.93540381	
WT	55.52511416	0.84076237	
AJ	55.75379126	0.843063829	
AJ	24.31681334	0.51567342	
AJ	28.73980054	0.565804538	
AJ	38.45843422	0.66893217	
AJ	24.61097461	0.519094925	
AJ	1.686507937	0.130233451	
AJ	16.04696673	0.412157027	
AJ	7.817303469	0.283371647	
AJ	19.3767479	0.455810573	
AJ	8.981788079	0.304374321	
AJ	28.979793	0.56845282	
AJ	59.4012605	0.879973717	
AJ	51.09020949	0.796301122	
AJ	33.82633588	0.620699253	
AJ	5.457301668	0.235787412	
AJ	10.87848933	0.336118778	
AJ	4.763894693	0.220034694	
AJ	23.40173639	0.504938267	
AJ	1.53441473	0.124190486	
AJ	39.71161341	0.681774082	
AJ	25.35670607	0.52770796	
AJ	20.29250457	0.467293976	
AJ	8.785046729	0.300916654	
AJ	7.828518173	0.283580462	
AJ	90.93725794	1.265009196	
AJ	84.71705907	1.169150173	
AJ	43.00314465	0.715199215	
AJ	71.63212435	1.009109024	
AJ	51.45283862	0.799928595	
AJ	6.228201295	0.252229616	
AJ	63.23387873	0.919332942	
AJ	28.04757185	0.558128444	
AJ	44.45422535	0.729826074	
AJ	59.60949464	0.88209474	
AJ	85.81187599	1.184596069	
AJ	27.85299807	0.555960512	
AJ	50.45417011	0.789939927	
AJ	15.30612245	0.401968177	
AJ	57.10158883	0.856655013	
AJ	43.77423524	0.722978509	
AJ	55.10455105	0.836532763	
AJ	88.03216651	1.217549936	
AJ	69.67953985	0.987665371	
AJ	53.08641975	0.816281995	
AJ	43.7994723	0.72323285	
AJ	16.9599218	0.424455057	
AJ	24.8372093	0.521716982	
AJ	77.98951882	1.082464565	

Shapiro-Wilk normality test (data was submitted to a transformation that combines the arcsine and square root functions.)
data: transformed
W = 0.982, p-value = 0.223

Two Sample t-test
data: transformed by Strain
t = -4.3029, df = 94, p-value = 4.126e-05

Source Data for Supplementary Fig. S16C

Xtt CFU populations in barley leaves inoculated via spray		
**Strain**	**CFU**	**Legend**
WT	85333333.33	WT – Xtt UPB820 WT
WT	37333333.33	AJ – Xtt UPB820 Δ*xopAJ*
WT	10133333.33	
WT	162666666.7	
WT	12266666.67	
WT	74666666.67	
WT	5066666.667	
WT	2053333333	
WT	12533333.33	
WT	45333333.33	
WT	218666666.7	
WT	370666666.7	
WT	2320000	
WT	3466666.667	
WT	557333333.3	
WT	74666666.67	
WT	77333333.33	
WT	109333333.3	
WT	4320000000	
WT	3413333333	
WT	3040000000	
WT	69333333.33	
WT	400000000	
WT	346666666.7	
WT	8266666.667	
WT	14133333.33	
WT	1440000	
WT	266666.6667	
WT	613333.3333	
WT	1173333.333	
WT	320000	
WT	13333333.33	
WT	24000000	
WT	2186666.667	
WT	3200000	
WT	3733333.333	
WT	6666666.667	
WT	373333.3333	
WT	17066666.67	
WT	26666666.67	
WT	1600000	
WT	162666.6667	
WT	5866666.667	
WT	5866666.667	
WT	8266666.667	
WT	4800000	
WT	8266666.667	
WT	266666.6667	
AJ	69333333.33	
AJ	7786666667	
AJ	520000000	
AJ	512000000	
AJ	805333333.3	
AJ	93333333.33	
AJ	197333333.3	
AJ	2293333.333	
AJ	309333333.3	
AJ	362666666.7	
AJ	18666666.67	
AJ	8266666.667	
AJ	224000	
AJ	442666666.7	
AJ	1066666.667	
AJ	28800000	
AJ	11200000	
AJ	280000000	
AJ	1866666.667	
AJ	14666666.67	
AJ	56800000	
AJ	36533333.33	
AJ	2453333.333	
AJ	213333.3333	
AJ	85333333.33	
AJ	290666.6667	
AJ	5866666.667	
AJ	176000000	
AJ	16533333.33	
AJ	117333333.3	
AJ	14133333.33	
AJ	82666666.67	
AJ	29333333.33	
AJ	560000	
AJ	36266666.67	
AJ	15733333.33	
AJ	20800000	
AJ	9066666.667	
AJ	3733333.333	
AJ	34666666.67	
AJ	9333333.333	
AJ	30400000	
AJ	35733333.33	
AJ	4000000	
AJ	8533333.333	
AJ	22133333.33	
AJ	200000000	
AJ	178666666.7	

Shapiro-Wilk normality test
data: CFU
W = 0.98531, p-value = 0.3623

Tukey multiple comparisons of means

y	group1	group2	p.adj
CFU	WT	AJ	0.7686251
