# Supplementary material for: Effector loss and gain drives host range at a fitness cost
Source: PLoS Pathog. 2026 Aug 3;22(8):e1014145. doi: 10.1371/journal.ppat.1014145 (PMC13472402; doi:10.1371/journal.ppat.1014145)
Supplement: Supplementary file 2 [file ppat.1014145.s004.pdf]

S3 Table. Symptom development assessment in a cereal genotype diversity panel inoculated with Xtt and Xtu strains.

				**Xanthomonas translucens strain**				
				**Xtt UPB820 WT**	**Xtt UPB820 Δ*xopAL1***	**Xtt UPB820 Δ*xopAL1*/Δ*xopAL1***	**Xtu UPB513 miniTn7::Gm**	**Xtu UPB513 miniTn7::*xopAL1***
**Host**	**Scientific name**	**Genotype**	**Cultivar/variety/ genotype/line**	**Symptom**				
Wheat	*Triticum aestivum* L.	BBAADD (2n=6x)	Chinese Spring[a]	Ch.	W.S.	Ch.	W.S.	Ch.
Wheat	*Triticum aestivum* L.	BBAADD (2n=6x)	RB07[a]	Ch.	W.S.	Ch.	W.S.	Ch.
Wheat	*Triticum aestivum* L.	BBAADD (2n=6x)	Green Spring[a]	Ch.	W.S.	Ch.	W.S.	Ch.
Wheat	*Triticum aestivum* L.	BBAADD (2n=6x)	AGS 2055[b]	Ch.	W.S.	Ch.	W.S.	Ch.
Wheat	*Triticum aestivum* L. *compactum* (Host) MacKey	BBAADD (2n=6x)	Cara[c]	W.S. + Ch.	W.S.	N.A.	W.S.	N.A.
Durum Wheat	*Triticum turgidum* L. ssp. *durum* (Desf.) Husnot	BBAA (2n=4x)	Lustre[d]	W.S.	W.S.	Ch. / W.S.	W.S.	W.S.
Durum Wheat	*Triticum turgidum* L. ssp. *durum* (Desf.) Husnot	BBAA (2n=4x)	ND Riveland[d]	Ch. / W.S.	W.S.	Ch. / W.S.	W.S.	Ch. / W.S.
Einkorn Wheat	*Triticum monococcum* ssp. *boeoticum*	AA (2n)	-	Ch. / W.S.	Ch. / W.S.	Ch. / W.S.	W.S.	W.S.
Emmer Wheat	*Triticum turgidum* ssp. *dicoccum*	BBAA (2n=4x)	-	W.S.	W.S.	W.S.	W.S.	W.S.
Barley	*Hordeum vulgare* L.	(2n)	Morex[e]	W.S.	W.S.	W.S.	W.S.	W.S.
Barley	*Hordeum vulgare* L.	(2n)	Kewaunee[e]	W.S.	W.S.	W.S.	W.S.	W.S.
Barley	*Hordeum vulgare* L.	(2n)	Conlon[f]	W.S.	W.S.	W.S.	W.S.	W.S.
Barley	*Hordeum vulgare* L.	(2n)	ND Pinnacle[f]	W.S.	W.S.	W.S.	W.S.	W.S.
Rye (Spring)	*Secale cereale* L.	(2n)	-	Ch.	Ch.	Ch.	W.S.	W.S. / W.S. + Ch.
Rye (Winter)	*Secale cereale* L.	(2n)	-	Ch.	Ch.	Ch.	W.S.	W.S.
Rye	*Secale cereale* L.	(2n)	AC Remington[g]	Ch. / W.S. + Ch.	Ch.	Ch. / W.S. + Ch.	W.S.	W.S.
Triticale	*x Triticosecale Wittmack*	BBAARR (2n=6x)	-	Ch.	W.S.	Ch.	W.S.	W.S.
Triticale	*x Triticosecale Wittmack*	BBAARR (2n=6x)	-	Ch. / W.S. + Ch.	W.S.	Ch. / W.S. + Ch.	W.S.	W.S.
Triticale	*x Triticosecale Wittmack*	BBAARR (2n=6x)	H	Ch.	W.S.	Ch.	Ch. / W.S.	W.S.
Oat	*Avena sativa L.*	AACCDD (2n=6x)	Antigo[h]	Ch. + W.S. edges	Ch. + W.S. edges	N.A.	W.S.	W.S.
Intermediate Wheatgrass	*Thinopyrum intermedium*	$E^eE^eE^bE^bStSt$ (2n=6x)	-	Ch.	Ch.	Ch.	W.S.	W.S.

Leaf inoculations were performed by syringe infiltration (OD_{600} of 0.1) using two-week-old plants, and symptoms were visualized at three days post inoculation.
[a]Hard Red Spring Wheat; [b]Soft Red Winter Wheat; [c]Soft White Winter Wheat; [d]Spring Durum Wheat; [e]Six-row Spring Barley; [f]Two-row Spring Barley; [g]Winter Rye; [h]Spring oat.
Ch. – chlorosis (non-host response); W.S. – water soaking (symptom development – susceptibility); N.A. – not analyzed. + indicates both symptoms present in the same inoculated leaf area; / indicates symptoms identified in different inoculated leaf areas.
