# Supplementary material for: Effector loss and gain drives host range at a fitness cost
Source: PLoS Pathog. 2026 Aug 3;22(8):e1014145. doi: 10.1371/journal.ppat.1014145 (PMC13472402; doi:10.1371/journal.ppat.1014145)
Supplement: Supplementary file 3 [file ppat.1014145.s006.pdf]

S4 Table. Symptom development assessment in a subset of the cereal genotype diversity panel inoculated with Xtt and Xtu strains.

				Xanthomonas translucens strain					
				**Xtt UPB820 WT**	**Xtt UPB820 ∆*xopAJ***	**Xtt UPB820 ∆*xopAJ*/∆*xopAJ***	**Xtt UPB820 ∆*xopE5***	**Xtt UPB820 ∆hopW1**	**Xtu UPB513 WT**
**Host**	**Scientific name**	**Genotype**	**Cultivar/variety/ genotype/line**	**Symptom**					
Wheat	*Triticum aestivum* L.	BBAADD (2n=6x)	AGS 2055[a]	Ch.	Ch.	N.A.	Ch.	Ch.	W.S.
Durum Wheat	*Triticum turgidum* L. ssp. *durum* (Desf.) Husnot	BBAA (2n=4x)	Lustre[b]	W.S.	W.S.	N.A.	W.S.	W.S.	W.S.
Einkorn Wheat	*Triticum monococcum* ssp. *boeoticum*	AA (2n)	-	Ch. + W.S. spots	Ch.	N.A.	Ch. + W.S. spots	Ch. + W.S. spots	W.S.
Emmer Wheat	*Triticum turgidum* ssp. *dicoccum*	BBAA (2n=4x)	-	W.S.	W.S.	N.A.	W.S.	W.S.	W.S.
Barley	*Hordeum vulgare* L.	(2n)	Morex[c]	W.S.	W.S.	N.A.	W.S.	W.S.	W.S.
Barley	*Hordeum vulgare* L.	(2n)	Conlon[d]	W.S.	W.S.	N.A.	W.S.	W.S.	W.S.
Rye (Spring)	*Secale cereale* L.	(2n)	-	Ch.	Ch.	N.A.	Ch.	Ch.	W.S.
Rye (Winter)	*Secale cereale* L.	(2n)	-	Ch.	Ch.	N.A.	Ch.	Ch.	W.S.
Triticale	*x Triticosecale Wittmack*	BBAARR (2n=6x)	-	Ch.	Ch.	N.A.	Ch.	Ch.	W.S.
Oat	*Avena sativa L.*	AACCDD (2n=6x)	Antigo[g]	Ch. + W.S. edges	W.S.	Ch. + W.S. edges	Ch. + W.S. edges	Ch. + W.S. edges	W.S.
Intermediate Wheatgrass	*Thinopyrum intermedium*	$E^eE^eE^bE^bStSt$ (2n=6x)	-	Ch.	Ch.	N.A.	Ch.	Ch.	W.S.

Leaf inoculations were performed by syringe infiltration (OD_{600} of 0.1) using two-week-old plants, and symptoms were visualized at three days post inoculation.
[a]Soft Red Winter Wheat; [b]Spring Durum Wheat; [c]Six-row Spring Barley; [d]Two-row Spring Barley; [g]Spring oat.
Ch. – chlorosis (non-host response); W.S. – water soaking (symptom development – susceptibility); N.A. – not analyzed. + indicates both symptoms present in the same inoculated leaf area; / indicates symptoms identified in different inoculated leaf areas.
