# Supplementary material for: Effector loss and gain drives host range at a fitness cost
Source: PLoS Pathog. 2026 Aug 3;22(8):e1014145. doi: 10.1371/journal.ppat.1014145 (PMC13472402; doi:10.1371/journal.ppat.1014145)
Supplement: Supplementary file 4 [file ppat.1014145.s007.pdf]

S6 Table. Bacterial strains and plasmids used in this study.

Bacterial strain	Genotype or description	Source
***Xanthomonas translucens* pv. translucens (Xtt)**		
UPB820 WT	Wild-type Xtt strain UPB820	(1, 2)
UPB820 Δ*hrcT*	UPB820 with chromosomal *hrcT* deletion	This study
UPB820 Δ*xopAJ*	UPB820 with chromosomal *xopAJ* deletion	This study
UPB820 Δ*xopAL1*	UPB820 with chromosomal *xopAL1* deletion	This study
UPB820 Δ*xopE5*	UPB820 with chromosomal *xopE5* deletion	This study
UPB820 Δ*hopW1*	UPB820 with chromosomal *hopW1* deletion	This study
UPB820 Δ*xopAL1/xopAL1*	Complemented strain of UPB820 Δ*xopAL1*	This study
UPB820 Δ*xopAJ/xopAJ*	Complemented strain of UPB820 Δ*xopAJ*	This study
UPB820 WT + pPneo-GFP	UPB820 WT bearing the pPneo-GFP plasmid for GFP expression, Km^R	This study
UPB820 Δ*xopAL1* + pPneo-GFP	UPB820 Δ*xopAL1* bearing the pPneo-GFP plasmid for GFP expression, Km^R	This study
UPB820 Δ*xopAL1/xopAL1* + pPneo-GFP	UPB820 Δ*xopAL1/xopAL1* bearing the pPneo-GFP plasmid for GFP expression, Km^R	This study
UPB787 WT	Wild-type Xtt strain UPB787	(1)
UPB787 Δ*xopAL1*	UPB787 with chromosomal *xopAL1* deletion	This study
XtKm7 WT	Wild-type Xtt strain XtKm7	(3)
XtKm7 Δ*xopAL1*	XtKm7 with chromosomal *xopAL1* deletion	This study
***Xanthomonas translucens* pv. undulosa (Xtu)**		
UPB513 WT	Wild-type Xtu strain UPB513	(1)
UPB513 Δ*hrcT*	UPB513 with chromosomal *hrcT* deletion	This study
UPB513 Δ*xopE4*	UPB513 with chromosomal *xopE4* deletion	This study
UPB513 miniTn*7*::Gm	UPB513 bearing genomic insertion using the pUC18miniTn*7*T-Gm empty vector, Gm^R	This study
UPB513 miniTn*7*::*xopAJ*	UPB513 bearing genomic insertion using the pUC18miniTn*7*T-Gm-*xopAJ* vector for expression of *xopAJ* from Xtt UPB820, Gm^R	This study
UPB513 miniTn*7*::*xopAL1*	UPB513 bearing genomic insertion using the pUC18miniTn*7*T-Gm-*xopAL1* vector for expression of *xopAL1* from Xtt UPB820, Gm^R	This study
UPB513 miniTn*7*::*xopE5*	UPB513 bearing genomic insertion using the pUC18miniTn*7*T-Gm-*xopE5* vector for expression of *xopE5* from Xtt UPB820, Gm^R	This study
UPB513 miniTn*7*::*hopW1*	UPB513 bearing genomic insertion using the pUC18miniTn*7*T-Gm-*hopW1* vector for expression of *hopW1* from Xtt UPB820, Gm^R	This study
UPB513 WT + pPneo-GFP	UPB513 WT bearing the pPneo-GFP plasmid for GFP expression, Km^R	(4)

UPB513 + pBBR1-*xopAL1*$_{\mathrm{Xcc8004}}$	UPB513 bearing the pBBR1-*xopAL1*$_{\mathrm{Xcc8004}}$ plasmid for expression of *xopAL1* from *Xanthomonas campestris* pv. campestris (Xcc) strain 8004, Km^R	This study
Escherichia coli		
DH10B	F– *mcrA* Δ(*mrr-hsdRMS-mcrBC*) φ80*lacZ*ΔM15 Δ*lacX74* *recA1 endA1 araD139* Δ (*ara-leu*)7697 *galU* *galK* λ– *rpsL*(Str^R) *nupG*	ThermoFisher
WM3064	thrB1004 pro thi rpsL hsdS lacZΔM15 RP4-1360 Δ(araBAD)567 ΔdapA1341::[erm pir]	(5)
**Plasmids**		
pK18mobsacB	*sacB* mutagenesis plasmid, Km^R	(6)
pUC18miniTn*7*T-Gm	Plasmid used for genomic insertion via miniTn*7*, Gm^R	(7)
pTNS3	Plasmid used for transposition of miniTn*7*, Ap^R	(8)
pK18mobsacB::Δ*hrcT*Xtt$_{\mathrm{UPB820}}$	*sacB* mutagenesis plasmid for Xtt UPB820 Δ*hrcT* deletion, Km^R	This study
pK18mobsacB::Δ*xopAJ*Xtt$_{\mathrm{UPB820}}$	*sacB* mutagenesis plasmid for Xtt UPB820 Δ*xopAJ* deletion, Km^R	This study
pK18mobsacB::Δ*xopAL1*Xtt$_{\mathrm{UPB820}}$	*sacB* mutagenesis plasmid for Xtt UPB820 Δ*xopAL1* deletion, Km^R	This study
pK18mobsacB::Δ*xopE5*Xtt$_{\mathrm{UPB820}}$	*sacB* mutagenesis plasmid for Xtt UPB820 Δ*xopE5* deletion, Km^R	This study
pK18mobsacB::Δ*hopW1*Xtt$_{\mathrm{UPB820}}$	*sacB* mutagenesis plasmid for Xtt UPB820 Δ*hopW1* deletion, Km^R	This study
pK18mobsacB::*xopAL1*Xtt$_{\mathrm{UPB820}}$-COMP	*sacB* mutagenesis plasmid designed to complement Xtt UPB820 Δ*xopAL1* by inserting *xopAL1* back into its original locus, Km^R	This study
pK18mobsacB::Δ*xopAL1*Xtt$_{\mathrm{UPB787}}$	*sacB* mutagenesis plasmid for Xtt UPB886 Δ*xopAL1* deletion, Km^R	This study
pK18mobsacB::Δ*xopAL1*Xtt$_{\mathrm{XtKm7}}$	*sacB* mutagenesis plasmid for Xtt CIX95 Δ*xopAL1* deletion, Km^R	This study
pK18mobsacB::Δ*hrcT*Xtu$_{\mathrm{UPB513}}$	*sacB* mutagenesis plasmid for Xtu UPB513 Δ*hrcT* deletion, Km^R	This study
pK18mobsacB::Δ*xopE4*Xtu$_{\mathrm{UPB513}}$	*sacB* mutagenesis plasmid for Xtu UPB513 Δ*xopE4* deletion, Km^R	This study
pUC18miniTn*7*T-Gm::*xopAJ*Xtt$_{\mathrm{UPB820}}$	Plasmid used for miniTn*7*::*xopAJ* (from Xtt UPB820; including native promoter and terminator regions) genomic insertion, Gm^R	This study
pUC18miniTn*7*T-Gm::*xopAL1*Xtt$_{\mathrm{UPB820}}$	Plasmid used for miniTn*7*::*xopAL1* (from Xtt UPB820; including native promoter and terminator regions) genomic insertion, Gm^R	This study
pUC18miniTn*7*T-Gm::*xopE5*Xtt$_{\mathrm{UPB820}}$	Plasmid used for miniTn*7*::*xopE5* (from Xtt UPB820; including native promoter and terminator regions) genomic insertion, Gm^R	This study
pUC18miniTn*7*T-Gm::*hopW1*Xtt$_{\mathrm{UPB820}}$	Plasmid used for miniTn*7*::*hopW1* (from Xtt UPB820; including native promoter and terminator regions) genomic insertion, Gm^R	This study

pPneo-GFP	Plasmid for GFP expression under the control of the promoter of neomycin resistant gene, Km^R	(9)
pBBR1MCS-2	RK2 broad-host-range cloning vector, Km^R	(10)
pEL667	Domesticated GoldenGate-compatible derivative of pBBR1MCS-2, *Bsa*I cohesive ends GGAG and AGCG, Km^R	This study
pEL667-*xopAL1*$_{\text{Xcc8004}}$	pEL667 carrying *xopAL1* from Xcc 8004 including native promoter and terminator regions, Km^R	This study
pRK2073	Helper plasmid, Sm^R	(11)

Km^R – kanamycin-resistant; Gm^R – gentamicin-resistant; Rif^R – rifampicin-resistant; Ap^R – ampicillin-resistant; Sm^R – streptomycin-resistant.

References for S6 Table

1. Bragard C, Singer E, Alizadeh A, Vauterin L, Maraite H, Swings J. Xanthomonas translucens from small grains: diversity and phytopathological relevance. Phytopathology. 1997;87:1111–7.
2. Alizadeh A, Barrault G, Sarrafi A, Rahimian H, Albertini L. Identification of bacterial leaf streak of cereals by their phenotypic characteristics and host range in Iran. European Journal of Plant Pathology. 1995;101:225–9.
3. Shah SMA, Khojasteh M, Wang Q, Taghavi SM, Xu Z, Khodaygan P, et al. Genomics-enabled novel insight into the pathovar-specific population structure of the bacterial leaf streak pathogen Xanthomonas translucens in small grain cereals. Front Microbiol. 2021;12:674952.
4. Gluck-Thaler E, Cerutti A, Perez-Quintero AL, Butchacas J, Roman-Reyna V, Madhavan VN, et al. Repeated gain and loss of a single gene modulates the evolution of vascular plant pathogen lifestyles. Sci Adv. 2020;6:eabc4516.
5. Dehio C, Meyer M. Maintenance of broad-host-range incompatibility group P and group Q plasmids and transposition of Tn5 in Bartonella henselae following conjugal plasmid transfer from Escherichia coli. J Bacteriol. 1997;179:538–40.
6. Schäfer A, Tauch A, Jäger W, Kalinowski J, Thierbach G, Pühler A. Small mobilizable multi-purpose cloning vectors derived from the Escherichia coli plasmids pK18 and pK19: selection of defined deletions in the chromosome of Corynebacterium glutamicum. Gene. 1994;145:69–73.
7. Choi KH, Schweizer HP. mini-Tn7 insertion in bacteria with single attTn7 sites: example Pseudomonas aeruginosa. Nat Protoc. 2006;1(1):153–61.
8. Choi KH, Mima T, Casart Y, Rholl D, Kumar A, Beacham IR, et al. Genetic tools for select-agent-compliant manipulation of Burkholderia pseudomallei. Appl Environ Microbiol. 2008;74(4):1064–75.
9. Han SW, Park CJ, Lee SW, Ronald PC. An efficient method for visualization and growth of fluorescent Xanthomonas oryzae pv. oryzae in planta. BMC Microbiol. 2008;8:164.
10. Kovach ME, Elzer PH, Hill DS, Robertson GT, Farris MA, Roop RM, et al. Four new derivatives of the broad-host-range cloning vector pBBR1MCS, carrying different antibiotic-resistance cassettes. Gene. 1995;166:175–6.

11. Leong SA, Ditta GS, Helinski DR. Heme biosynthesis in Rhizobium: identification of a cloned gene coding for δ-aminolevulininc acid synthetase from Rhizobium meliloti. J Biol Chem. 1982;257(15):8724–30.
