# Supplementary material for: Effector loss and gain drives host range at a fitness cost
Source: PLoS Pathog. 2026 Aug 3;22(8):e1014145. doi: 10.1371/journal.ppat.1014145 (PMC13472402; doi:10.1371/journal.ppat.1014145)
Supplement: Supplementary file 5 [file ppat.1014145.s008.pdf]

S7 Table. List of genes deleted in *X. translucens* in this study.

Locus tag[a]	Gene	Genome annotation[a]
***Xanthomonas translucens* pathovar translucens**		
KFS83_06010	*hrcT*	Type III secretion system export apparatus subunit SctT
KFS83_17905	*xopAJ*	Zeta toxin family protein
KFS83_12860	*xopAL1*	Type III effector
KFS83_00330	*xopE5*	Type III effector HopX1
KFS83_14440	*hopW1*	Type III effector
***Xanthomonas translucens* pathovar undulosa**		
F0H33_05575	*hrcT*	EscT/YscT/HrcT family type III secretion system export apparatus protein
F0H33_00965	*xopE4*	Hypothetical protein

[a]Locus tags and genome annotations are from genomes available online at NCBI, which are used here as references. Strain UPB947 is the reference for *X. translucens* pv. translucens (GenBank Accession number CP076248), and strain P3 is the reference for *X. translucens* pv. undulosa (GenBank Accession number CP043500).
