# Supplementary material for: Effector loss and gain drives host range at a fitness cost
Source: PLoS Pathog. 2026 Aug 3;22(8):e1014145. doi: 10.1371/journal.ppat.1014145 (PMC13472402; doi:10.1371/journal.ppat.1014145)
Supplement: Supplementary file 6 [file ppat.1014145.s009.pdf]

S8 Table. Oligonucleotide primers used in this study.

Name	Sequence (5'-3')	Purpose	Amplicon size (bp)	Source
**Oligonucleotide primers used to knockout genes of interest in respective strains**				
XttUPB820-hrcT-UP-F	ctctagagtcgacctgcaggcatgcaagctgatgtgaccgatttccagcagtcgctgtgg	Amplify the upstream region of *hrcT* (Xtt UPB820) for cloning into pK18mobsacB	663	This study
XttUPB820-hrcT-UP-R	agagcgcaactcatacagtgcgcgatggtccatgaacagcacgaagcaacgcacgctcgc			This study
XttUPB820-hrcT-Down-F	gcgagcgtgcgttgcttcgtgctgttcatggaccatcgcgcactgtatgagttgcgctct	Amplify the downstream region of *hrcT* (Xtt UPB820) for cloning into pK18mobsacB	549	This study
XttUPB820-hrcT-Down-R	acgttgtaaaacgacggccagtgccaagctctgctgaagcatctgccaggcggtattacg			This study
hrcT-conf-F	cttcgtgctgttcatggtgctg	Amplify an internal region of *hrcT* (Xtt UPB820 and Xtu UPB513) to confirm deletion	679	This study
hrcT-conf-R	gaaggacagttcgtccagcaac			This study
XttUPB820-xopAJ-UP-F	ctctagagtcgacctgcaggcatgcaagctcatcgtcggatcgctcagcgcctaccattg	Amplify the upstream region of *xopAJ* (Xtt UPB820) for cloning into pK18mobsacB	533	This study
XttUPB820-xopAJ-UP-R	ctgccaagccatatttttcgctctcaatccccaggaaaatgcgatgacccacttaaaagg			This study
XttUPB820-xopAJ-Down-F	ccttttaagtgggtcatcgcattttcctggggattgagagcgaaaaatatggcttggcag	Amplify the downstream region of *xopAJ* (Xtt UPB820) for cloning into pK18mobsacB	537	This study
XttUPB820-xopAJ-Down-R	acgttgtaaaacgacggccagtgccaagctgccgtagacgtcgccgatgaacaggttgag			This study
xopAJXttUPB820 conf-F	gatgtttggactgggtgagcttc	Amplify an internal region of *xopAJ* (Xtt UPB820) to confirm deletion	670	This study
xopAJXttUPB820 conf-R	gaacctgtcgatgacatctaagc			This study
XttUPB820-xopAL1-UP-F	ctctagagtcgacctgcaggcatgcaagctaccgtatcccgatcgtggcggtgaccaatc	Amplify the upstream region of *xopAL1* (Xtt UPB820) for cloning into pK18mobsacB	801	This study
XttUPB820-xopAL1-UP-R	cgtacgacactacaacgcctcatgatcgactgcaactgaagagcggcgagcaacccgcag			This study
XttUPB820-xopAL1-Down-F	ctgcgggttgctcgccgctcttcagttgcagtcgatcatgaggcgttgtagtgtcgtacg	Amplify the downstream region of *xopAL1* (Xtt UPB820) for cloning into pK18mobsacB	577	This study
XttUPB820-xopAL1-Down-R	acgttgtaaaacgacggccagtgccaagctaacccgttccacccgttcagcgtgttcgac			This study
xopAL1Xtt-conf-F	ccttatggcgctggaaatcag	Amplify an internal region of *xopAL1* (all Xtt strains) to confirm	605	This study
xopAL1Xtt-conf-R	gacatgatcgacgagcgatac			This study

		deletion and complementation		
XttUPB820-xopE5-UP-F	ctctagagtcgacctgcaggcatgcaagctgatcttcaccggcaacgacgaaatggcggc	Amplify the upstream region of *xopE5* (Xtt UPB820) for cloning into pK18mobsacB	585	This study
XttUPB820-xopE5-UP-R	gcaccaggtgaacgccgtcaggcggggcgcacgcaactccaatgtgtgcaagcgcccgcg			This study
XttUPB820-xopE5-Down-F	cgcgggcgcttgcacacattggagttgcgtgcgccccgcctgacggcgttcacctggtgc	Amplify the downstream region of *xopE5* (Xtt UPB820) for cloning into pK18mobsacB	720	This study
XttUPB820-xopE5-Down-R	acgttgtaaaacgacggccagtgccaagctcgatgtactggtagatggtgcgcgcgttgc			This study
xopE5XttUPB820 conf-F	gttcgtcgagtatgtcgagcaaatg	Amplify an internal region of *xopE5* (Xtt UPB820) to confirm deletion	503	This study
xopE5XttUPB820 conf-R	catcgttgagcacatgctgttc			This study
XttUPB820-hopW1-UP-F	ctctagagtcgacctgcaggcatgcaagctaagtctgccaaaaaccgtaagcacatcacc	Amplify the upstream region of *hopW1* (Xtt UPB820) for cloning into pK18mobsacB	720	This study
XttUPB820-hopW1-UP-R	cggctatgaatctctcgctcataggcggattgaacaaagcatagctcgcggctgagtgta			This study
XttUPB820-hopW1-Down-F	tacactcagccgcgagctatgctttgttcaatccgcctatgagcgagagattcatagccg	Amplify the downstream region of *hopW1* (Xtt UPB820) for cloning into pK18mobsacB	632	This study
XttUPB820-hopW1-Down-R	acgttgtaaaacgacggccagtgccaagcttatccgctgcattcggatggcacagcagta			This study
hopW1XttUPB820conf-F	gcaacaatgctcgatctagatgatgg	Amplify an internal region of *hopW1* (Xtt UPB820) to confirm deletion	592	This study
hopW1XttUPB820conf-R	ccatcgtggacaaaggtaaaagacg			This study
XtuUPB513-hrcT-UP-F	ctctagagtcgacctgcaggcatgcaagctgacgatgatgcaggtattgcaacgcctgcg	Amplify the upstream region of *hrcT* (Xtu UPB513) for cloning into pK18mobsacB	682	This study
XtuUPB513-hrcT-UP-R	gtgcgttgcttcgtgctgttcatggtgctgttgctgatgctggcgttgctgatgcaagtg			This study
XtuUPB513-hrcT-Down-F	cacttgcatcagcaacgccagcatcagcaacagcaccatgaacagcacgaagcaacgcac	Amplify the downstream region of *hrcT* (Xtu UPB513) for cloning into pK18mobsacB	719	This study
XtuUPB513-hrcT-Down-R	acgttgtaaaacgacggccagtgccaagctatttccagcagtcgctgtggcacttgcagc			This study
xopE4XtuUPB513 conf-F	gatgcaatcgccggagctatcatc	Amplify an internal region of *xopE4* (Xtu	638	This study

xopE4XtuUPB513 conf-R	ctctgcttttgtccatcgaccaagc	UPB513) to confirm deletion		This study
M13-F	gtaaaacgacggccag	Amplify an internal region of pK18mobsacB to confirm cloning of regions of interest	variable	(1)
M13-R	caggaaacagctatgac			
**Oligonucleotide primers used to insert genes into XtuUPB513 via miniTn*7***				
XttUPB820COMPxopAJ-ORF-F	cttggcgcttttaaaaatttccttggccgccgcatttcctggtcagatcaatgaactgt	Amplify *xopAJ* (Xtt UPB820) including promoter (272 bp) for cloning into pUC18miniTn*7*T-Gm	1,542	This study
XttUPB820COMPxopAJ-ORF-R	aaggccttcgcgaggtaccgggcccaagctgcgagaggcgccagtcatgtgtgttgcaac			This study
XttUPB820COMPxopAJ-term-F	ccccgggctgcaggaattcctcgagaagctcatcgtcggatcgctcagcgcctaccattg	Amplify terminator region of *xopAJ* (Xtt UPB820) for cloning into pUC18miniTn*7*T-Gm	223	This study
XttUPB820COMPxopAJ-term-R	acagttcattgatctgaccaggaaaatgcggcggccaaggaaatttttaaaagcgccaag			This study
XttUPB820COMPxopAL1-F	ccccgggctgcaggaattcctcgagaagctacgaatagcaggttgcgggtcatgtcgcgt	Amplify *xopAL1* (Xtt UPB820) including promoter (338 bp) and terminator (248 bp) for cloning into pUC18miniTn*7*T-Gm	1,504	This study
XttUPB820COMPxopAL1-R	aaggccttcgcgaggtaccgggcccaagctccgaaggacaaccgctcatccaagacgacg			This study
XttUPB820COMPxopE5-F	ccccgggctgcaggaattcctcgagaagctaagactgatccgaggcgagcgtcagcctgc	Amplify *xopE5* (Xtt UPB820) including promoter (313 bp) and terminator (163 bp) for cloning into pUC18miniTn*7*T-Gm	1,598	This study
XttUPB820COMPxopE5-R	aaggccttcgcgaggtaccgggcccaagctgtacgggccattctagccgccatggttaac			This study
XttUPB820COMPhopW1-F	ccccgggctgcaggaattcctcgagaagctcctctcaacatacaccggtgctcacatcaa	Amplify *hopW1* (Xtt UPB820) including promoter (405 bp) and terminator (265 bp) for cloning into pUC18miniTn*7*T-Gm	1,960	This study
XttUPB820COMPhopW1-R	aaggccttcgcgaggtaccgggcccaagctcagaacacgtgatcgatcaaatggtcgcgc			This study
miniTN7-conf-F	cgttcggtcaaggttctgga	Amplify an internal region of pUC18miniTn*7*T-Gm to confirm cloning of regions of interest	variable	This study
miniTn7-conf-R	gctggccgataagctctgat			This study
**Oligonucleotide primers used to clone *xopAL1*$_{Xcc8004}$ into pEL667 replicative plasmid**				
LM-169-XC_2995 xopAL1	tggtctctggagttctgaagcatgtggtcgg	Amplify *xopAL1* 5' region for Golden Gate cloning into pEL667	850	This study
LM170-XC_2995 xopAL1	tggtctcgtcctcatctgcgtcgcttactc			
LM171-XC_2995 xopAL1	tggtctcgaggaccatgcttacgtgctga		568	This study

LM-172-XC_2995 xopAL1	tggtctcaagcgttcctcggagtaggaaatca	Amplify *xopAL1* 3' region for Golden Gate cloning into pEL667		

Reference for S8 Table

1. Messing J. New M13 vectors for cloning. Methods Enzymol. 1983;101:20–78.
