# Supplementary material for: Effector loss and gain drives host range at a fitness cost
Source: PLoS Pathog. 2026 Aug 3;22(8):e1014145. doi: 10.1371/journal.ppat.1014145 (PMC13472402; doi:10.1371/journal.ppat.1014145)
Supplement: Supplementary file 7 [file ppat.1014145.s010.pdf]

S9 Table. List of genomes used in this study.

Strain	GenBank Accession number	Reference
***Xanthomonas translucens* pv. translucens (Xtt)**		
B1FA	CP090000	Unpublished
CIX43	CP072988/CP072989	(1)
CIX84	CP159782	This study
CIX95	CP072990	(1)
Colorado	https://github.com/Merfa-lab/Genomes	This study
DSM 18974	LT604072	(2)
UPB458	CP076249	(3)
UPB545	CP159781	This study
UPB787	https://github.com/Merfa-lab/Genomes	This study
UPB820[a]	https://github.com/Merfa-lab/Genomes	This study
UPB886	GCA_009600865.1	(4)
UPB947	CP076248	(3)
XtKm7	CP064005	(5)
XtKm8	CP064004	(5)
XtKm9	CP064003	(5)
XtKm34	CP064001	(5)
***Xanthomonas translucens* pv. undulosa (Xtu)**		
CFBP 2055	CP074361/CP074362	(3)
CFBP 2539	CP074363	(3)
CIX162	CP093449	(6)
CIX207	CP093448	(6)
CIX282	CP093447	(6)
CIX303	CP093446	(6)
CIX40	CP093450	(6)
ICMP 11055	CP009750	(7)
LW16	CP043540	(8)
MAI5034	CP089584	(9)
P3	CP043500	(8)
UPB513[a]	JBEFAI000000000	This study
XtFa1	CP063996	(5)
XtKm12	CP064000	(5)
XtKm15	CP063997/CP063998/CP063999	(5)
XtLr8	CP063993/CP063994/CP063995	(5)
Xtu 4699	CP008714	Unpublished
***Xanthomonas translucens* pv. arrhenateri (Xta)**		
LMG 727	CP086333	(3)
***Xanthomonas translucens* pv. cerealis (Xtc)**		
01	CP038228	(10)
CFBP 2541	CP074364	(3)
***Xanthomonas translucens* pv. graminis (Xtg)**		
LMG 726	CP076254	(3)
ART-Xtg9	CP076252	(11)
ART-Xtg2	CP076253	(11)
ART-Xtg29	CP076257	(11)

NCPPB 3709	CP118161/CP118162	(11)
***Xanthomonas translucens* pv. phleipratensis (Xtphlei)**		
LMG 730	CP076251	(3)
LMG 843	CP086332	(3)
***Xanthomonas translucens* pv. pistaciae (Xtp)**		
CFBP 8304	CP074365	(3)
ICMP 16317	CP083804	(3)
***Xanthomonas translucens* pv. poae (Xtpo)**		
LMG 728	CP076250	(3)
NCPPB 3711	CP076255/CP076256	Unpublished
Xanthomonas albilineans		
Xa-FJ1[a]	CP046570.1/CP046571.1	(12)
***Xanthomonas arboricola* (Xa)**		
YchA	CP090441	Unpublished
***Xanthomonas arboricola* pv. juglandis**		
CFBP 2528[a]	GCA_001013475.1	(13)
***Xanthomonas axonopodis* pv. cassiae**		
NCPPB 645	CP168000.1	Unpublished
***Xanthomonas axonopodis* pv. vasculorum**		
NCPPB 796[a]	CP053649.1/CP053650.1/CP053651.1	Unpublished
Xanthomonas bromi		
LMG 947	GCA_900092025.1	Unpublished
***Xanthomonas campestris* (Xc) pv. campestris (Xcc)**		
40-2	CP067001	(14)
3054	CP066963/CP066964/CP066965	(14)
8004	CP000050	(15)
CFBP 12825	CP066929	(14)
CFBP 5251[a]	AE008922.1	(16)
CN07	CP101174/CP101175	Unpublished
Xanthomonas cannabis		
8590[a]	GCA_011761725.1	Unpublished
***Xanthomonas euvesicatoria* (Xe)**		
T0319-01	CP137539.1	(17)
***Xanthomonas euvesicatoria* pv. perforans**		
GEV872[a]	CP116305.1/CP116306.1/CP116307.1/CP116308.1	Unpublished
***Xanthomonas euvesicatoria* pv. polysciadis**		
Pgu1	CP180391.1	Unpublished
***Xanthomonas fragariae* (Xf)**		
YLX21	CP114134.1	(18)

Xanthomonas hortorum		
B07-007[a]	CP016878.1/CP016879.1	Unpublished
***Xanthomonas hortorum* pv. hederae (Xhh)**		
22-338	GCA_028580375.1	(19)
***Xanthomonas hortorum* pv. pelargonii (Xhp)**		
OSU493	CP098604/CP098605	Unpublished
OSU498	CP098602/CP098603	Unpublished
***Xanthomonas hortorum* pv. vitians (Xhv)**		
LM16734	CP060399.1	(20)
Xanthomonas hyacinthi		
CFBP 1156	CP043476/CP043477	(21)
***Xanthomonas nasturtii* (Xn)**		
10015A	GCA_023337265.1	Unpublished
***Xanthomonas oryzae* pv. oryzicola**		
GX01[a]	CP043403.1/KR071788.1	Unpublished
BLS256	CP003057.2	(22)
***Xanthomonas populi* (Xp)**		
CFBP 1817	GCA_002940065.1	Unpublished
***Erwinia psidii* (Ep)**		
LPF 534	GCA_026549045.1	(23)
***Paraburkholderia nemoris* (Pn)**		
RL18-011-BIC-A	GCA_046027695.1	(24)
Paracidovorax citrulli		
AAC00-1	CP000512.1	Unpublished
DSM 17060	GCA_900100305.1	Unpublished
***Pseudomonas amygdali* (Pa)**		
CFBP 1650	GCA_041449455.1	Unpublished
***Ralstonia mannitolilytica* (Rm)**		
LMG 18090	GCA_958405575.1	(25)
Ralstonia solanacearum		
10314	GCA_008271875.1	(26)

[a] Indicates genomes that were used for representative *Xanthomonas* spp. phylogenomics and host range in Figs 1A and S1.

References for S9 Table

1. Heiden N, Roman-Reyna V, Curland RD, Dill-Macky R, Jacobs JM. Comparative genomics of barley-infecting Xanthomonas translucens shows overall genetic similarity but globally distributed virulence factor diversity. Phytopathology. 2023;113:2056–61.

2. Jaenicke S, Bunk B, Wibberg D, Sproer C, Hersemann L, Blom J, et al. Complete genome sequence of the barley pathogen Xanthomonas translucens pv. translucens DSM 18974T (ATCC 19319T). Genome Announc. 2016;4(6).
3. Goettelmann F, Roman-Reyna V, Cunnac S, Jacobs JM, Bragard C, Studer B, et al. Complete genome assemblies of all Xanthomonas translucens pathotype strains reveal three genetically distinct clades. Front Microbiol. 2021;12:817815.
4. Roman-Reyna V, Luna EK, Pesce C, Vancheva T, Chang C, Ziegle J, et al. Genome resource of barley bacterial blight and leaf streak pathogen Xanthomonas translucens pv. translucens strain UPB886. Plant Dis. 2020;104(1):13–5.
5. Shah SMA, Khojasteh M, Wang Q, Taghavi SM, Xu Z, Khodaygan P, et al. Genomics-enabled novel insight into the pathovar-specific population structure of the bacterial leaf streak pathogen Xanthomonas translucens in small grain cereals. Front Microbiol. 2021;12:674952.
6. Ledman KE, Roman-Reyna V, Curland RD, Heiden N, Jacobs J, Dill-Macky R. Comparative genomics of Xanthomonas translucens pv. undulosa strains isolated from weedy grasses and cultivated wild rice. Phytopathology. 2023;113:2083–90.
7. Falahi Charkhabi N, Booher NJ, Peng Z, Wang L, Rahimian H, Shams-Bakhsh M, et al. Complete genome sequencing and targeted mutagenesis reveal virulence contributions of Tal2 and Tal4b of Xanthomonas translucens pv. undulosa ICMP11055 in bacterial leaf streak of wheat. Front Microbiol. 2017;8:1488.
8. Peng Z, Hu Y, Zhang J, Huguet-Tapia JC, Block AK, Park S, et al. Xanthomonas translucens commandeers the host rate-limiting step in ABA biosynthesis for disease susceptibility. Proc Natl Acad Sci U S A. 2019;116(42):20938–46.
9. Clavijo F, Barrera C, Bencic A, Croce V, Jacobs JM, Bernal AJ, et al. Complete genome sequence resource for Xanthomonas translucens pv. undulosa MAI5034, a wheat pathogen from Uruguay. Phytopathology. 2022;112(9):2036–9.
10. Shah SMA, Haq F, Ma W, Xu X, Wang S, Xu Z, et al. Tal1(NXtc01) in Xanthomonas translucens pv. cerealis contributes to virulence in bacterial leaf streak of wheat. Front Microbiol. 2019;10:2040.
11. Goettelmann F, Koebnik R, Roman-Reyna V, Studer B, Kolliker R. High genomic plasticity and unique features of Xanthomonas translucens pv. graminis revealed through comparative analysis of complete genome sequences. BMC Genomics. 2023;24(1):741.
12. Zhang HL, Ntambo MS, Rott PC, Chen G, Chen LL, Huang MT, et al. Complete genome sequence reveals evolutionary and comparative genomic features of Xanthomonas albilineans causing sugarcane leaf scald. Microorganisms. 2020;8(2).
13. Cesbron S, Briand M, Essakhi S, Gironde S, Boureau T, Manceau C, et al. Comparative genomics of pathogenic and nonpathogenic strains of Xanthomonas arboricola unveil molecular and evolutionary events linked to pathoadaptation. Front Plant Sci. 2015;6:1126.
14. Dubrow ZE, Carpenter SCD, Carter ME, Grinage A, Gris C, Lauber E, et al. Cruciferous weed isolates of Xanthomonas campestris yield insight into pathovar genomic relationships and genetic determinants of host and tissue specificity. Mol Plant Microbe Interact. 2022;35(9):791–802.
15. Qian W, Jia Y, Ren SX, He YQ, Feng JX, Lu LF, et al. Comparative and functional genomic analyses of the pathogenicity of phytopathogen Xanthomonas campestris pv. campestris. Genome Res. 2005;15(6):757–67.

16. da Silva ACR, Ferro JA, Reinach FC, Farah CS, Furlan LR, Quaggio RB, et al. Comparison of the genomes of two Xanthomonas pathogens with differing host specificities. Nature. 2002;417(6887):459–63.
17. Huang CJ, Wu TL, Wu YL, Wang RS, Lin YC. Comparative genomic analysis uncovered phylogenetic diversity, evolution of virulence factors, and horizontal gene transfer events in tomato bacterial spot Xanthomonas euvesicatoria. Front Microbiol. 2024;15:1487917.
18. Wei F, Liang X, Shi J-C, Luo J-N, Qiu L-J, Li X-X, et al. Pan-Genomic Analysis Identifies the Chinese Strain as a New Subspecies of Xanthomonas fragariae. Plant Disease. 2023;108(1):45–9.
19. Iruegas-Bocardo F, Weisberg AJ, Riutta ER, Kilday K, Bonkowski JC, Creswell T, et al. Whole genome sequencing-based tracing of a 2022 introduction and outbreak of Xanthomonas hortorum pv. pelargonii. Phytopathology. 2023;113(6):975–84.
20. Morinière L, Lecomte S, Gueguen E, Bertolla F. In vitro exploration of the Xanthomonas hortorum pv. vitians genome using transposon insertion sequencing and comparative genomics to discriminate between core and contextual essential genes. Microb Genom. 2019;7(6).
21. Cohen SP, Luna EK, Lang JM, Ziegle J, Chang C, Leach JE, et al. High-quality genome resource of Xanthomonas hyacinthi generated via long-read sequencing. Plant Dis. 2020;104(4):1011–2.
22. Bogdanove AJ, Koebnik R, Lu H, Furutani A, Angiuoli SV, Patil PB, et al. Two new complete genome sequences offer insight into host and tissue specificity of plant pathogenic Xanthomonas spp. J Bacteriol. 2011;193(19):5450–64.
23. Alves FHNdS, Pereira IC, dos Santos SA, Monteiro ALR, Vidigal PMP, Alfenas AC, et al. Comprehensive prediction of plant cytoplasmic and apoplastic effectors underlying Erwinia psidii pathogenicity. Plant Pathology. 2023;72(1):130–43.
24. Paulo BS, Recchia MJJ, Lee S, Fergusson CH, Romanowski SB, Hernandez A, et al. Discovery of megapolipeptins by genome mining of a Burkholderiales bacteria collection. Chem Sci. 2024;15(40):16567–81.
25. Steyaert S, Peeters C, Wieme AD, Muyldermans A, Vandoorslaer K, Spilker T, et al. Novel Ralstonia species from human infections: improved matrix-assisted laser desorption/ionization time-of-flight mass spectrometry-based identification and analysis of antimicrobial resistance patterns. Microbiol Spectr. 2024;12(6):e0402123.
26. Bautista MAM, Llames JHS, Sabban EAV, Villegas LC. Draft genome sequences of Ralstonia solanacearum isolated from banana and tomato in the Philippines. Philippine Journal of Science. 2019;148:115–26.
