# Supplementary material for: Effector loss and gain drives host range at a fitness cost
Source: PLoS Pathog. 2026 Aug 3;22(8):e1014145. doi: 10.1371/journal.ppat.1014145 (PMC13472402; doi:10.1371/journal.ppat.1014145)
Supplement: Supplementary file 8 [file ppat.1014145.s020.docx]

***Re:* Manuscript PPATHOGENS-D-26-00734, “Effector loss and gain drives host range at a fitness cost”**

We would like to thank the editor and the three reviewers for their careful review of our manuscript and their constructive suggestions. We have addressed all comments raised by the peer reviewers and made the corresponding adjustments to the manuscript to the best of our knowledge. We believe that these changes and corrections have improved the quality of our study, and we are grateful for this.

**Note**: Specific comments taken from the editor and reviewers’ comments are included here in bold, followed by their respective responses. Line numbers of the corrections correspond to the marked-up manuscript version (“Revised Manuscript with Track Changes” file) using track changes.

**Editor’s comments to authors:**

**This study by Merfa et al is centered about how host-specialize organisms evolve into generalists. They tested the hypothesis that a generalist Xanthomonas translucens subgroup (Xtu) arose from a specialist ancestor (Xtt) via the loss of a single Type III effector encoding gene, xopAL1. Type III effectors have been implicated in host range before, but one reviewer notes that the novelty of this work is the use of natural variation to show that this phenomenon is happening naturally and is driving ecologically and economically important shifts in pathogen host range.**

**All 3 reviewers recommend revisions to the manuscript focused on restructuring of the manuscript to provide clarity and support to the findings. Reviewers 1 and 3 both suggest that moving some data out of the supplement into the main text would help provide support the main findings of the manuscript. It was also suggested that supplementary material be better synthesized so it is easier to understand the main findings. Reviewers 1 and 3 suggests adding additional explanation/support for the findings in barley.**

We thank the editor for carefully choosing expert reviewers to comment on our manuscript. We have addressed all questions and concerns raised by the reviewers, added the required data, and edited our manuscript accordingly. Please refer below to our responses to the suggestions made by the reviewers.

**Reviewers’ comments to authors**

**Part I - Summary**

**Reviewer #1:**

**Understanding the genetic factors that determines pathogen host range, and how pathogens evolve to infect new hosts, is critical to predicting and understanding emergent diseases. Using Xanthomonas translucens as a model, Merfa et al. study the genomics of host transitions and found that X. translucens subgroup arose from a specialist ancestor via the loss of a single effector gene, xopAL1. Deleting barley-specialist X. translucens xopAL1 was sufficient to result in a gain of virulence on wheat; however, loss of xopAL1 results in a decrease in virulence in barley highlighting an evolutionary tradeoff.

This study advances our understanding of host range and the evolution of new pathogens. While T3E have previously been shown to control host range and fitness tradeoffs between hosts, and even generalist/specialist transitions, I think the novelty of this work is the use of natural variation to show that this phenomenon is happening naturally and is driving ecologically and economically important shifts in pathogen host range.

I think the findings are strong, but the manuscript suffers a bit from trying to keep the main text brief with too many major findings buried in the supplement. There are also a lot of tangents that aren’t described in the abstract or the main summary. I don’t believe Plos Pathogens is restricted to 4 figures; thus I would strongly encourage the authors to reorganize and pull some of the data out of the supplement and put it in the main text. I think this would help tell a stronger and clearer story.**

We thank the reviewer for the thoughtful review of our manuscript and for the suggestions. We also appreciate the reviewer for highlighting the novelty of our study. We have edited our manuscript following the reviewer’s comments to better explain and support our findings, including the following:

- Supporting figures (screening of mutants, complemented, and insertion strains, growth curves, and results with *xopAJ* in oat and barley and its evolutionary analysis) were moved to the main text.
- Additional supporting figures were consolidated into less figures to make the information concise.
- A more detailed description of inoculation experiments, strains that were used and interpretation of leaf lesions was included. More background information was also provided to guide the readers outside the field of plant pathology.
- The discussion section was expanded to include additional comments raised by reviewers.
- The manuscript was reframed to present all XopAL1 results first and then focus on XopAJ. We believe this substantially increased the strength of the study.
- An additional supporting file was included containing all source (raw) data for quantitative analyses.

**Reviewer #2:**

**This manuscript presents strong and compelling evidence supporting the role of XopAL1 in host specificity between barley and wheat, and overall the study is well designed, experimentally rigorous, and of clear significance to the field of plant–microbe interactions. The combination of comparative genomics, mutant analysis, and infection assays provides an important contribution toward understanding how effector evolution shapes host adaptation in Xanthomonas translucens.**

We thank the reviewer for the kind comments and suggestions to our manuscript.

**Reviewer #3:**

**This manuscript by Merfa, Jacobs, and colleagues trace loss of Type III secretion effector XopAL1 as a key evolutionary step in development of generalist plant pathogens in the Xanthomonas translucens group. Type III secretion systems are known to be of widespread importance in plant pathogens broadly and Xanthomonas specifically, and this work derived from phylogenomic analysis to examine so-called non-TAL effectors in about a dozen generalist (Xtu) and barley-specialized (Xtt) strains. Loss of XopAL1 promotes pathogenesis in wheat (as shown by engineered loss in Xtt or introduction into the Xtu background). The work also shows that Xtu strains lacking XopAL1 have a fitness defect in barley, demonstrating a tradeoff to loss of this effector that inhibits fitness in barley while enhancing fitness in wheat (although I raise a question about this phenotype). The work also examines an effector gained in Xtt, xopAJ, and demonstrates a significant albeit milder specificity phenotype in that context.

The paper overall is strong in combining multiple approaches to yield a rich understanding of the interplay of bacterial effectors across evolution and how those effectors act in different bacterial-host contexts. The authors are to be applauded for delving into a wide strain collection to ask these questions, for testing their hypotheses in multiple isolates, and for distilling complicated ideas into compelling figures that capture the complexity of the work while still telling the story. My critical comments fall into two categories. First, the writing uses substantial jargon (plant pathogen, plant, genomics jargon, etc), and although I am deeply interested in the topic, it took me much longer to read as I had to dissect through these areas. I pointed out some of these issues, but overall I would recommend reevaluating much of the manuscript for places where additional context could guide the reader. Similarly, the key access point to the work is a double-negative phenotype (the chlorosis phenotype that correlates with protection) that could similarly use more background. I have a similar comment about making the data in the supplement more accessible to the reader. Second, understanding some of the key phenotypes was obscured and it was difficult to tease apart whether these were issues with the writing or the data/interpretation themselves. Point #1 below on the key phenotypes in barley was the most relevant concern. The comments below elaborate in more detail.**

We thank the reviewer for the insightful comments and thorough review of our manuscript. We have carefully edited the manuscript to address and clarify these concerns and also make it more digestible for readers outside the field of plant pathology.

**Part II – Major Issues: Key Experiments Required for Acceptance

Reviewer #1:**

**None**

**Reviewer #2:**

**Lines 151–152 may benefit from further clarification, where the manuscript refers to the “presence” and “absence” of effectors. The definition of “presence” should be expanded beyond simple gene detection. Specifically, it would be helpful to include information regarding amino acid sequence identity, potential truncations, and structural integrity of the predicted effectors. In addition, the authors may wish to discuss whether SNP-level variation within conserved effectors could contribute to host specificity, as even minor sequence changes may alter recognition or virulence functions.**

We thank the reviewer for the suggestion and agree that including a more detailed description of the comparative genomics analysis that we performed will be helpful to guide the readers. We added these details to the revised manuscript (lns. 171-173).

Although we agree that SNP-level variation of an effector gene, its truncation and/or genomic context (i.e. promoter and genomic locus), among other factors may affect pathogen host range, we chose to not perform these analyses since it is out of scope of our study. Variations among those genes are the focus of our ongoing research of host range in *X. translucens* and will be included in a future manuscript. Upon determining patterns of effector gene presence and absence between our specialist (Xtt) and generalist (Xtu) models, we focused our functional genomics analyses on the unique effectors detected between both. Thus, we believe that including this extensive information about the variation within conserved effector genes would be distracting for the reader since we did not perform functional analyses on this. We have mentioned that the hypothesis we are testing is that host range is modulated by patterns of effector gene presence/absence (lns. 193-195). Nonetheless, we had previously performed this analysis specifically for XopAL1 and have included this information in the updated manuscript (lns. 439-442).

**Regarding Figure S3, I did not observe a clear reduction in water-soaking symptoms in the Xtt ΔxopAL1 mutant on barley following infiltration, whereas disease development was reduced following spray inoculation (Figure 2). This discrepancy is intriguing and warrants additional investigation. One useful experiment would be to quantify bacterial populations of Xtt WT and the ΔxopAL1 mutant in infiltrated barley leaves, similar to the approach used in Figure S4. If bacterial populations are comparable after infiltration, this may suggest that XopAL1 contributes primarily to early infection processes such as bacterial entry, epiphytic survival, or stomatal invasion rather than post-entry multiplication. This possibility would be consistent with previous findings for XopAP in Xanthomonas oryzae pv. oryzicola (Liu et al., 2022, Journal of Integrative Plant Biology). Findings from this additional experiment will strengthen the mechanistic interpretation.**

We agree with the reviewer’s comment. In fact, we had already quantified Xtt WT and ∆*xopAL1* populations in planta upon leaf infiltration and observed no differences. We also did not observe visual differences in symptom development. However, this type of inoculation only allows for qualitative results of symptom development, and we thus performed spray inoculation as well. We previously decided to not include this experiment because we are currently working to understand the susceptibility and resistance mechanisms mediated by XopAL1 in barley and wheat, respectively.

However, to strengthen this study, we have now included this result in the manuscript (lns. 238-245, now Fig 3) and discussed how XopAL1 could be required during the early infection stage of barley by Xtt (lns. 279-281, and lns. 568-578).

**The manuscript provides convincing evidence for wheat–barley host specificity, which I consider one of its major strengths. However, I find the broader interpretation distinguishing a barley specialist versus a generalist pathogen with extended host range beyond wheat to be less strongly supported by the current data. Although Table S3 is informative, the evidence does not yet appear sufficiently strong to fully support the broader generalist claim, particularly with respect to Emmer wheat, Einkorn wheat, rye, oat, and intermediate wheatgrass. At present, the most robust conclusion is that loss of XopAL1 alters compatibility with wheat-related hosts, rather than establishing a broad generalist phenotype. I therefore recommend either providing stronger experimental support for these additional hosts or tempering the language surrounding the generalist claim and focusing more specifically on XopAL1-mediated wheat/barley host specificity. Additionally, it is interesting that miniTn7::xopAL1 complementation in Xtu did not abolish disease symptoms in hosts other than wheat. This observation deserves further discussion, as it may indicate that XopAL1 alone is insufficient to define host range and likely acts in combination with additional genetic determinants or background-specific virulence factors.**

We agree with the reviewer that our data mostly supports XopAL1 as a determinant of host specificity between wheat-barley, rather than its loss being the sole driver of a generalist phenotype in Xtu. It is well documented that Xtu is a broad host range pathogen from wheat to barley to even ornamental asparagus (doi: 10.1094/phyto-08-20-0337-r, 10.1126/science.44.1134.432, 10.1126/science.50.1280.48, 10.1094/PHYTO.1997.87.11.1111, 10.1094/PDIS-04-19-0797-PDN, 10.1094/pdis-02-21-0407-pdn, 10.1094/phyto-96-0876); while Xtt is largely limited to Hordeum (doi: 10.1126/science.44.1134.432). We therefore view the loss of *xopAL1* as a critical step among likely multiple toward broadening the host range of Xtu in comparison to Xtt. We speculate that this loss is one of several gene gain/loss events that collectively shaped the host range of Xtu. This is supported by our evolutionary analyses of not only *xopAL1*, but of *xopAJ*, *xopE5* and *hopW1* as well, all of which are Xtt-unique effectors. We have revised the manuscript to temper the generalist language and focus on the fact that XopAL1 mediates the host range of *X. translucens* only for wheat. In addition, we believe that the lack of effect of miniTn*7*::*xopAL1* in Xtu inoculated on hosts other than wheat is consistent with the specific role of XopAL1 in triggering resistance in wheat.

**Reviewer #3:**

**1. While the wheat phenotypes in Figs 1-2 were straightforward, the interpretation of the barley fitness/pathogenesis effects were confusing. On L232, the authors set up the remainder of the paper with, "the downstream analyses described here focused mainly on XopAL1 due to its role in determining the ability of X. translucens to cause disease in its two main economic host plants, wheat and barley(17, 24)." I am unclear on the evidence that XopAL1 contributes to disease in barley. In Fig 2A there is no impact of XopAL1 on barley, and in Fig 2BC there is a slight effect on bacterial fitness, though it is not clear to me how this connects to its virulence.**

We thank the reviewer for giving us the opportunity to clarify these phenotypes and highlight how our findings support that XopAL1 impacts both the virulence and fitness of Xtt. In the previous figure 2A we performed a qualitative analysis to observe if disease symptoms are being developed or not. In this figure, we are observing water soaking spots that developed upon spray inoculation. Water soaking is a type of leaf lesion (disease) that appears wet, dark, and translucent. In these figures, we visualized symptoms using a light board placed under leaves as often used by other colleagues (doi: 10.3389/fpls.2019.00507), and thus symptoms appear as lighter spots on leaves due to water soaking being translucent. We have added these descriptions throughout the manuscript to better guide the reader.

Our results demonstrated that deletion of *xopAL1* did not affect pathogenicity of Xtt in barley (ability to cause disease or not) but allowed for host jump to wheat. This is because water soaking symptom still developed when inoculating via both infiltration and spray. However, the density of water soaking spots on barley leaves inoculated via spray with the Xtt ∆*xopAL1* mutant appeared to be lower in comparison to the wild-type. We thus quantified the leaf area displaying symptoms and observed that deletion of this effector gene significantly reduced the mean average of the symptomatic leaf area from 28% (WT) to 14% (mutant; now Fig. 4B). This represents a two-fold reduction in symptoms development, and we thus concluded that XopAL1 affects the virulence of Xtt (in other words, severity of disease).

Xtu produces limited symptoms on barley when inoculated via spray. We therefore could not quantify it. Nonetheless, the goal was to assess the role of XopAL1 in Xtt, since this effector is conserved in the barley-specialist. Due to XopAL1 affecting virulence of Xtt, we quantified the endophytic populations of Xtt and Xtu in barley leaves and observed a significant decrease in the pathogen population whenever this effector was absent (now Fig. 4C). Even though differences in the pathogen population may seem minor (mostly because we are observing results as Log10 of CFU), deletion of *xopAL1* decreased the mean average of the Xtt population by 76%, and introduction of this gene into Xtu increased its population mean average by 313%. We have now added more details and these numbers when narrating these results to better describe the effect of XopAL1 to the pathogen’s virulence and fitness (lns. 260-284). We have also generated a Source Data supporting file containing all the raw data used in our quantitative analyses. We believe this will provide additional information for readers to understand the significant quantitative differences between treatments.

**2. I am skeptical of the statistical groups in Fig 4D, specifically the group assignments of the last three samples. Those should be checked given the spread of samples.**

We have checked our analysis and obtained the same results. This data has a non-normal distribution (Shapiro-Wilk test; W = 0.886, *P* value = 0.00000663) and we thus analyzed it using Kruskal-Wallis rank sum test followed by Wilcoxon rank-sum test. This yielded the statistical significance shown in the figure. As mentioned in the previous comment, we have now added an additional supporting file to our manuscript submission containing all the raw data used in the quantitative analyses. This file has the results from Kruskal-Wallis and Wilcoxon test supporting the statistical significance for these specific results. We have also added to the manuscript narrative a description of the fold change in wheat leaf chlorophyll concentration mediated by XopAL1 presence and absence (lns. 373-378).

**Part III – Minor Issues: Editorial and Data Presentation Modifications**

**Reviewer #1:**

**1. Create a standalone section in the main text related to the results for oats and xopAJ (or take it out and write a separate paper?). There is a ton of data/results in the manuscript about xopAJ affecting virulence and host range related to oats, but it is all in the supplement. As it is, the data are not described in enough detail to really do this work justice. The paragraph starting with line 228 is a bit of an aside and feels out of place. It is revisited later, maybe let the reader know you’ll come back to it, or introduce it in a separate section with the xopAJ/oat results? Similarly, the section describing the deletion results feels out of place and isn’t described in enough detail to follow. Was this already tested and described earlier in the paper (Figure S3?).**

We thank the reviewer for the suggestion. We have re-structured the manuscript to describe the rationale for screening the other Xtt effector mutants and focus on functionally characterizing XopAJ in Xtt only after we have finished describing all the results with XopAL1. Two standalone sections were created for XopAJ in which we added the missing details to the manuscript (lns. 447-522). We believe that this reorganization has improved our study’s narrative.

**2. Move the RNAseq earlier (maybe right after Figure 2) as I don’t think it’s the strongest thing to end on. The RNAseq experiment is a bit descriptive and could be better framed in the context of the literature. Similarly, I don’t think the statement in the abstract about “pathway discovery” is supported by RNAseq. Decrease in expression of genes involved in growth (i.e. photosynthesis) is common to many types of defense responses. Thus, this confirms that there is an immune response, but I don’t think provides any novel info about the type of immune response without genetic follow up.**

We thank the reviewer for this suggestion and have moved the RNA-seq section to appear earlier in the manuscript. We also updated the abstract to better reflect our findings. We performed RNA-seq to understand if the chlorotic non-host response is a type of defense response, and to create an informative pool of host differentially expressed genes to further identify candidate resistance genes responsible for recognizing XopAL1. We agree that, as is, our analysis does not provide targets for pathway discovery. We are currently working on this and have thus edited the manuscript to temper our RNA-seq conclusions. We focused on the fact that we have characterized the non-host response as a robust immune response triggered by effectors (lns. 325-331, lns.378-380, and lns. 587-596). This is important because the non-host response remains mostly undescribed and we provide an important initial step toward understanding it.

**3. Provide context from the literature about the type of immunity you think might be happening. In rice Xoo is recognized by a PRR (not an NLR) Ax21, and so I would expect something more akin to pattern-triggered immunity to be going on here, assuming that like the raxX peptide, the effector is being recognized by a PRR not an NLR. Regardless, many PRR and NLR recognition of effectors result in loss of growth, so having some of this context would help. I also think it’s one of the weaker/more descriptive findings and so doesn’t feel like the thing to end on.**

As mentioned in our response to the previous comment, we have an ongoing project that includes further analyzing and mining our RNA-seq data to identify potential candidate resistance genes that recognize XopAL1 and trigger a defense response in wheat. Thus, we prefer to not include this information in this study, since it will be the focus of a future manuscript. However, we included some brief information in our discussion section to highlight how the RNA-seq data can be useful to allow us to identify the resistance gene, whether it is a PRR or NLR (lns 579-596).

**4. Ensure figure 2 is complete with quantification of images and CFU for all treatments. This is needed to support the claim of expansion of host range into barley. Some of these data are already in the supplement (Fig S4), but since this is the central finding of the paper, it would be better to put them in a main figure.**

We have moved figure S4 to the main text and added results for endophytic populations in barley obtained via leaf infiltration (now Fig 3). All other quantification of images and CFU have also been provided. We have also provided an additional supporting file containing the source (raw) data for all of our quantitative analyses to support our findings. We did not quantify water soaking symptoms of barley leaves inoculated with Xtu via spray because only few symptoms developed, and our focus was to assess the role of XopAL1 in Xtt.

**5. Move Figure S3 to the main text and ensure it is fully described in the results. The logic in lines 173-177 is hard to follow without referring to the supplemental figure, and is a bit brief to describe the extent of what was done. Before describing the results, please describe the experiment, hypothesis, ALL the mutants and strains tested, and expected outcome. In this same paragraph, please describe the results for all effectors; it sounds like loss of function of only 1 of the 5 changed host phenotypes, but this is only shown in the figure and not described in the text. Similarly, I would suggest separating the results for the Xtu-specific effector into a separate paragraph. I understand this was a screen but it’s also important data to support the main conclusions that shouldn’t be buried in the supplement.**

We edited our manuscript according to the reviewer’s comments (lns. 193-237). We appreciate the suggestions and believe that this part of the manuscript has substantially improved. **Additional points for clarification/organization:
Line 170-2. This sentence seems to imply the type of immune response is novel and distinct from the described immune responses. First please clarify if it is truly novel (i.e. physiologically or molecularly distinct from ETI and PTI) versus if it hasn’t been described, but might be similar to canonical ETI and PTI. More specifically, how is it distinct from HR? Is this similar or different to Ax21 recognition of raxT? How is it different than HR? Is it PTI (i.e. is there ROS burst or callose deposition? Or haven’t these things been tested.**

The chlorotic non-host response is triggered by effectors, as demonstrated in our study and others (i.e., doi: 10.3389/fpls.2016.01796). However, it differs from the canonical ETI as there is no programmed cell death leading to localized necrosis and killing of the pathogen. In other words, no HR develops. In fact, Xtt still survives and grows in wheat when inoculated via infiltration, as demonstrated in our manuscript. However, Xtt fails to induce disease symptoms, and its endophytic growth is significantly lower than that of the compatible wheat pathogen Xtu. There is not much description of this non-host response in the literature, such as ROS burst, callose deposition, or the molecular pathways leading to it. To address this gap and determine whether this is a true immune/resistance response, we carried out RNA-seq analysis. We added a more detailed description to the manuscript to guide the readers and highlight our contribution to the understanding of a non-host response. We specifically kept the description of the chlorosis phenotype succinct when initially describing it (lns. 198-205) to provide enough information for the reader to understand our screening process. Then, we provided more information about it in the RNA-seq section to support this experiment and highlight the knowledge gap that we addressed (lns. 317-331, and lns. 592-596). We are not sure at this moment if XopAL1 recognition by wheat is similar to Xa21 recognition of RaxX: 1) we are currently working to determine what wheat component recognizes XopAL1 (R protein, guard, decoy, pattern recognition receptor, etc…); and 2) we do not know if the Xa21-mediated immunity is similar to the non-host response studied here, as we could not find description of a chlorotic phenotype in rice in the literature (doi: 10.1094/MPMI.2004.17.6.602, 10.1111/mpp.12368, 10.1126/sciadv.1500245, 10.1038/srep12165).

**I think the hypothesis that hosts might recognize effectors should be introduced earlier (first introduced at line 196), likely in the intro or when T3E are first described in the results. I think the hypothesis that loss of an effector expands host range makes a lot of sense in this context as classical “avirulence factors”, but as virulence factors the logic is harder to follow.**

We have described this concept in the introduction of our manuscript, as suggested by the reviewer (lns. 126-130).

**Additional minor comments
Line 146: “However, 146 specialist Xtt has a larger T3E repertoire than generalist Xtu (Wilcoxon rank-sum test; P value = 147 3.436 e-06).” Please include the number of T3Es and reference the appropriate figure or table.**

Done.

**Avoid/limit adverbs in the results. For instance, “curiously” is used to start consecutive sentences (line 221, and 224)**

We have limited the use of adverbs, as suggested.

**Reviewer #2:**

**Line72 “have significant effects on host health outcomes”**

Done.

**Line 120 “but at a fitness cost”**

Done.

**Line 194-195 “but it restricts”**

Done.

**Line 285-286 “xopAJ originated in Xtt”**

Done.

**Line 315 “285 DEGs”**

Done.

**Line 361 “Bacteria commonly cause disease”**

Done.

**Line 385 “contributes to virulence”**

Done.

**Overall, this is a strong manuscript with important findings, and addressing these points would further improve its impact and clarity.**

We thank the reviewer for the suggestions and kind comments toward our study.

**Reviewer #3:**

**3. Provide additional description of "neighborhoods of xopAL1" in the Results text to explain what these are (syntenic gene arrangements).**

We provided the information requested by the reviewer to clarify what these genomic neighborhoods are, and how we obtained and analyzed them (lns. 386-392).

**4. Fig 3A shows "xopAL1 loss and replacement" but the Results text only discusses the loss. Additionally the figures and text do not provide sufficient resolution to explain what the replacement gene(s) are, whether those replacement gene(s) are conserved across the Xtu clade, or whether there are DNA sequences that suggest how the loss/replacement was accomplished.**

We have added this information to the manuscript (lns. 417-424). While we do not know whether these acquired genes affect host range, or their evolutionary origin, this information helps to understand how the evolution of this genomic locus is dynamic.

**5. The authors assessed three lineages of Xtt as to their ability to serve as wheat pathogens if experimentally deleted for xopAL1, and all three were confirmed to exhibit the wheat-pathogenic phenotype if they lacked the effector. The authors wrote, "Xtt lineages are globally important in barley production systems(39), and all have an inherent pathogenic capacity in wheat production systems based on xopAL1 loss." This is very interesting to think about, and it prompted two questions. First, do these host species coexist frequently in the field? Second, do they authors have ideas as to why this evolutionary event, gene loss of xopAL1, has occurred only once?**

We thank the reviewer for the thoughtful comment and questions. Regarding the first question, barley and wheat indeed coexist in the field. They share overlapping global geographic distributions (i.e. across the U.S. great plains, Europe, and Australia) being frequently cultivated at the same time, used in regional crop rotations, or grown in adjacent fields. We have briefly added this description to the manuscript to clarify this ecological overlap (lns. 619-621).

About the second question, we hypothesize that the single loss of *xopAL1* in Xtu means that this event was ancestral to this clade, possibly leading to a founder effect. There is possibly no strong selective pressure for Xtt to repeatedly lose *xopAL1* because this subgroup is already highly adapted to barley, and the wheat niche is now occupied by Xtu. Nonetheless, Xtt has an inherent risk to jump hosts based on inactivation of single genes. We have updated the Discussion section to include this (lns. 621-626).

**6. Data on the gain of xopAJ in barley are entirely in the supplement. I would strongly recommend making a main manuscript figure of these data, perhaps pulling from Fig S12 and S16. This result is key to the model (Fig. 5), for example, necessitating the work be in the main manuscript.**

We have moved these data (and figures) to the main manuscript, as recommended.

**7. The supplemental material is large and not synthesized for the reader. The above suggestion partially addresses this issue. Can raw data in the supplement be further processed/consolidated to be more readily accessible (e.g., the raw clinker results in Fig S13 & 14)? In their current forms, many of the supplemental figures do not support the key claims in the paper without the reader having to do extensive work to find the relevant elements.**

We moved additional figures from the supporting data to the main manuscript and consolidated figures as much as possible. We have also removed the previous S6 and S7 figures as they were not adding much information than what was already shown in the previous Figure 3B (*xopAL1* locus neighborhood tree). We thank the reviewer for these suggestions.

**8. Wording suggestions:
- For the non plant expert, the chlorosis described on L169-172 is unexpected, yet is critical to understand that section of the paper. It is unexpected both that the phenotype shown is protective, and also that a T3SS effector would contribute to protection. Additional context to explain how/why the chlorosis is, or correlates with a protective response, would make this section easier to understand. I found the reveal/hypothesis later fascinating, but I struggled initially to get a handle on what I was seeing/reading so that I could proceed past this point of the manuscript.**

We have added details throughout the manuscript to guide the reader on what the leaf phenotypes represent (i.e. water soaking is disease and chlorosis is a non-host response related to defense) (lns. 158-159, and lns. 199-205). We also added information about how effectors play a role both in susceptibility and resistance. Briefly, effectors secreted by pathogens disrupt plant processes and pathways that lead to susceptibility, but if they are recognized by components of the plant immune system, usually resistance (R) proteins, an immune response is triggered (lns. 126-130).

**- L187 "pathogenic" seems out of place or missing subsequent words.**

We have restructured this sentence to avoid confusion (lns. 270-272).

**- L225 Suggest rewording "did not convert this subgroup into a non-pathogen of Triticale"**

Done.

**- L227 "DD sub-genome" is unclear. Has this been introduced?**

We have moved this information to the Discussion section (lns. 597-608). We provided more details on the genomic organization of wheat and how our results suggest that resistance is mostly encoded in the DD sub-genome. Briefly, common wheat has an allohexaploid genome (2n = 6x = 42; BBAADD genome) originated from the hybridization of allotetraploid wheat (*Triticum turgidum*; BBAA genome) with *Aegilops tauschii* (DD genome). Our results showed that diploid (AA genome) and allotetraploid (BBAA genome) wheat, as well as the wheat-rye hybrid Triticale (BBAARR, where R is the sub-genome inherited from rye), were overall more susceptible to Xtt independent of XopAL1. Collectively, this suggests that resistance against Xtt may be mostly encoded by the DD sub-genome.

**- L286 "neighborhood 2" refers to the neighborhood in Fig S12 but is unrelated to neighborhood 2 in Fig 3. Could a clearer numbering be used for this; something like "AJ neighborhood 2"?**

We thank the reviewer for the suggestion. We have update our narrative to highlight when we are referring to the *xopAL1* or *xopAJ* locus neighborhoods. We tried to update our figures to reflect this different nomenclature but could not do it due to lack of space. We are using circles with numbers to represent each neighborhood in our locus trees, and the circles would get too big and not fit into the trees if we did this.

**- L309-316. The current manuscript does not state clearly the key finding from Fig 4A that mock samples cluster with the effector-less samples.**

We have added the description of this result to the manuscript (lns. 340-342).
