# Supplementary material for: Effector loss and gain drives host range at a fitness cost
Source: PLoS Pathog. 2026 Aug 3;22(8):e1014145. doi: 10.1371/journal.ppat.1014145 (PMC13472402; doi:10.1371/journal.ppat.1014145)
Supplement: Peer review history

# Author comment: Author response to Decision Letter 0

**DOI:** 10.1371/journal.ppat.1014145.r001

**Transfer Alert**

This paper was transferred from another journal. As a result, its full editorial history (including decision letters, peer reviews and author responses) may not be present.

20 Mar 2026

# Peer review: Decision Letter 0

**DOI:** 10.1371/journal.ppat.1014145.r002

**Authors:** Shou-Wei Ding, Caroline Roper

26 Apr 2026

PPATHOGENS-D-26-00734

Effector loss and gain drives host range at a fitness cost

PLOS Pathogens

Dear Dr. Jacobs,

Thank you for submitting your manuscript to PLOS Pathogens. After careful consideration, we feel that it has merit but does not fully meet PLOS Pathogens's publication criteria as it currently stands. Therefore, we invite you to submit a revised version of the manuscript that addresses the points raised during the review process.

Please submit your revised manuscript by Jun 25 2026 11:59PM. If you will need more time than this to complete your revisions, please reply to this message or contact the journal office at plospathogens@plos.org. When you're ready to submit your revision, log on to https://www.editorialmanager.com/ppathogens/ and select the 'Submissions Needing Revision' folder to locate your manuscript file.

Please include the following items when submitting your revised manuscript:

* A letter that responds to each point raised by the editor and reviewer(s). You should upload this letter as a separate file labeled 'Response to Reviewers'. This file does not need to include responses to any formatting updates and technical items listed in the 'Journal Requirements' section below.

* A marked-up copy of your manuscript that highlights changes made to the original version. You should upload this as a separate file labeled 'Revised Manuscript with Track Changes'.

* An unmarked version of your revised paper without tracked changes. You should upload this as a separate file labeled 'Manuscript'.

If you would like to make changes to your financial disclosure, competing interests statement, or data availability statement, please make these updates within the submission form at the time of resubmission. Guidelines for resubmitting your figure files are available below the reviewer comments at the end of this letter.

We look forward to receiving your revised manuscript.

Kind regards,

Caroline Roper

Guest Editor

PLOS Pathogens

Shou-Wei Ding

Section Editor

PLOS Pathogens

Sumita Bhaduri-McIntosh

Editor-in-Chief

PLOS Pathogens

orcid.org/0000-0003-2946-9497

Michael Malim

Editor-in-Chief

PLOS Pathogens

orcid.org/0000-0002-7699-2064

**Additional Editor Comments :**

This study by Merfa et al is centered about how host-specialize organisms evolve into generalists. They tested the hypothesis that a generalist Xanthomonas translucens subgroup (Xtu) arose from a specialist ancestor (Xtt) via the loss of a single Type III effector encoding gene, xopAL1. Type III effectors have been implicated in host range before, but one reviewer notes that the novelty of this work is the use of natural variation to show that this phenomenon is happening naturally and is driving ecologically and economically important shifts in pathogen host range.

All 3 reviewers recommend revisions to the manuscript focused on restructuring of the manuscript to provide clarity and support to the findings. Reviewers 1 and 3 both suggest that moving some data out of the supplement into the main text would help provide support the main findings of the manuscript. It was also suggested that supplementary material be better synthesized so it is easier to understand the main findings. Reviewers 1 and 3 suggests adding additional explanation/support for the findings in barley.

**Journal Requirements:**

1) We ask that a manuscript source file is provided at Revision. Please upload your manuscript file as a .doc, .docx, .rtf or .tex. If you are providing a .tex file, please upload it under the item type u2018LaTeX Source Fileu2019 and leave your .pdf version as the item type u2018Manuscriptu2019. 2) Please provide an Author Summary. This should appear in your manuscript between the Abstract (if applicable) and the Introduction, and should be 150-200 words long. The aim should be to make your findings accessible to a wide audience that includes both scientists and non-scientists. Sample summaries can be found on our website under Submission Guidelines:https://journals.plos.org/plospathogens/s/submission-guidelines#loc-parts-of-a-submission 3) Please amend your detailed Financial Disclosure statement. This is published with the article. It must therefore be completed in full sentences and contain the exact wording you wish to be published.1) State what role the funders took in the study. If the funders had no role in your study, please state: "The funders had no role in study design, data collection and analysis, decision to publish, or preparation of the manuscript.". 4) Please ensure that the funders and grant numbers match between the Financial Disclosure field and the Funding Information tab in your submission form. Note that the funders must be provided in the same order in both places as well. 5) Please provide a complete Data Availability Statement in the submission form, ensuring you include all necessary access information. If your research concerns only data provided within your submission, please write "All data are in the manuscript and/or supporting information files" as your Data Availability Statement.If your research concerns data from external sources, please amend your Data Availability Statement to include the full link to the data. 6) Some material included in your submission may be copyrighted. According to PLOS’s copyright policy, authors who use figures or other material (e.g., graphics, clipart, maps) from another author or copyright holder must demonstrate or obtain permission to publish this material under the Creative Commons Attribution 4.0 International (CC BY 4.0) License used by PLOS journals. Please closely review the details of PLOS’s copyright requirements here: PLOS Licenses and Copyright. If you need to request permissions from a copyright holder, you may use PLOS's Copyright Content Permission form. Please respond directly to this email and provide any known details concerning your material's license terms and permissions required for reuse, even if you have not yet obtained copyright permissions or are unsure of your material's copyright compatibility. Once you have responded and addressed all other outstanding technical requirements, you may resubmit your manuscript within Editorial Manager.  Potential Copyright Issues: i) Figures 3D, S3, S5, and S17. Please confirm (a) that you are the photographer; or (b) provide written permission from the photographer to publish the photo(s) under our CC BY 4.0 license. ii) Figures 3C and 5. Please confirm whether you drew the images / clip-art within the figure panels by hand. If you did not draw the images, please provide (a) a link to the source of the images or icons and their license / terms of use; or (b) written permission from the copyright holder to publish the images or icons under our CC BY 4.0 license. Alternatively, you may replace the images with open source alternatives. See these open source resources you may use to replace images / clip-art:- https://commons.wikimedia.org- https://openclipart.org/.

Note: If the reviewer comments include a recommendation to cite specific previously published works, please review and evaluate these publications to determine whether they are relevant and should be cited. There is no requirement to cite these works unless the editor has indicated otherwise.

**Reviewers' Comments:**

Reviewer's Responses to Questions

**Part I - Summary**

Please use this section to discuss strengths/weaknesses of study, novelty/significance, general execution and scholarship.

Reviewer #1: Understanding the genetic factors that determines pathogen host range, and how pathogens evolve to infect new hosts, is critical to predicting and understanding emergent diseases. Using Xanthomonas translucens as a model, Merfa et al. study the genomics of host transitions and found that X. translucens subgroup arose from a specialist ancestor via the loss of a single effector gene, xopAL1. Deleting barley-specialist X. translucens xopAL1 was sufficient to result in a gain of virulence on wheat; however, loss of xopAL1 results in a decrease in virulence in barley highlighting an evolutionary tradeoff.

This study advances our understanding of host range and the evolution of new pathogens. While T3E have previously been shown to control host range and fitness tradeoffs between hosts, and even generalist/specialist transitions, I think the novelty of this work is the use of natural variation to show that this phenomenon is happening naturally and is driving ecologically and economically important shifts in pathogen host range.

I think the findings are strong, but the manuscript suffers a bit from trying to keep the main text brief with too many major findings buried in the supplement. There are also a lot of tangents that aren’t described in the abstract or the main summary. I don’t believe Plos Pathogens is restricted to 4 figures; thus I would strongly encourage the authors to reorganize and pull some of the data out of the supplement and put it in the main text. I think this would help tell a stronger and clearer story.

Reviewer #2: This manuscript presents strong and compelling evidence supporting the role of XopAL1 in host specificity between barley and wheat, and overall the study is well designed, experimentally rigorous, and of clear significance to the field of plant–microbe interactions. The combination of comparative genomics, mutant analysis, and infection assays provides an important contribution toward understanding how effector evolution shapes host adaptation in Xanthomonas translucens.

Reviewer #3: This manuscript by Merfa, Jacobs, and colleagues trace loss of Type III secretion effector XopAL1 as a key evolutionary step in development of generalist plant pathogens in the Xanthomonas translucens group. Type III secretion systems are known to be of widespread importance in plant pathogens broadly and Xanthomonas specifically, and this work derived from phylogenomic analysis to examine so-called non-TAL effectors in about a dozen generalist (Xtu) and barley-specialized (Xtt) strains. Loss of XopAL1 promotes pathogenesis in wheat (as shown by engineered loss in Xtt or introduction into the Xtu background). The work also shows that Xtu strains lacking XopAL1 have a fitness defect in barley, demonstrating a tradeoff to loss of this effector that inhibits fitness in barley while enhancing fitness in wheat (although I raise a question about this phenotype). The work also examines an effector gained in Xtt, xopAJ, and demonstrates a significant albeit milder specificity phenotype in that context.

The paper overall is strong in combining multiple approaches to yield a rich understanding of the interplay of bacterial effectors across evolution and how those effectors act in different bacterial-host contexts. The authors are to be applauded for delving into a wide strain collection to ask these questions, for testing their hypotheses in multiple isolates, and for distilling complicated ideas into compelling figures that capture the complexity of the work while still telling the story. My critical comments fall into two categories. First, the writing uses substantial jargon (plant pathogen, plant, genomics jargon, etc), and although I am deeply interested in the topic, it took me much longer to read as I had to dissect through these areas. I pointed out some of these issues, but overall I would recommend reevaluating much of the manuscript for places where additional context could guide the reader. Similarly, the key access point to the work is a double-negative phenotype (the chlorosis phenotype that correlates with protection) that could similarly use more background. I have a similar comment about making the data in the supplement more accessible to the reader. Second, understanding some of the key phenotypes was obscured and it was difficult to tease apart whether these were issues with the writing or the data/interpretation themselves. Point #1 below on the key phenotypes in barley was the most relevant concern. The comments below elaborate in more detail.

**********

**Part II – Major Issues: Key Experiments Required for Acceptance**

Please use this section to detail the key new experiments or modifications of existing experiments that should be absolutely required to validate study conclusions.

Generally, there should be no more than 3 such required experiments or major modifications for a "Major Revision" recommendation. If more than 3 experiments are necessary to validate the study conclusions, then you are encouraged to recommend "Reject".

Reviewer #1: None

Reviewer #2: Lines 151–152 may benefit from further clarification, where the manuscript refers to the “presence” and “absence” of effectors. The definition of “presence” should be expanded beyond simple gene detection. Specifically, it would be helpful to include information regarding amino acid sequence identity, potential truncations, and structural integrity of the predicted effectors. In addition, the authors may wish to discuss whether SNP-level variation within conserved effectors could contribute to host specificity, as even minor sequence changes may alter recognition or virulence functions.

Regarding Figure S3, I did not observe a clear reduction in water-soaking symptoms in the Xtt ΔxopAL1 mutant on barley following infiltration, whereas disease development was reduced following spray inoculation (Figure 2). This discrepancy is intriguing and warrants additional investigation. One useful experiment would be to quantify bacterial populations of Xtt WT and the ΔxopAL1 mutant in infiltrated barley leaves, similar to the approach used in Figure S4. If bacterial populations are comparable after infiltration, this may suggest that XopAL1 contributes primarily to early infection processes such as bacterial entry, epiphytic survival, or stomatal invasion rather than post-entry multiplication. This possibility would be consistent with previous findings for XopAP in Xanthomonas oryzae pv. oryzicola (Liu et al., 2022, Journal of Integrative Plant Biology). Findings from this additional experiment will strengthen the mechanistic interpretation.

The manuscript provides convincing evidence for wheat–barley host specificity, which I consider one of its major strengths. However, I find the broader interpretation distinguishing a barley specialist versus a generalist pathogen with extended host range beyond wheat to be less strongly supported by the current data. Although Table S3 is informative, the evidence does not yet appear sufficiently strong to fully support the broader generalist claim, particularly with respect to Emmer wheat, Einkorn wheat, rye, oat, and intermediate wheatgrass. At present, the most robust conclusion is that loss of XopAL1 alters compatibility with wheat-related hosts, rather than establishing a broad generalist phenotype. I therefore recommend either providing stronger experimental support for these additional hosts or tempering the language surrounding the generalist claim and focusing more specifically on XopAL1-mediated wheat/barley host specificity. Additionally, it is interesting that miniTn7::xopAL1 complementation in Xtu did not abolish disease symptoms in hosts other than wheat. This observation deserves further discussion, as it may indicate that XopAL1 alone is insufficient to define host range and likely acts in combination with additional genetic determinants or background-specific virulence factors.

Reviewer #3: 1. While the wheat phenotypes in Figs 1-2 were straightforward, the interpretation of the barley fitness/pathogenesis effects were confusing. On L232, the authors set up the remainder of the paper with, "the downstream analyses described here focused mainly on XopAL1 due to its role in determining the ability of X. translucens to cause disease in its two main economic host plants, wheat and barley(17, 24)." I am unclear on the evidence that XopAL1 contributes to disease in barley. In Fig 2A there is no impact of XopAL1 on barley, and in Fig 2BC there is a slight effect on bacterial fitness, though it is not clear to me how this connects to its virulence.

2. I am skeptical of the statistical groups in Fig 4D, specifically the group assignments of the last three samples. Those should be checked given the spread of samples.

**********

**Part III – Minor Issues: Editorial and Data Presentation Modifications**

Please use this section for editorial suggestions as well as relatively minor modifications of existing data that would enhance clarity.

Reviewer #1: 1. Create a standalone section in the main text related to the results for oats and xopAJ (or take it out and write a separate paper?). There is a ton of data/results in the manuscript about xopAJ affecting virulence and host range related to oats, but it is all in the supplement. As it is, the data are not described in enough detail to really do this work justice. The paragraph starting with line 228 is a bit of an aside and feels out of place. It is revisited later, maybe let the reader know you’ll come back to it, or introduce it in a separate section with the xopAJ/oat results? Similarly, the section describing the deletion results feels out of place and isn’t described in enough detail to follow. Was this already tested and described earlier in the paper (Figure S3?).

2. Move the RNAseq earlier (maybe right after Figure 2) as I don’t think it’s the strongest thing to end on. The RNAseq experiment is a bit descriptive and could be better framed in the context of the literature. Similarly, I don’t think the statement in the abstract about “pathway discovery” is supported by RNAseq. Decrease in expression of genes involved in growth (i.e. photosynthesis) is common to many types of defense responses. Thus, this confirms that there is an immune response, but I don’t think provides any novel info about the type of immune response without genetic follow up.

3. Provide context from the literature about the type of immunity you think might be happening. In rice Xoo is recognized by a PRR (not an NLR) Ax21, and so I would expect something more akin to pattern-triggered immunity to be going on here, assuming that like the raxX peptide, the effector is being recognized by a PRR not an NLR. Regardless, many PRR and NLR recognition of effectors result in loss of growth, so having some of this context would help. I also think it’s one of the weaker/more descriptive findings and so doesn’t feel like the thing to end on.

4. Ensure figure 2 is complete with quantification of images and CFU for all treatments. This is needed to support the claim of expansion of host range into barley. Some of these data are already in the supplement (Fig S4), but since this is the central finding of the paper, it would be better to put them in a main figure.

5. Move Figure S3 to the main text and ensure it is fully described in the results. The logic in lines 173-177 is hard to follow without referring to the supplemental figure, and is a bit brief to describe the extent of what was done. Before describing the results, please describe the experiment, hypothesis, ALL the mutants and strains tested, and expected outcome. In this same paragraph, please describe the results for all effectors; it sounds like loss of function of only 1 of the 5 changed host phenotypes, but this is only shown in the figure and not described in the text. Similarly, I would suggest separating the results for the Xtu-specific effector into a separate paragraph. I understand this was a screen but it’s also important data to support the main conclusions that shouldn’t be buried in the supplement.

Additional points for clarification/organization:

Line 170-2. This sentence seems to imply the type of immune response is novel and distinct from the described immune responses. First please clarify if it is truly novel (i.e. physiologically or molecularly distinct from ETI and PTI) versus if it hasn’t been described, but might be similar to canonical ETI and PTI. More specifically, how is it distinct from HR? Is this similar or different to Ax21 recognition of raxT? How is it different than HR? Is it PTI (i.e. is there ROS burst or callose deposition? Or haven’t these things been tested.

I think the hypothesis that hosts might recognize effectors should be introduced earlier (first introduced at line 196), likely in the intro or when T3E are first described in the results. I think the hypothesis that loss of an effector expands host range makes a lot of sense in this context as classical “avirulence factors”, but as virulence factors the logic is harder to follow.

Additional minor comments

Line 146: “However, 146 specialist Xtt has a larger T3E repertoire than generalist Xtu (Wilcoxon rank-sum test; P value = 147 3.436 e-06).” Please include the number of T3Es and reference the appropriate figure or table.

Avoid/limit adverbs in the results. For instance, “curiously” is used to start consecutive sentences (line 221, and 224)

Reviewer #2: Line72 “have significant effects on host health outcomes”

Line 120 “but at a fitness cost”

Line 194-195 “but it restricts”

Line 285-286 “xopAJ originated in Xtt”

Line 315 “285 DEGs”

Line 361 “Bacteria commonly cause disease”

Line 385 “contributes to virulence”

Overall, this is a strong manuscript with important findings, and addressing these points would further improve its impact and clarity.

Reviewer #3: 3. Provide additional description of "neighborhoods of xopAL1" in the Results text to explain what these are (syntenic gene arrangements).

4. Fig 3A shows "xopAL1 loss and replacement" but the Results text only discusses the loss. Additionally the figures and text do not provide sufficient resolution to explain what the replacement gene(s) are, whether those replacement gene(s) are conserved across the Xtu clade, or whether there are DNA sequences that suggest how the loss/replacement was accomplished.

5. The authors assessed three lineages of Xtt as to their ability to serve as wheat pathogens if experimentally deleted for xopAL1, and all three were confirmed to exhibit the wheat-pathogenic phenotype if they lacked the effector. The authors wrote, "Xtt lineages are globally important in barley production systems(39), and all have an inherent pathogenic capacity in wheat production systems based on xopAL1 loss." This is very interesting to think about, and it prompted two questions. First, do these host species coexist frequently in the field? Second, do they authors have ideas as to why this evolutionary event, gene loss of xopAL1, has occurred only once?

6. Data on the gain of xopAJ in barley are entirely in the supplement. I would strongly recommend making a main manuscript figure of these data, perhaps pulling from Fig S12 and S16. This result is key to the model (Fig. 5), for example, necessitating the work be in the main manuscript.

7. The supplemental material is large and not synthesized for the reader. The above suggestion partially addresses this issue. Can raw data in the supplement be further processed/consolidated to be more readily accessible (e.g., the raw clinker results in Fig S13 & 14)? In their current forms, many of the supplemental figures do not support the key claims in the paper without the reader having to do extensive work to find the relevant elements.

8. Wording suggestions:

- For the non plant expert, the chlorosis described on L169-172 is unexpected, yet is critical to understand that section of the paper. It is unexpected both that the phenotype shown is protective, and also that a T3SS effector would contribute to protection. Additional context to explain how/why the chlorosis is, or correlates with a protective response, would make this section easier to understand. I found the reveal/hypothesis later fascinating, but I struggled initially to get a handle on what I was seeing/reading so that I could proceed past this point of the manuscript.

- L187 "pathogenic" seems out of place or missing subsequent words.

- L225 Suggest rewording "did not convert this subgroup into a non-pathogen of Triticale"

- L227 "DD sub-genome" is unclear. Has this been introduced?

- L286 "neighborhood 2" refers to the neighborhood in Fig S12 but is unrelated to neighborhood 2 in Fig 3. Could a clearer numbering be used for this; something like "AJ neighborhood 2"?

- L309-316. The current manuscript does not state clearly the key finding from Fig 4A that mock samples cluster with the effector-less samples.

**********

PLOS authors have the option to publish the peer review history of their article (what does this mean?). If published, this will include your full peer review and any attached files.

If you choose “no”, your identity will remain anonymous but your review may still be made public.

**Do you want your identity to be public for this peer review?**  For information about this choice, including consent withdrawal, please see our Privacy Policy.

Reviewer #1: No

Reviewer #2: **Yes:** Quan Zeng

Reviewer #3: No

[NOTE: If reviewer comments were submitted as an attachment file, they will be attached to this email and accessible via the submission site. Please log into your account, locate the manuscript record, and check for the action link "View Attachments". If this link does not appear, there are no attachment files.]

**Figure resubmission:**

While revising your submission, we strongly recommend that you use PLOS’s NAAS tool (https://ngplosjournals.pagemajik.ai/artanalysis) to test your figure files. NAAS can convert your figure files to the TIFF file type and meet basic requirements (such as print size, resolution), or provide you with a report on issues that do not meet our requirements and that NAAS cannot fix.

After uploading your figures to PLOS’s NAAS tool - https://ngplosjournals.pagemajik.ai/artanalysis, NAAS will process the files provided and display the results in the "Uploaded Files" section of the page as the processing is complete. If the uploaded figures meet our requirements (or NAAS is able to fix the files to meet our requirements), the figure will be marked as "fixed" above. If NAAS is unable to fix the files, a red "failed" label will appear above. When NAAS has confirmed that the figure files meet our requirements, please download the file via the download option, and include these NAAS processed figure files when submitting your revised manuscript.

**Reproducibility:**

To enhance the reproducibility of your results, we recommend that authors of applicable studies deposit laboratory protocols in protocols.io, where a protocol can be assigned its own identifier (DOI) such that it can be cited independently in the future. Additionally, PLOS ONE offers an option to publish peer-reviewed clinical study protocols. Read more information on sharing protocols at https://plos.org/protocols?utm_medium=editorial-email&utm_source=authorletters&utm_campaign=protocols

# Author comment: Author response to Decision Letter 1

**DOI:** 10.1371/journal.ppat.1014145.r003

22 Jun 2026

Attachment Submitted filename: Response to reviewers.docx

# Editor report: Decision Letter 1

**DOI:** 10.1371/journal.ppat.1014145.r004

**Authors:** Shou-Wei Ding, Caroline Roper

3 Jul 2026

Dear Professor Jacobs,

We are pleased to inform you that your manuscript 'Effector loss and gain drives host range at a fitness cost' has been provisionally accepted for publication in PLOS Pathogens.

Before your manuscript can be formally accepted you will need to complete some formatting changes, which you will receive in a follow up email. A member of our team will be in touch with a set of requests.

Please note that your manuscript will not be scheduled for publication until you have made the required changes, so a swift response is appreciated.

IMPORTANT: The editorial review process is now complete. PLOS will only permit corrections to spelling, formatting or significant scientific errors from this point onwards. Requests for major changes, or any which affect the scientific understanding of your work, will cause delays to the publication date of your manuscript.

Should you, your institution's press office or the journal office choose to press release your paper, you will automatically be opted out of early publication. We ask that you notify us now if you or your institution is planning to press release the article. All press must be co-ordinated with PLOS.

Thank you again for supporting Open Access publishing; we are looking forward to publishing your work in PLOS Pathogens.

Best regards,

Caroline Roper

Guest Editor

PLOS Pathogens

Shou-Wei Ding

Section Editor

PLOS Pathogens

Sumita Bhaduri-McIntosh

Editor-in-Chief

PLOS Pathogens

orcid.org/0000-0003-2946-9497

Michael Malim

Editor-in-Chief

PLOS Pathogens

orcid.org/0000-0002-7699-2064

***********************************************************

The authors have responded to the reviewer's comments in a thorough manner and the paper has integrated the reviewer feeedback in a thoughtful way into the revised manuscript. One minor comment for Figure 7. It would be helpful to label panel A with Oat like panel B is labeled with wheat.

Reviewer Comments (if any, and for reference):

# Editor report: Acceptance letter

**DOI:** 10.1371/journal.ppat.1014145.r005

**Authors:** Shou-Wei Ding, Caroline Roper

Dear Dr. Jacobs,

We are delighted to inform you that your manuscript, "Effector loss and gain drives host range at a fitness cost," has been formally accepted for publication in PLOS Pathogens.

We have now passed your article onto the PLOS Production Department who will complete the rest of the pre-publication process. All authors will receive a confirmation email upon publication.

The corresponding author will soon be receiving a typeset proof for review, to ensure errors have not been introduced during production. Please review the PDF proof of your manuscript carefully, as this is the last chance to correct any scientific or type-setting errors. Please note that major changes, or those which affect the scientific understanding of the work, will likely cause delays to the publication date of your manuscript. Note: Proofs for Front Matter articles (Pearls, Reviews, Opinions, etc...) are generated on a different schedule and may not be made available as quickly.

Soon after your final files are uploaded, the early version of your manuscript, if you opted to have an early version of your article, will be published online. The date of the early version will be your article's publication date. The final article will be published to the same URL, and all versions of the paper will be accessible to readers.

For Research Articles, you will receive an invoice from PLOS for your publication fee after your manuscript has reached the completed accept phase. If you receive an email requesting payment before acceptance or for any other service, this may be a phishing scheme. Learn how to identify phishing emails and protect your accounts at https://explore.plos.org/phishing.

Thank you again for supporting open-access publishing; we are looking forward to publishing your work in PLOS Pathogens.

Best regards,

Sumita Bhaduri-McIntosh

Editor-in-Chief

PLOS Pathogens

orcid.org/0000-0003-2946-9497

Michael Malim

Editor-in-Chief

PLOS Pathogens

orcid.org/0000-0002-7699-2064
